# Renin‐Angiotensin System Inhibitors in Patients With COVID‐19: A Meta‐Analysis of Randomized Controlled Trials Led by the International Society of Hypertension

**DOI:** 10.1161/JAHA.122.026143

**Published:** 2022-08-24

**Authors:** Sonali R. Gnanenthiran, Claudio Borghi, Dylan Burger, Bruno Caramelli, Fadi Charchar, Julio A. Chirinos, Jordana B. Cohen, Antoine Cremer, Gian Luca Di Tanna, Alexandre Duvignaud, Daniel Freilich, D. H. Frank Gommans, Abraham E. Gracia‐Ramos, Thomas A. Murray, Facundo Pelorosso, Neil R. Poulter, Michael A. Puskarich, Konstantinos D. Rizas, Rodolfo Rothlin, Markus P. Schlaich, Michael Schreinlecher, Ulrike Muscha Steckelings, Abhinav Sharma, George S. Stergiou, Christopher J. Tignanelli, Maciej Tomaszewski, Thomas Unger, Roland R. J. van Kimmenade, Richard D. Wainford, Bryan Williams, Anthony Rodgers, Aletta E. Schutte, the COVID‐METARASI Consortium

**Affiliations:** ^1^ The George Institute for Global Health University of New South Wales Sydney NSW Australia; ^2^ Department of Medical and Surgical Sciences University of Bologna Italy; ^3^ Department of Cellular and Molecular Medicine, Kidney Research Centre, Ottawa Hospital Research Institute University of Ottawa Canada; ^4^ Interdisciplinary Medicine in Cardiology Unit, InCor University of Sao Paulo Brazil; ^5^ School of Health and Life Sciences Federation University Australia Ballarat VIC Australia; ^6^ Division of Cardiovascular Medicine University of Pennsylvania Perelman School of Medicine Philadelphia PA; ^7^ Renal‐Electrolyte and Hypertension Division and Department of Biostatistics, Epidemiology, and Informatics University of Pennsylvania Perelman School of Medicine Philadelphia PA; ^8^ Department of Cardiology and Hypertension, Hypertension Excellence Center Hôpital Saint André, Centre Hospitalier Universitaire de Bordeaux & University Bordeaux Bordeaux France; ^9^ Department of Infectious Diseases and Tropical Medicine, Division of Tropical Medicine and Clinical International Health Hôpital Pellegrin, Centre Hospitalier Universitaire de Bordeaux & University Bordeaux Bordeaux France; ^10^ Bassett Medical Center Cooperstown NY; ^11^ Department of Cardiology Radboud University Medical Center Nijmegen The Netherlands; ^12^ Netherlands Heart Institute Utrecht The Netherlands; ^13^ Departamento de Medicina Interna, Hospital General, Centro Médico Nacional “La Raza” Instituto Mexicano del Seguro Social Mexico City Mexico; ^14^ Departamento de Medicina Interna Hospital Regional de Alta Especialidad de Zumpango Estado de Mexico Mexico; ^15^ Division of Biostatistics, School of Public Health University of Minnesota Minneapolis MN; ^16^ Asociacion Argentina de Medicamentos Ciudad Autonoma de Buenos Aires Argentina; ^17^ Servicio de Anatomía Patologica, Hospital de Alta Complejidad El Calafate SAMIC Santa Cruz Argentina; ^18^ Imperial Clinical Trials Unit Imperial College London London UK; ^19^ Department of Emergency Medicine Hennepin County Medical Center University of Minnesota Minneapolis MN; ^20^ Medizinische Klinik und Poliklinik I Ludwig Maximilian University Hospital Munich Munich Germany; ^21^ Sociedad Argentina de Farmacología Clínica, Asociacion Medica Argentina Buenos Aires Argentina; ^22^ Dobney Hypertension Centre, Medical School, Royal Perth Hospital Unit–Royal Perth Hospital Medical Research Foundation University of Western Australia Perth Australia; ^23^ Department of Internal Medicine III, Cardiology and Angiology Medical University of Innsbruck Innsbruck Austria; ^24^ Department of Cardiovascular and Renal Research University of Southern Denmark Odense Denmark; ^25^ Division of Cardiology McGill University Health Centre Montreal Quebec Canada; ^26^ Hypertension Center STRIDE‐7, School of Medicine, Third Department of Medicine, Sotiria Hospital National and Kapodistrian University of Athens Athens Greece; ^27^ Department of Surgery University of Minnesota Minneapolis MN; ^28^ Division of Cardiovascular Sciences, Faculty of Medicine, Biology and Health University of Manchester Manchester UK; ^29^ Manchester Academic Health Science Centre Manchester University National Health Service Foundation Trust Manchester Manchester UK; ^30^ Cardiovascular Research Institute Maastricht–School for Cardiovascular Diseases Maastricht University Maastricht The Netherlands; ^31^ Department of Pharmacology and Experimental Therapeutics and the Whitaker Cardiovascular Institute Boston University School of Medicine Boston MA; ^32^ Institute of Cardiovascular Science University College London and National Institute for Health Research University College London Hospitals Biomedical Research Centre London UK

**Keywords:** acute kidney injury, angiotensin II receptor blockers, angiotensin‐converting enzyme inhibitors, COVID‐19, hypertension, renin‐angiotensin system inhibitors, Hypertension, Pharmacology, ACE/Angiotension Receptors/Renin Angiotensin System, Clinical Studies

## Abstract

**Background:**

Published randomized controlled trials are underpowered for binary clinical end points to assess the safety and efficacy of renin‐angiotensin system inhibitors (RASi) in adults with COVID‐19. We therefore performed a meta‐analysis to assess the safety and efficacy of RASi in adults with COVID‐19.

**Methods and Results:**

MEDLINE, EMBASE, ClinicalTrials.gov, and the Cochrane Controlled Trial Register were searched for randomized controlled trials that randomly assigned patients with COVID‐19 to RASi continuation/commencement versus no RASi therapy. The primary outcome was all‐cause mortality at ≤30 days. A total of 14 randomized controlled trials met the inclusion criteria and enrolled 1838 participants (aged 59 years, 58% men, mean follow‐up 26 days). Of the trials, 11 contributed data. We found no effect of RASi versus control on all‐cause mortality (7.2% versus 7.5%; relative risk [RR], 0.95; [95% CI, 0.69–1.30]) either overall or in subgroups defined by COVID‐19 severity or trial type. Network meta‐analysis identified no difference between angiotensin‐converting enzyme inhibitors versus angiotensin II receptor blockers. RASi users had a nonsignificant reduction in acute myocardial infarction (2.1% versus 3.6%; RR, 0.59; [95% CI, 0.33–1.06]), but increased risk of acute kidney injury (7.0% versus 3.6%; RR, 1.82; [95% CI, 1.05–3.16]), in trials that initiated and continued RASi. There was no increase in need for dialysis or differences in congestive cardiac failure, cerebrovascular events, venous thromboembolism, hospitalization, intensive care admission, inotropes, or mechanical ventilation.

**Conclusions:**

This meta‐analysis of randomized controlled trials evaluating angiotensin‐converting enzyme inhibitors/angiotensin II receptor blockers versus control in patients with COVID‐19 found no difference in all‐cause mortality, a borderline decrease in myocardial infarction, and an increased risk of acute kidney injury with RASi. Our findings provide strong evidence that RASi can be used safely in patients with COVID‐19.

**Nonstandard Abbreviations and Acronyms**
ACE2: angiotensin‐converting enzyme 2
AKI: acute kidney injury
RASi: renin‐angiotensin system inhibitors

**Clinical Perspective**

**What Is New?**

- There was an almost 2‐fold increased risk of acute kidney injurty associated with renin‐angiotensin system inhibitors (RASi) in patients hospitalized with acute COVID‐19 in hospitalized patients (7.0% versus 3.6%; relative risk, 1.82; [95% CI, 1.05–3.16]). The overall event rate was low, but effects were consistent across trials that initiated and those that continued RASi, but was not associated with an increased need for dialysis or mortality at short‐term follow‐up.

**What Are the Clinical Implications?**

- Evidence suggests that patients who are using RASi should continue taking their medication as prescribed; the overall cardiovascular benefits of these drugs are overwhelming, and early alerts of potential increased risk in patients with COVID‐19 have been silenced; similarly, clinicians should not be hesitant to initiate RASi treatment in patients with COVID‐19.
- RASi can still be safely used in patients with COVID‐19 while being aware of an increased risk of acute kidney injury in hospitalized patients.
- There does not appear to be increased risk of acute kidney injury in outpatients, which is where the vast majority of COVID‐19 is managed, and longer term follow‐up is needed to investigate renal outcomes and whether there may even be benefits of RASi to slow the progression of proteinuric chronic kidney disease in such patients.

Renin‐angiotensin system inhibitors (RASi), including angiotensin‐converting enzyme inhibitors (ACEIs) and angiotensin II receptor blockers (ARBs), are the most widely prescribed antihypertensive treatments used by hundreds of millions of people worldwide.^1^ RASi are not only first‐line agents for the treatment of hypertension but also are the cornerstone for treating conditions such as heart failure, coronary heart disease, diabetes, and chronic kidney disease. It has been suggested that RASi therapy may upregulate the expression of the angiotensin‐converting enzyme 2 (ACE2) receptor,^2^, ^3^ which is the functional receptor for SARS‐CoV‐2,^4^ the virus responsible for the COVID‐19 pandemic. However, ACE2 upregulation has not been consistently demonstrated,^5^ nor has it been shown to affect the function of RASi.^6^

The BRACE CORONA (blockers of angiotensin receptor and angiotensin‐converting enzyme inhibitors suspension in hospitalized patients with coronavirus infection) randomized trial^7^ in patients hospitalized with mild–moderate COVID‐19 suggested that days alive outside of hospital were equivalent in those continuing ACEIs/ARBs compared with those who had therapy suspended. Similarly compared with discontinuation of RASi, the REPLACE COVID (the randomized elimination or prolongation of angiotensin converting enzyme inhibitors and angiotensin receptor blockers in coronavirus disease 2019) trial found that continuation of RASi had no effect on a composite global rank score as a marker for COVID‐19 severity.^8^ In comparison, the ACE‐COVID trial demonstrated that RASi discontinuation may lead to a more rapid and improved recovery from COVID‐19.^9^ Most randomized controlled trials (RCTs) starting ARB therapy have also failed to demonstrate any difference compared with those not randomized to RASi therapy.^10^, ^11^, ^12^, ^13^, ^14^, ^15^ These trials, together with multiple others, are small to moderate in size, with many unable to meet their recruitment targets, and are insufficiently powered to answer questions regarding binary clinical end points or subgroup populations. Animal and observational studies have provided conflicting data, including concerns that RASi‐induced upregulation of ACE2 receptor expression may increase viral cell entry, whereas other studies have suggested that therapies may provide protective benefits^2^, ^3^, ^16^ or have no effect on ACE2 expression.^17^ In response to these uncertainties, numerous RCTs have been initiated to determine the short‐term safety of RASi in patients with COVID‐19. International hypertension, cardiology, and nephrology societies have consistently recommended that patients continue RASi therapy during the COVID‐19 pandemic on the basis of the strong and well‐documented evidence on their cardiovascular protective effects, but identified a need for more reliable human data.^18^, ^19^, ^20^, ^21^, ^22^ We therefore performed a meta‐analysis of RCTs in patients with COVID‐19 to assess the safety and efficacy of RASi therapy compared with controls without RASi at short‐term follow‐up.

## Methods

### Meta‐Analysis Design and Selection of Trials

Our meta‐analysis and search strategy were reported in accordance with the Preferred Reporting Items for Systematic Review and Meta‐Analysis for protocol recommendations.^23^ The methods of this review were previously published^24^ and will be outlined here in brief. Using the Cochrane Collaboration guidelines,^25^ electronic searches of MEDLINE (1996–present), EMBASE (1996–present), the Cochrane Central Register of Controlled Trials (most recent edition), and ClinicalTrials.gov were performed in June 2021 to identify RCTs that meet the inclusion criteria.

Trials with the following criteria were included: (1) RCTs recruiting between March 2020 and June 2021, (2) patients aged ≥18 years; (3) laboratory‐confirmed SARS‐CoV‐2 infection, (4) comparison of patients randomly assigned to RASi versus no RASi therapy (this includes trials that investigate continuation versus cessation of RASi among patients currently treated with RASi and trials that report initiation of RASi versus control in those not currently treated with such therapies), (5) findings reported in English, and (6) oral administration of RASi therapies. Two reviewers (S.R.G. and A.E.S.) independently performed study selection, quality assessment, and data extraction. Data extraction included information regarding study design, participants, methods, interventions, and outcome measures. End points were all‐cause mortality, acute myocardial infarction, congestive cardiac failure, venous thromboembolism, hospitalization, admission to intensive care, mechanical ventilation, hypotension requiring inotropes, and acute kidney injury (AKI; defined according to the Kidney Disease Improving Global Outcomes criteria)^26^ at short‐term follow‐up (defined as ≤30 days). Standardized grouped tabular deidentified data were requested from trialists. A quality assessment of each trial was performed by 2 authors (S.R.G. and A.E.S.) using the Cochrane Collaboration risk of bias tool.^25^, ^27^ Each included trial was approved by an institutional review committee, and the participants gave informed consent.

### Statistical Analysis

Trial‐specific outcome data were pooled. For binary outcomes, risk ratios and 95% CIs were estimated. Head‐to‐head meta‐analyses were performed by the Mantel–Haenszel fixed‐effects models,^28^ with key results presented using forest plots. A 2‐tailed *P* value of 5% was used for hypothesis testing. Small study effect was assessed by visual inspection of funnel plots and by formal regression‐based Egger tests.^29^ Quantitative heterogeneity has been explored by prespecified subgroup analyses and fitting univariable meta‐regression with the percentage loss to follow‐up as a fixed‐effect covariate.^24^ A fixed‐effects analysis was used unless there was significant heterogeneity (as evidenced by *I* ^2^ >50% and quantitatively large variation), in which case random‐effects analysis was performed instead.^28^ Sensitivity analyses to account for zero and small counts in some trials were performed using the reciprocal of the sample size of the opposite arm.^30^ To assess the relative efficacy of ACEIs versus ARBs (versus control), we also fitted a frequentist random‐effects network meta‐analysis. We reported resulting rankograms and *P* scores^31^: these allow rank treatments on a continuous scale (with a 0–1 range, the higher the better) and are the frequentist analog of the surface under the cumulative ranking curve.

Analyses were conducted using Review Manager 5.3 software (Copenhagen, The Nordic Cochrane Centre, The Cochrane Collaboration), Comprehensive Meta‐Analysis V3 (Biostat, Englewood, NJ), and the package netmeta in R.^32^

The authors declare that all supporting data are available in the article and its supplemental files.

## Results

**Figure 1:**
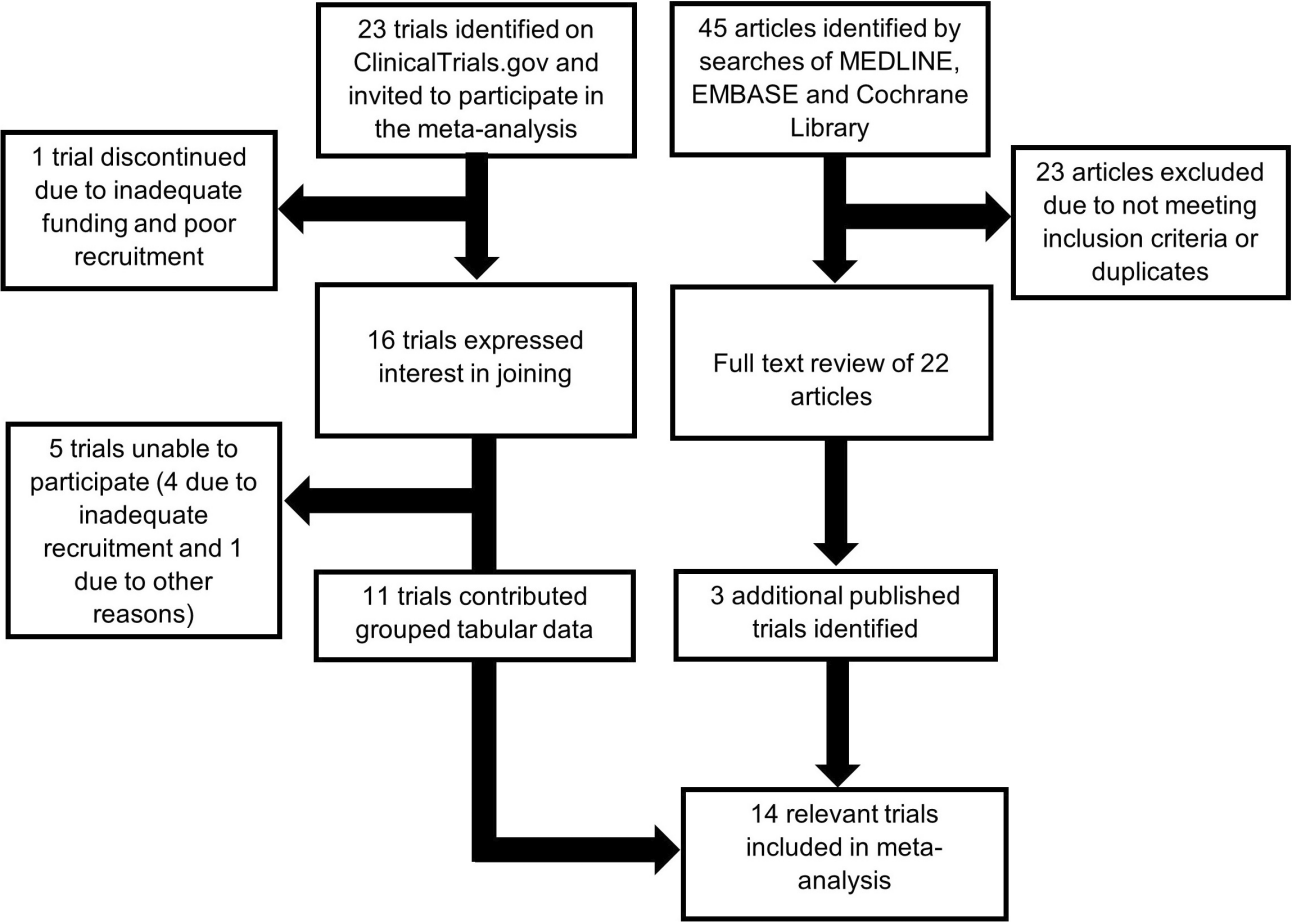
Flowchart of study selection methodology.

Of 45 articles identified through a systematic search and 23 trials on ClinicalTrials.org, 14 RCTs met the inclusion criteria (Table 1, Figure 1). Of the trials, 11 provided grouped tabular data. A total of 1838 patients with a mean follow‐up of 26 days were enrolled, including sites in Argentina, Austria, Brazil, Canada, France, Germany, Iran, Mexico, the Netherlands, and the United States. Of these, 5 trials evaluated the continuation versus discontinuation of RASi therapies in those already on such therapies (n=1079), and 9 trials involved initiation of RASi in those naïve to therapy (n=759). All 9 trials initiating RASi therapies involved commencement of ARBs (n=5 telmisartan, n=3 losartan, n=1 valsartan).

**Table 1:** Characteristics of Included Randomized Controlled Trials of Adults With COVID‐19

Trial name	Country	Inclusion criteria	Intervention	Control	No.	Follow‐up, d
ACEI‐COVID^9^	Germany; Austria	Symptomatic COVID‐19 ACEI/ARB use before admission Hemodynamically stable	Continue ACEI/ARB	Discontinue ACEI/ARB	204	30
BRACE CORONA^7^	Brazil	Hospitalization with COVID‐19 ACEI/ARB use before admission	Continue ACEI/ARB	Discontinue ACEI/ARB	659	30
RAAS‐COVID^15^	Canada	Hospitalization with COVID‐19 ACEI/ARB use before admission	Continue ACEI/ARB	Discontinue ACEI/ARB	46	30
REPLACE‐COVID^8^	United States, Canada, Mexico, Sweden, Peru, Bolivia, and Argentina	Hospitalization with COVID‐19 ACEI/ARB use before admission	Continue ACEI/ARB	Discontinue ACEI/ARB	152	5
SWITCH‐COVID	Brazil	Hospitalization with COVID‐19 Hypertension requiring ACEI/ARB use before admission	Continue ACEI/ARB	Discontinue ACEI/ARB	18	30
ALPS‐COVID IP^14^	United States	Hospitalization with a respiratory SOFA ≥1 and increased oxygen requirement compared with baseline among those on home O_2_	Losartan	Placebo	205	28
ALPS‐COVID OP^13^	United States	Outpatients not requiring hospitalization Symptomatic (within 24 h of informed consent)	Losartan	Placebo	117	28
ARB use to minimize progression to respiratory failure^11^	United States	Mild to moderate hypoxia SpO_2_<96% on ≥L/min O_2_ by nasal cannula but not requiring mechanical ventilation	Losartan	Standard care	31	10
COVERAGE‐France	France	No indication for hospitalization or acute oxygen therapy Age ≥60 years or 50 to 59 years with At least 1 of the following risk factors: hypertension, obesity, diabetes, CAD, CCF, stroke, COPD, CKD, solid tumors, or malignant blood diseases that are progressive or were diagnosed <5 years ago or immunodeficiency	Telmisartan	Vitamin supplement	69	14
COVID MED	United States	Hospitalized patients	Losartan	Placebo	12	30
Evaluation of the effect of losartan in COVID‐19^12^	Iran	Hospitalized patients Hypertension: systolic BP 130 to 140 mm Hg and diastolic BP 85 to 90 mm Hg who were managed by nonpharmacological strategies or were newly diagnosed	Losartan	Amlodipine	80	30
PRAETORIAN‐COVID	The Netherlands	Hospitalized patients	Valsartan	Placebo	23	14
STAR‐COVID	Mexico	Hospitalized with hypoxic respiratory failure: SpO_2_≤94% on room air or tachypnea (respiratory rate≥22 breaths/min)	Telmisartan	Standard care	64	30
Telmisartan for treatment of patients with COVID‐19^10^	Argentina	Hospitalization with COVID‐19 Symptomatic COVID‐19	Telmisartan	Standard care	141	30

ACEI indicates angiotensin‐converting enzyme inhibitor; ACEI‐COVID, the stopping ACE‐inhibitors in COVID‐19 trial; ALPS‐COVID OP, angiotensin receptor blocker based lung protective strategy for COVID‐19 outpatient trial; ALPS‐COVIDIP, Angiotensin receptor blocker based lung protective strategy for COVID‐19inpatient trial; ARB, angiotensin II receptor blocker; BP, blood pressure; BRACE CORONA, blockers of angiotensin receptor and angiotensin‐converting enzyme inhibitors suspension in hospitalized patients with coronavirus infection; CAD, coronary artery disease; CCF, congestive cardiac failure; CKD, chronic kidney disease; COPD, chronic obstructive pulmonary disease; COVERAGE‐France, randomized trial to evaluate the safety and efficacy of outpatient treatments to reduce the risk of worsening in individuals with COVID‐19 with risk factors; COVID MED, comparison of therapeutics for hospitalized patients infected with SARS‐CoV‐2; PRAETORIAN‐COVID, randomized clinical trial with valsartan for prevention of acute respiratory distress syndrome in hospitalized patients with SARS‐COV‐2 Infection Disease; RAAS‐COVID, renin‐angiotensin aldosterone system inhibitors in COVID‐19; REPLACE COVID, the randomized elimination or prolongation of angiotensin converting enzyme inhibitors and angiotensin receptor blockers in coronavirus disease 2019; SOFA, sequential organ failure assessment; STAR‐COVID, telmisartanin respiratory failure due to COVID‐19; and SWITCH‐COVID, switch of renin‐angiotensin system inhibitors in patients with COVID‐19.

### Study Quality

The RCTs were of high quality as assessed by the Cochrane Collaboration risk of bias tool (Table S1, Figure S1). There were 4 placebo‐controlled, double‐blinded RCTs, 9 open‐label trials, and 1 double‐blinded RCT comparing ARB versus amlodipine. Of these, 12 trials were conducted in patients hospitalized with COVID‐19 and 2 trials recruited outpatients. All trials used random sequence generation and were judged as being low risk of selection bias. The double‐blinded trials were judged as being at low risk of allocation concealment and performance biases, whereas the open‐label trials were judged as having moderate risk of these biases. Most trials were at low risk of detection bias and attrition bias, with only 1 trial having a loss to follow‐up of >10%. All trials had a low risk of reporting bias.

### Baseline Clinical Characteristics

Baseline clinical characteristics of the intervention and control groups are described in Table 2, indicating comparable profiles. The mean age of the population was 58.8 years, and 57.6% were men. Hypertension was prevalent in 75.5%, diabetes in 28.5%, cardiovascular disease in 10.4%, obesity in 35.8%, and chronic obstructive pulmonary disease in 10.8%. COVID‐19 severity ranged from mild (46.6%) or moderate (44.2%) to severe (9.2%). Of those patients recruited, 21.6% were either current or past smokers.

**Table 2:** Baseline Clinical Characteristics of Total Cohort (N=1838)

	Renin‐angiotensin system inhibitors (n=917)	Control (n=921)
Mean age, y	58.6	58.9
Sex, n (%)		
Male sex	526/917 (57.4)	532/921 (57.8)
Female sex	391/917 (42.6)	389/921 (42.2)
Past medical history, n (%)		
Hypertension	669/889 (75.3)	679/897 (75.7)
Diabetes	266/917 (29.0)	258/921 (28.0)
Hypercholesterolemia	115/329 (35.0)	94/325 (28.9)
Cardiovascular disease	90/856 (10.5)	89/867 (10.2)
Obesity	164/451 (36.4)	159/450 (35.3)
Chronic kidney disease	48/759 (6.3)	44/763 (5.8)
Chronic obstructive pulmonary disease	64/586 (10.9)	61/572 (10.7)
Smoking, n (%)		
Ever smoked	109/514 (21.2)	113/516 (21.9)
Nonsmoker	405/514 (78.8)	403/516 (78.1)
COVID‐19 severity, n (%)		
Mild	343/722 (47.5)	324/709 (45.7)
Moderate	311/722 (43.0)	321/709 (45.3)
Severe	68/722 (9.4)	64/709 (9.0)

Cardiovascular disease defined as established coronary artery disease, heart failure, arrythmia, and/or stroke; chronic kidney disease defined as estimated glomerular filtration rate <60 mL/min per 1.73 m^2^.

### Primary Outcome

#### All‐Cause Mortality

**Figure 2:**
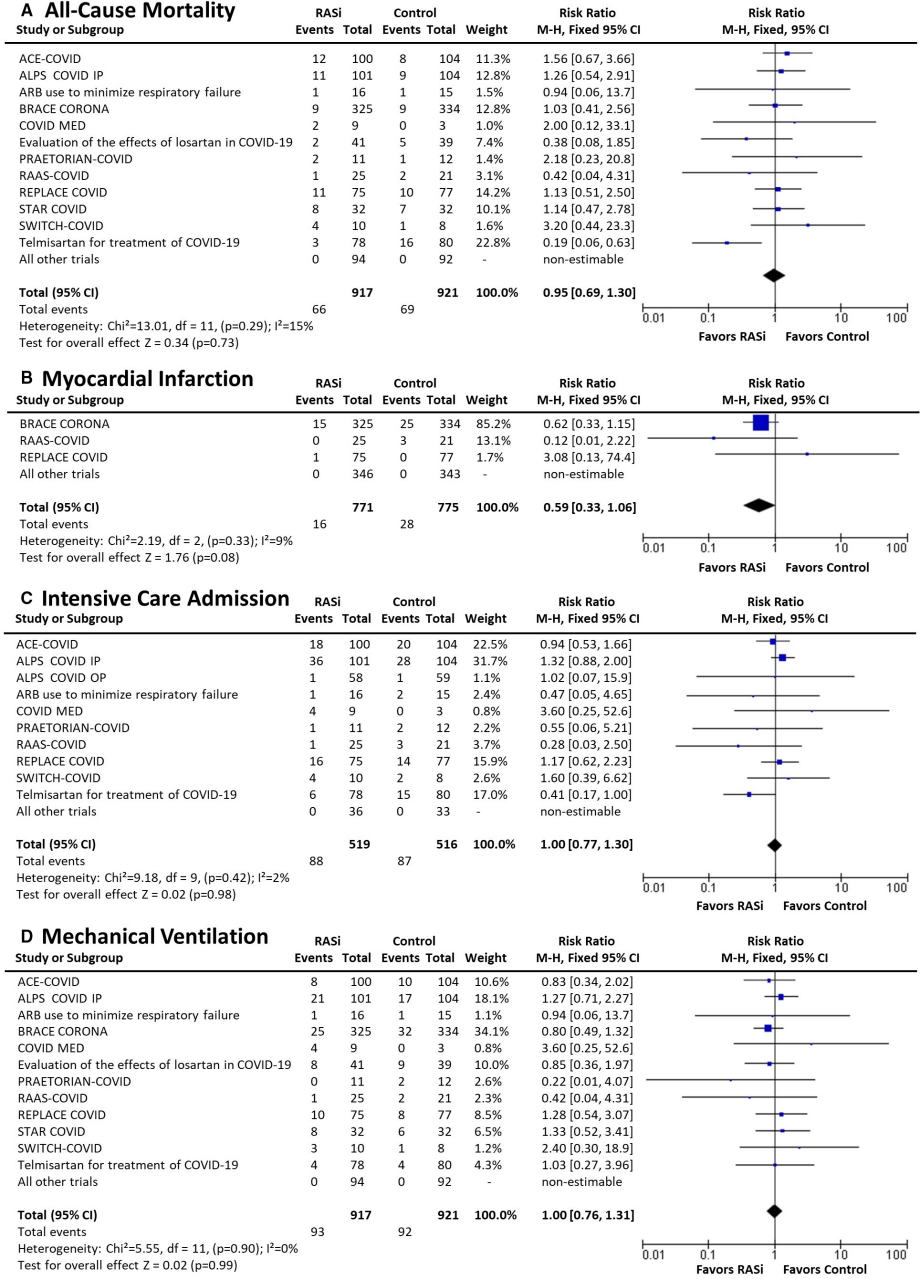
Outcomes at short‐term follow‐up (≤30 days).^7^, ^8^, ^9^, ^10^, ^11^, ^12^, ^13^, ^14^, ^15^ ACEI‐COVID, the stopping ace‐inhibitors in COVID‐19 trial; ALPS‐COVID IP, angiotensin receptor blocker based lung protective strategy for COVID‐19 inpatient trial; ALPS‐COVID OP, angiotensin receptor blocker based lung protective strategy for COVID‐19 outpatient trial; BRACE CORONA, blockers of angiotensin receptor and angiotensin‐converting enzyme inhibitors suspension in hospitalized patients with coronavirus infection; COVERAGE‐France, randomized trial to evaluate the safety and efficacy of outpatient treatments to reduce the risk of worsening in individuals with COVID‐19 with risk factors; COVID MED, comparison of therapeutics for hospitalized patients infected with SARS‐CoV‐2; M‐H indicates Mantel–Haenszel; PRAETORIAN‐COVID, randomised clinical trial with valsartan for prevention of acute respiratory distress syndrome in hospitalised patients with SARS‐COV‐2 infection disease; RAAS‐COVID, renin‐angiotensin aldosterone system inhibitors in COVID‐19 trial; RASi, renin‐angiotensin system inhibitors; REPLACE COVID, the randomized elimination or prolongation of angiotensin converting enzyme inhibitors and angiotensin receptor blockers in coronavirus disease 2019; STAR‐COVID, telmisartan in respiratory failure due to COVID‐19; and SWITCH‐COVID, switch of renin‐angiotensin system inhibitors in patients with COVID‐19.

A total of 14 trials provided all‐cause mortality data (n=1838; Figure 2A), with 12 trials reporting a total of 135 deaths. We found no effect of RASi versus control on all‐cause mortality (7.2% versus 7.5%; relative risk [RR], 0.95; [95% CI, 0.69–1.30]; *I* ^2^=15%; *P*=0.73). When analyzed by trial type, there was no significant difference between trials that compared RASi initiation (RR, 0.72; [95% CI, 0.46–1.14]; *P*=0.16) versus continuation (RR, 1.24; [95% CI, 0.78–1.96]; *P*=0.36; *P*=0.28 for subgroup difference; Figure S2). We also found no difference in mortality by placebo control versus open‐label trials, location of trial, or COVID‐19 severity (Figures S3 through S5). In the ARB class, there was no difference between the different drugs (Figure S6). There was no significant publication bias as assessed by Egger regression testing (*P*=0.86), although inspection of the plot suggested an underrepresentation of trials showing benefit with RASi therapy (Figure S7).

Sensitivity analyses demonstrated that there were no effects on all‐cause mortality across subgroups based on age, sex, or ethnicity (Figures S8 through S10), although there was a nonsignificant trend to increased mortality among the White population with RASi therapy (RR, 1.52; [95% CI, 0.85–2.72]; *P*=0.16). Analyses accounting for the small counts in some trials also did not change the results (Table S2). There were also no between‐group differences in all‐cause mortality for those on RASi compared with control when stratified by the presence or absence of hypertension, diabetes, cardiovascular disease, chronic kidney disease, chronic obstructive pulmonary disease, smoking status, or obesity (Figures S11 through S17). Although the largest trial (BRACE‐CORONA) accounted for a large proportion of participants, an analysis excluding this trial did not change the results (RR, 0.93; [95% CI, 0.66–1.31]). Meta‐regression analysis of trials according to percentage loss to follow‐up demonstrated that trials with a higher loss to follow‐up overestimated mortality benefit with RASi (coefficient, −0.165; [95% CI, −0.281 to −0.050]; *P*=0.005; Figure S18).

Network meta‐analysis comparing control to ACEIs versus ARBs demonstrated no statistically significant differences between ACEIs and ARBs, but ACEIs were associated with a worse mortality effect with a *P* score of 0.089 compared with *P* scores of 0.72 and 0.69 for ARBs and control, respectively (Figures S19 and S20). In particular, we found the RR of ARBs versus ACEIs of 0.60 (95% CI, 0.29–1.23) and the RRs versus placebo for ACEIs and ARBs equal to 1.65 (95% CI, 0.78–3.48) and 0.99 (95% CI, 0.62–1.59), respectively (overall inconsistency *I* ^2^=28.3%; test of homogeneity *P* value=0.15).

### Secondary Outcomes

#### Myocardial Infarction

A total of 10 trials collected acute myocardial infarction outcomes (n=1546; Figure 2B): 3 trials that compared continuation versus discontinuation of RASi in people with preexisting hypertension and/or cardiovascular disease reported a total of 44 events. Pooling of these studies suggest a substantial but nonstatistically significant reduction in acute myocardial infarction with RASi compared with control (2.1% versus 3.6%; RR, 0.59; [95% CI, 0.33–1.06]; *I* ^2^=9%; *P*=0.078).

#### Coronary Revascularization

Data were collected from 8 trials (n=841), but there were no coronary revascularization events reported in the RASi and control groups.

#### Cerebrovascular Accidents

A total of 10 trials provided cerebrovascular outcomes (n=1546; Figure S21), with 2 trials reporting events. A total of 8 cerebrovascular events were observed. There was no significant difference in cerebrovascular events with RASi compared with control (0.6% versus 0.4%; RR, 1.62; [95% CI, 0.43–6.15]; *I* ^2^=0%; *P*=0.48).

#### Congestive Cardiac Failure

A total of 9 trials provided congestive cardiac failure outcomes (n=1341; Figure S22), with 3 trials reporting a total of 41 heart failure events. There were no statistically significant between‐group differences in congestive cardiac failure on RASi compared with control (2.8% versus 3.3%; RR, 0.71; [95% CI, 0.16–3.17]; *I* ^2^=60%; *P*=0.66).

#### Venous Thromboembolism

Data were available from 9 trials (n=1500; Figure S23), with 3 trials reporting 16 venous thromboembolism events. There was no difference in the rate of thromboembolism between the groups (1.2% versus 0.9%; RR, 1.18; [95% CI, 0.45–3.05]; *I* ^2^=0%; *P*=0.74).

#### Hospitalization

There were only 2 small outpatient trials^13^, ^33^ that reported hospitalization rates for COVID‐19 (n=186; Figure S24). A total of 9 hospitalization episodes were observed. There was no significant difference in rates of hospitalization detected between those on RASi compared with control (6.4% versus 3.3%; RR, 1.92; [95% CI, 0.50–7.35]; *I* ^2^=0; *P*=0.34).

#### Intensive Care Admission

A total of 11 trials collected intensive care admission outcomes (n=1035; Figure 2C), with 10 trials reporting a total of 175 admissions. There was no difference in admission to intensive care between those on RASi compared with control (17.0% versus 16.9%; RR, 1.00; [95% CI, 0.77–1.30]; *I* ^2^=2%; *P*=0.98). Analysis comparing trials that commenced versus those that continued/discontinued RASi also did not demonstrate differences in intensive care admission rates (*P*=0.91 for subgroup differences; Figure S25).

#### Mechanical Ventilation

Of the trials, 9 collected outcome data on need for mechanical ventilation (n=1838; Figure 2D), with 6 trials reporting 185 mechanical ventilation events. There was no difference in the rate of mechanical ventilation between people on RASi compared with controls (10.1% versus 10.0%; RR, 1.00; [95% CI, 0.76–1.31]; *I* ^2^=0%; *P*=0.99). Analysis comparing trials that commenced versus those that continued/discontinued RASi also did not demonstrate differences in mechanical ventilation rates (*P*=0.41 for subgroup differences; Figure S26).

#### Hypotension Requiring Inotropes

**Figure 3:**
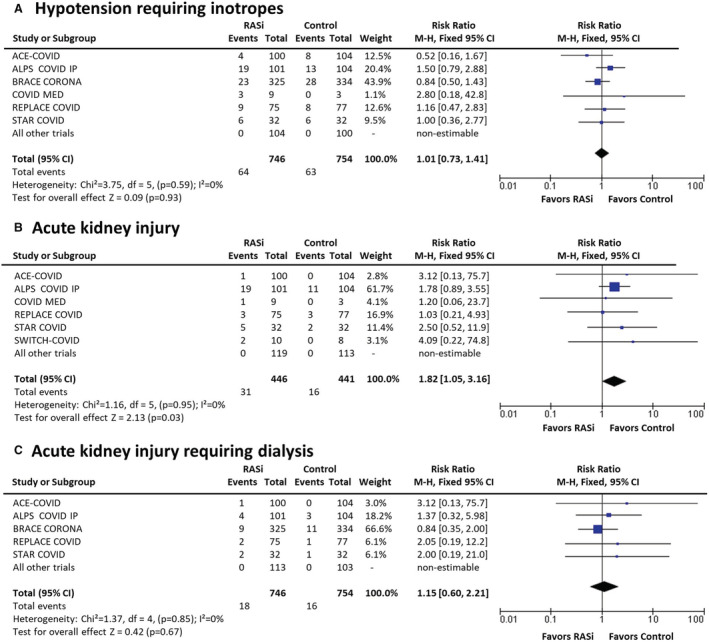
Adverse outcomes at short‐term follow‐up (≤30 days).^7^, ^8^, ^9^, ^10^, ^11^, ^12^, ^13^, ^14^, ^15^ ACEI‐COVID, the stopping ace‐inhibitors in COVID‐19 trial; ALPS‐COVID IP, angiotensin receptor blocker based lung protective strategy for COVID‐19 inpatient trial; ALPS‐COVID OP, angiotensin receptor blocker based lung protective strategy for COVID‐19 outpatient trial; BRACE CORONA, blockers of angiotensin receptor and angiotensin‐converting enzyme inhibitors suspension in hospitalized patients with coronavirus infection; COVERAGE‐France, randomized trial to evaluate the safety and efficacy of outpatient treatments to reduce the risk of worsening in individuals with COVID‐19 with risk factors; COVID MED, comparison of therapeutics for hospitalized patients infected with SARS‐CoV‐2; M‐H indicates Mantel–Haenszel; PRAETORIAN‐COVID, randomised clinical trial with valsartan for prevention of acute respiratory distress syndrome in hospitalised patients with SARS‐COV‐2 infection disease; RAAS‐COVID, renin‐angiotensin aldosterone system inhibitors in COVID‐19 trial; RASi, renin‐angiotensin system inhibitors; REPLACE COVID, the randomized elimination or prolongation of angiotensin converting enzyme inhibitors and angiotensin receptor blockers in coronavirus disease 2019; STAR‐COVID, telmisartan in respiratory failure due to COVID‐19; and SWITCH‐COVID, switch of renin‐angiotensin system inhibitors in patients with COVID‐19.

A total of 9 trials measured hypotension requiring inotropes (n=1500; Figure 3A), with 6 trials reporting a total of 127 events requiring inotropes. In the total group, there was no increase in inotrope use between people on RASi compared with no RASi (8.6% versus 8.4%; RR, 1.01; [95% CI, 0.73–1.41]; *I* ^2^=0%; *P*=0.93). However, sensitivity analyses restricted to patients with severe COVID‐19 demonstrated that RASi was associated with a trend to increased risk of hypotension requiring inotropes compared with controls (33.8% versus 20.3%; RR, 1.56; [95% CI, 0.88–2.79]; *I* ^2^=0%; *P*=0.13; Figure S27). Analysis comparing trials that commenced RASi showed a nonsignificant increase in inotrope use compared with those that continued/discontinued RASi (RR, 1.40 [95% CI, 0.82–2.39] versus RR, 0.84 [95% CI, 0.55–1.28], respectively; *P*=0.15 for subgroup comparison; Figure S28).

#### AKI and Need for Dialysis

A total of 9 trials measured AKI outcomes (n=887; Figure 3B); 6 trials of hospitalized patients reported 47 AKI events. Increased AKI (7.0% versus 3.6%; RR, 1.82; [95% CI, 1.05–3.16]; *I* ^2^=0%; *P*=0.033) was noted in the RASi versus control groups. Although the AKI events were low, this effect was consistent across trials that initiated RASi versus those that continued/discontinued RASi (*P*=0.90 for subgroup differences; Figure S29) and across those with mild, moderate, and severe COVID‐19 (*P*=0.90 for subgroup differences; Figure S30). There was no statistically significant increase in need for dialysis in the RASi group compared with control (2.4% versus 2.1%; RR, 1.15; [95% CI, 0.60–2.21]; *I* ^2^=0%; *P*=0.67; Figure 3C).

## Discussion

In this meta‐analysis of 14 clinical trials in patients with COVID‐19, we found no effect on all‐cause mortality, a trend toward decreased myocardial infarction, and an increased risk of AKI in patients randomly assigned to RASi versus controls. Evidence from RCTs in patient groups without COVID‐19 including those with hypertension and high cardiovascular risk has also indicated an increased risk of AKI from RASi‐based blood pressure (BP) lowering but decreases in vascular events from RASi therapy long term,^34^ suggesting that these effects in patients with COVID‐19 may be real. In this analysis, the safety of RASi was seen across other outcomes, including heart failure, stroke, hospitalization, need for intensive care, and use of inotropes or mechanical ventilation. This is consistent with observational studies that suggested there was no adverse effect of renin‐angiotensin system blockade on COVID‐19 severity and outcomes.^16^, ^35^, ^36^, ^37^, ^38^ The totality of data from this international collaboration provides strong evidence to suggest that RASi can be safely used in patients with COVID‐19 while being aware of an increased risk of AKI, which will better inform public health policy and clinical decision making.

The collective inclusion of data from >1800 patients enabled us to conduct several subgroup analyses. Consistent effects were seen across subgroups. The majority of patients used RASi therapy for the treatment of hypertension, but results in the subgroups with cardiovascular disease and chronic kidney disease were reassuring. Importantly, we were able to demonstrate for the first time that there was no statistically significant difference in ACEI versus ARB use on all‐cause mortality. This suggests that neither the upstream renin‐angiotensin syndrome inhibition by ACEIs nor the downstream inhibition at the receptor level by ARBs influence mortality outcomes in COVID‐19.

We found an almost 2‐fold increased risk of AKI associated with RASi in patients hospitalized with acute COVID‐19 in hospitalized patients, with CIs suggesting a minor to a 4‐fold increase. This risk is a potentially important finding that was unknown before our meta‐analysis.^39^ Effects were consistent across trials that initiated and those that continued RASi,^40^, ^41^, ^42^ but were not associated with increased need for dialysis or mortality at short‐term follow‐up. AKI is common in COVID‐19, with proteinuria often seen in those admitted to hospital,^43^ although the mechanisms appear to be multicausal. Some studies suggest that SARS‐CoV‐2 can directly infect the renal tubular epithelium through an ACE2‐dependent pathway,^40^, ^41^, ^42^, ^44^ whereas others have instead demonstrated acute tubular necrosis, thrombotic microangiopathy, glomerulonephritis, and other intrinsic renal disease.^45^, ^46^, ^47^ Kidney invasion of SARS‐CoV‐2 has been difficult to demonstrate consistently in all studies, and whether it directly leads to AKI is controversial.^48^ There have been reports of virus detected in the kidney by different methods,^49^ but others did not find any such evidence.^48^ The kidneys may be particularly susceptible to SARS‐CoV‐2 because of the high ACE2 expression^50^, ^51^ and coexpression of the cell surface protease facilitating viral cell entry transmembrane serine protease 2 in the proximal tubular cells and tubular progenitor cells.^4^, ^52^ AKI in COVID‐19 can stem from hypovolemia, hypotension, hypoxia, and inflammation or use of different nephrotoxic medications (eg, nonsteroidal anti‐inflammatory drugs) or their combined effects.^53^ It is well recognized that RASi produces reduction in intraglomerular pressure and this can translate into a drop in glomerular filtration rate,^54^ in particular in patients whose baseline kidney function is compromised.^54^ Analyses in patients without COVID‐19^55^, ^56^ have demonstrated that a decline in glomerular filtration rate associated with intensive BP reduction actually preserves blood flow to the renal tubules, a region highly sensitive to hypoxia and susceptible to acute tubular necrosis with sustained hypoperfusion.^57^ Longer term follow‐up is needed to investigate clinical outcomes in patients with a history of COVID‐19 treated with RASi—previous studies in patients without COVID‐19 demonstrated that angiotensin‐converting enzyme inhibition or ARB‐based treatment is associated with lower mortality in the follow‐up after AKI.^58^

We also observed a borderline decrease in acute myocardial infarction with continuation of RASi therapy. The results were driven by the BRACE CORONA trial^7^ (RR, 0.66; [95% CI, 0.33–1.15]), with the addition of 2 smaller trials further confirming this trend in our meta‐analysis (RR, 0.59; [95% CI, 0.33–1.06]; *P*=0.078). These 3 trials all compared continuation versus discontinuation of RASi therapy in people with preexisting hypertension and/or cardiovascular disease. The result is unsurprising given the well‐established benefits afforded by RASi therapy in the reduction in cardiovascular mortality, myocardial infarction, and stroke.^59^, ^60^ One small RCT (n=46) demonstrated that RASi discontinuation increased the incidence of acute heart failure (33% versus 4%; *P*=0.016),^15^ which was consistent with the direction of effect observed in our analysis. The short duration of this analysis did not allow the longer beneficial effects of RASi to be demonstrated. Increased vascular events have been observed with RASi cessation,^61^ with continuation leading to avoidance of drug discontinuation syndromes. The benefits of RASi can take months to accrue, but the risks of withdrawal occur more rapidly.^62^ Our results support the importance of continuing RASi in people with elevated cardiovascular risk—including patients with COVID‐19—consistent with the recommendations of international guidelines.^18^, ^19^, ^20^, ^21^, ^22^

There are a number of limitations to the present analysis. Our meta‐analysis focused on binary clinical end points, and benefits on continuous outcomes (eg, length of stay, duration of ventilation) were not assessed. Visual inspection of the all‐cause mortality funnel plot also suggested an underrepresentation of trials showing benefit with RASi therapy. This is likely to arise from poor recruitment leading to trial termination (NCT04329195), inability to participate in this meta‐analysis because of failure to meet predefined recruitment targets for unblinding (NCT04360551, NCT04351581), or provision of only a low number of participants to the analysis (NCT04335786, NCT04328012, NCT04493359). The relatively low event rates and short follow‐up duration of included trials (≤30 days) also prevents robust assessment of long‐term outcomes. The risk profile of patients included in RCTs may also limit the extrapolation of the results to patient groups in clinical practice who are older and more comorbid. The results also do not evaluate the posological discrimination of the ARBs used in each clinical trial.^63^ Further research is required to assess the mechanism of AKI associated with RASi, rates of renal recovery, and the benefits of RASi for the treatment of proteinuria in these patients and other longer term outcomes. Nevertheless, this is the largest pooled analysis of RCTs compared with other meta‐analyses that were smaller^64^ or included observational studies^65^ and represents a major achievement in international collaboration. This is the most highly powered randomized analysis to assess binary clinical end points and the first to directly compare ACEIs versus ARBs.

This first meta‐analysis of RCTs evaluating RASi versus control in patients with COVID‐19 found no difference in all‐cause mortality, a borderline decrease in myocardial infarction, and an increased risk of AKI with RASi. The risk of AKI was consistent across trials that initiated and those that continued RASi. More evidence is needed with longer term follow‐up to establish the clinical implications of this finding.

## Conclusion

Early controversies that RASi therapy may upregulate the ACE2 receptor and hence pose safety and efficacy issues in patients with COVID‐19 has resulted in several RCTs to be conducted across the globe to address this issue. Our meta‐analysis including 14 RCTs suggests that RASi can be safely used (continued or initiated) in patients with COVID‐19. In those using RASi, we report a trend toward decreased myocardial infarction, with a potential increased risk of AKI—a finding unknown in patients with COVID‐19 before our meta‐analysis. Our inclusion of several trials also enabled the first direct comparison of ACEIs versus ARBs, but our findings indicate no difference. Overall, this meta‐analysis provides strong evidence that RASi can be used safely in patients with COVID‐19, balancing both the benefits and risks on cardiovascular and renal outcomes, respectively.

## Sources of Funding

None.

## Disclosures

Dr Gnanenthiran is supported by a postdoctoral fellowship from the Heart Foundation of Australia. Dr Chirinos has recently consulted for Bayer, Sanifit, Fukuda‐Denshi, Bristol‐Myers Squibb, JNJ, Edwards Life Sciences, Merck, NGM Biopharmaceuticals, and the Galway‐Mayo Institute of Technology. He received University of Pennsylvania research grants from National Institutes of Health, Fukuda‐Denshi, Bristol‐Myers Squibb, Microsoft, and Abbott. He is named as inventor in a University of Pennsylvania patent for the use of inorganic nitrates/nitrites for the treatment of heart failure and preserved ejection fraction and for the use of biomarkers in heart failure with preserved ejection fraction. He has received payments for editorial roles from the American Heart Association, the American College of Cardiology, and Wiley. He has received research device loans from Atcor Medical, Fukuda‐Denshi, Uscom, NDD Medical Technologies, Microsoft, and MicroVision Medical. Dr Schutte received consultancy fees from Abbott and speaker honoraria from Servier, Sanofi, Sun Pharmaceuticals, Omron, Takeda, and Novartis. Dr Stergiou received consultancy fees and speaker honoraria from Astra‐Zeneca, Menarini, Novartis, Sanofi, and Servier. Dr Schlaich has received consulting fees and/or travel and research support from Medtronic, Abbott, Novartis, Servier, Pfizer, and Boehringer‐Ingelheim. Dr Sharma reports receiving support from the Fonds de Recherche Santé Quebec Junior 1 clinician scholar program, Canada Institute for Health Research (grant 175095), Roche Diagnostics, Boeringer‐Ingelheim, Novartis, and Takeda. Dr Poulter has received financial support from several pharmaceutical companies that manufacture blood pressure–lowering agents, for consultancy fees (Servier), research projects and staff (Servier, Pfizer), and for arranging and speaking at educational meetings (AstraZeneca, Lri Therapharma, Napi, Servier, Sanofi, Eva Pharma, Pfizer, Alkem Laboratories, and Glenmark Pharma). He holds no stocks or shares in any such companies. Dr Williams has received honoraria for lectures on hypertension from Servier, Menarini, Pfizer, Boehringer Ingelheim, and Daiichi Sankyo. The remaining authors have no disclosures to report.

## Supporting information

Data S1
Tables S1–S2
Figures S1–S30
