# Supplementary material for: Renin‐Angiotensin System Inhibitors in Patients With COVID‐19: A Meta‐Analysis of Randomized Controlled Trials Led by the International Society of Hypertension
Source: J Am Heart Assoc. 2022 Aug 24;11(17):e026143. doi: 10.1161/JAHA.122.026143 (PMC9496439; doi:10.1161/JAHA.122.026143)

SUPPLEMENTAL MATERIAL

Data S1. Trials that contributed grouped tabular data

Trial Name	Trial Team
ACEI-COVID	Team: Bauer A, Sappler N, Dolejsi T, Tilg H, Aulinger BA, Weiss G, Bellmann-Weiler R, Adolf C, Wolf D, Pirklbauer M, Graziadei I, Gänzer H, von Bary C, May AE, Wöll E, von Scheidt W, Rassaf T, Duerschmied D, Brenner C, Kääb S, Metzler B, Joannidis M, Kain HU, Kaiser N, Schwinger R, Witzenbichler B, Alber H, Straube F, Hartmann N, Achenbach S, von Bergwelt-Baildon M, von Stülpnagel L, Schoenherr S, Forer L, Embacher-Aichhorn S, Mansmann U, Massberg S<br>Funding: Austrian Science Fund and German Center for Cardiovascular Research.
RAAS-COVID	Team: Elharram M, Ni J, Afilalo J, Flannery A, Ezekowitz JA, Cheng MP, Ambrosy AP, Zannad F, Brophy J, Giannetti N, Bessissow A, Kronfli N, Marelli A. Aziz H, Alqahtani M, Aflaki M, Craig M, Lopes RD, Ferreira JP<br>Funding: McGill Interdisciplinary Initiative in Infection and Immunity (MI4) and the Division of Cardiology at McGill University.
REPLACE-COVID	Team: Hanff TC, William P, Sweitzer N, Rosado-Santander NR, Medina C, Rodriguez-Mori JE, Renna N, Chang TI, Corrales-Medina V, Andrade-Villanueva JF, Barbagelata A, Cristodulo-Cortez R, Díaz-Cucho OA, Spaak J, Alfonso CE, Valdivia-Vega R, Villavicencio-Carranza M, Ayala-García RJ, Castro-Callirgos CA, González-Hernández LA, Bernales-Salas EF, Coacalla-Guerra JC, Salinas-Herrera CD, Nicolosi L, Basconcel M, Byrd JB, Sharkoski T, Bendezú-Huasasquiche LE, Chittams J, Edmonston DL, Vasquez CR<br>Funding: REPLACE COVID Investigators, REPLACE COVID Trial Social Fundraising Campaign, and FastGrants.
SWITCH-COVID	Team: Girardi ACC, Tavares CAM, Cardozo FAM, Betonico GN, de Almeida L<br>Funding: University of Sao Paulo
ALPS-COVID IP	Team: Ingraham NE, Merck LH, Driver BE, Wacker DA, Black LP, Jones AE, Fletcher CV, South AM, Nelson AC, Lewandowski C, Farhat J, Benoit JL, Biros MH, Cherabuddi K, Chipman JG, Schacker TW, Guirgis FW, Voelker HT, Koopmeiners JS, Tignanelli CJ<br>Funding: Bill and Melinda Gates Foundation, NIH
ALPS-COVID OP	Team: Cummins NW, Ingraham NE, Wacker DA, Reilkoff RA, Driver BE, Biros MH, Bellolio F, Chipman JG, Nelson AC, Beckman K, Langlois R, Bold T, Aliota MT, Schacker TW, Voelker HT, Koopmeiners JS<br>Funding: Minnesota Partnership for Biotechnology and Medical Genomics
COVERAGE-France	Team: Malvy D, Anglaret X, Richert L, Wittkop L, Lhomme E, Sitta R, Gelley A, Hardel L, Wallet C, Schwimmer C, Thiebaut R, Onaisi R, Saint-Lary O, Joseph JP, Dupouy J, Gimenez L, Boucaut A, Chastang J, Naccache JM, Piroth L, Binquet C, Lefèvre B, Makinson A, Picot MC, Montoya A, Crantelle L, Molimard M, Bouchet S, de Lamballerie X, Roussillon C, Landman R<br>Funding: Ministère des Solidarités et de la Santé, Agence Nationale de la Recherche, ANRS | Maladies Infectieuses Emergentes, University of Bordeaux
COVID MED	Team: Victory J, Jenkins P, Krupa N, Wheeler J, Vail GM, Riesenfeld E, Cross P, Gilmore C, Huckabone M, Schworm A, Boregowda U, Deshmukh F, Choi Y, Khan A, Gadomski A<br>Funding: Bassett Healthcare, Reid Health, Goshen Health System
PRAETORIAN-COVID	Team: Aarts GWA, Konijnenberg LSF, Mensink FB, Herrmann JJ.<br>Funding: NLHI, the Dutch Heart Foundation, Novartis Pharma and ZonMW grant 10430012010020
STAR-COVID	Team: Ángeles-Duran GY, Flores-Gómez IR, Flores-Martínez E, Valdin-Orozco TI, Pedraza-Hervert C<br>Funding: National Polytechnic Institute, Mexico
Telmisartan for treatment of patients with COVID-19	Team: Duarte M, Nicolosi LN, Salgado MV, Vetulli H, Aquieri A, Azzato F, Castro M, Coyle J, Davolos I, Criado IF, Gregori R, Mastrodonato P, Rubio MC, Sarquis S, Wahlmann F<br>Funding: Facultad de Medicina (Universidad de Buenos Aires, Argentina), Hospital Espa~nol de Buenos Aires (Argentina) and Laboratorio Elea (Argentina)

Table S1. Quality assessment of RCTs – Cochrane Collaboration Risk of Bias Tool.

Trial Name	Random sequence generation (selection bias)	Allocation concealment (selection bias)	Blinding of participants and researchers (performance bias)	Blinding of outcome assessment (detection bias)	Incomplete outcome data (attrition bias)	Selective reporting (reporting bias)	Other
ACEI-COVID	Low risk	High risk	High risk	Low risk	Low risk	Low risk	Open label trial
BRACE-CORONA	Low risk	High risk	High risk	Low risk	Low risk	Low risk	Open label trial
RAAS-COVID	Low risk	High risk	High risk	Unclear risk	Low risk	Low risk	Open label trial
REPLACE-COVID	Low risk	High risk	High risk	Low risk	Low risk	Low risk	Open label trial
SWITCH-COVID	Low risk	High risk	High risk	Low risk	Low risk	Low risk	Open label trial
ALPS-COVID IP	Low risk	Low risk	Low risk	Low risk	Low risk	Low risk	Placebo controlled
ALPS-COVID OP	Low risk	Low risk	Low risk	Low risk	Low risk	Low risk	Placebo controlled
ARB use to minimize progression to respiratory failure	Low risk	High risk	High risk	Unclear risk	Low risk	Low risk	Open label trial
COVERAGE-France	Low risk	High risk	High risk	Low risk	Low risk	Low risk	Open label trial
COVID MED	Low risk	Low risk	Low risk	Low risk	Low risk	Low risk	Placebo controlled
Evaluation of the effect of losartan in COVID-19	Low risk	Low risk	Low risk	Low risk	Low risk	Low risk	Comparator amlodipine rather than placebo
PRAETORIAN-COVID	Low risk	Low risk	Low risk	Low risk	Low risk	Low risk	Placebo controlled
STAR-COVID	Low risk	High risk	High risk	Low risk	Low risk	Low risk	Open label trial
Telmisartan for treatment of patients with COVID-19	Low risk	High risk	High risk	Unclear risk	High risk	Unclear risk	Open label trial loss to follow-up:>10%

Table S2. Sensitivity analyses to account for small counts in trials*

	Original analyses RR, 95%CI	Sensitivity analyses* RR, 95%CI
**Mortality**	RR 0.95 (0.69-1.30), p=0.73	RR 0.95 (0.69-1.30), p=0.73
**Myocardial infarction**	RR 0.59 (0.33-1.06), p=0.08	RR 0.60 (0.24-1.06), p=0.08
**Intensive care admission**	RR 1.00 (0.77-1.30), p=0.98	RR 1.02 (0.78-1.32), p=0.90
**Mechanical ventilation**	RR 1.00 (0.76-1.31), p=0.99	RR 1.02 (0.77-1.35), p=0.90
**Hypotension requiring inotropes**	RR 1.01 (0.73-1.41), p=0.93	RR 1.01 (0.73-1.41), p=0.93
**Acute kidney injury**	RR 1.82 (1.05-3.16), p=0.03	RR 1.82 (1.05-3.14), p=0.03
**Acute kidney injury requiring dialysis**	RR 1.15 (0.60-2.21), p=0.67	RR 1.15 (0.60-2.19), p=0.68

RR relative risk, CI confidence interval

*Sensitivity analysis using the reciprocal of the sample size of the opposite arm to the cells in tables with zeroes

Figure S1. Quality assessment of RCTs – Cochrane Collaboration Risk of Bias Tool.

Study or Subgroup	RASI Total	Control Total	A	B	C	D	E	F	G
ACE-COVID	100	104	+	−	−	+	+	+	+
ALPS COVID IP	101	104	+	+	+	+	+	+	+
ALPS-COVID OP	58	59	+	+	+	+	+	+	+
ARB use to minimize respiratory failure	16	15	+	−	−		+	+	+
BRACE CORONA	325	334	+	−	−	+	+	+	+
COVERAGE France	36	33	+	−	−	+	+	+	+
COVID MED	9	3	+	+	+	+	+	+	+
Evaluation of the effects of losartan in COVID-19	41	39	+	+	+	+	+	+	+
PRAETORIAN-COVID	11	12	+	+	+	+	+	+	+
RAAS-COVID	25	21	+	−	−		+	+	+
REPLACE COVID	75	77	+	−	−	+	+	+	+
STAR-COVID	32	32	+	−	−	+	+	+	+
SWITCH-COVID	10	8	+	−	−	+	+	+	+
Telmisartan for treatment of COVID-19	78	80	+	−	−		−		+
	**917**	**921**							

Risk of bias legend
(**A**) Random sequence generation (selection bias)
(**B**) Allocation concealment (selection bias)
(**C**) Blinding of participants and personnel (performance bias)
(**D**) Blinding of outcome assessment (detection bias)
(**E**) Incomplete outcome data (attrition bias)
(**F**) Selective reporting (reporting bias)
(**G**) Other bias

Figure S2: All-Cause Mortality – Start vs Continue/Discontinue Trials

Study or Subgroup	RASI Events	RASI Total	Control Events	Control Total	Weight	Risk Ratio M-H, Fixed, 95% CI
**Start**						
ALPS COVID IP	11	101	9	104	12.8%	1.26 [0.54, 2.91]
ARB use to minimize respiratory failure	1	16	1	15	1.5%	0.94 [0.06, 13.68]
COVID MED	2	9	0	3	1.0%	2.00 [0.12, 33.10]
Evaluation of the effects of losartan in COVID-19	2	41	5	39	7.4%	0.38 [0.08, 1.85]
PRAETORIAN-COVID	2	11	1	12	1.4%	2.18 [0.23, 20.84]
STAR-COVID	8	32	7	32	10.1%	1.14 [0.47, 2.78]
Telmisartan for treatment of COVID-19	3	78	16	80	22.8%	0.19 [0.06, 0.63]
All other trials	0	94	0	92		–
**Subtotal (95% CI)**		**382**		**377**	**56.9%**	**0.72 [0.46, 1.14]**
Total events	29		39			
Heterogeneity: Chi² = 9.52, df = 6 (P = 0.15); I² = 37%						
Test for overall effect: Z = 1.39 (P = 0.16)						
**Continue/Discontinue**						
ACE-COVID	12	100	8	104	11.3%	1.56 [0.67, 3.66]
BRACE CORONA	9	325	9	334	12.8%	1.03 [0.41, 2.56]
RAAS-COVID	1	25	2	21	3.1%	0.42 [0.04, 4.31]
REPLACE COVID	11	75	10	77	14.2%	1.13 [0.51, 2.50]
SWITCH-COVID	4	10	1	8	1.6%	3.20 [0.44, 23.28]
**Subtotal (95% CI)**		**535**		**544**	**43.1%**	**1.24 [0.78, 1.96]**
Total events	37		30			
Heterogeneity: Chi² = 2.20, df = 4 (P = 0.70); I² = 0%						
Test for overall effect: Z = 0.91 (P = 0.36)						
**Total (95% CI)**		**917**		**921**	**100.0%**	**0.95 [0.69, 1.30]**
Total events	66		69			
Heterogeneity: Chi² = 13.01, df = 11 (P = 0.29); I² = 15%						
Test for overall effect: Z = 0.34 (P = 0.73)						
Test for subgroup differences: Chi² = 2.64, df = 1 (P = 0.28), I² =51%						

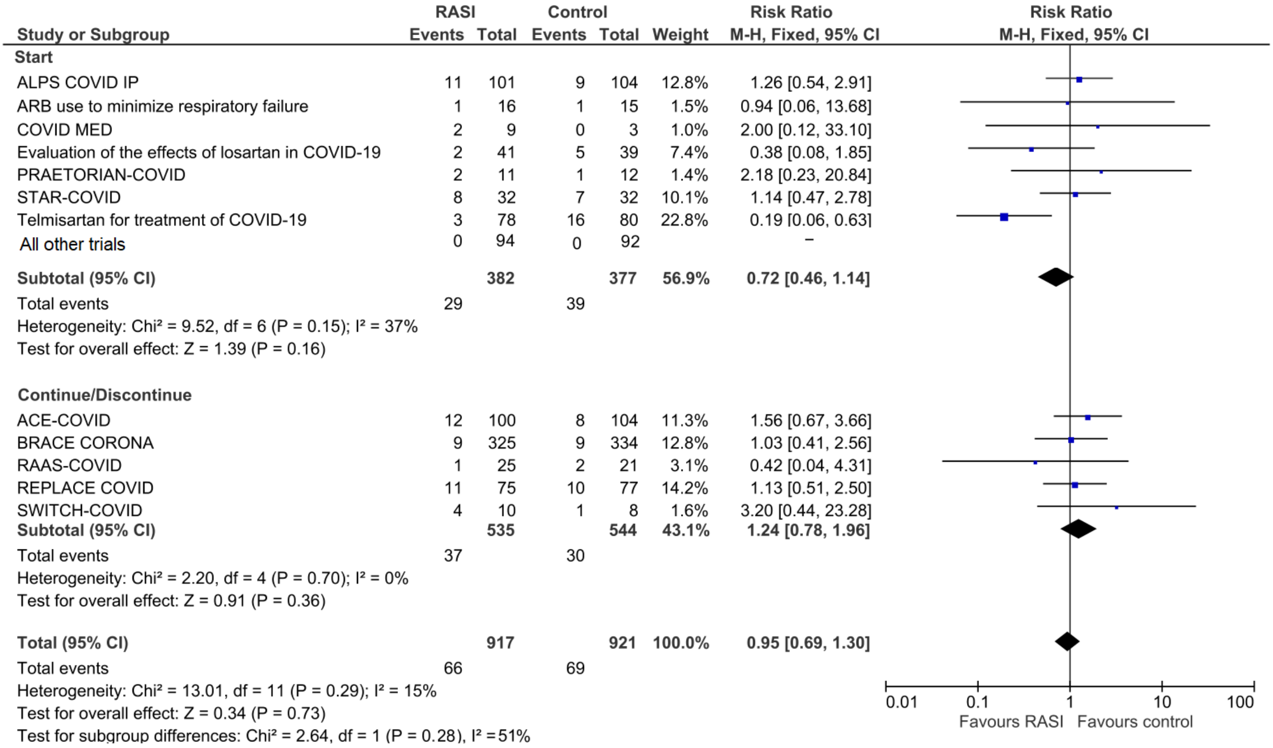

Figure S3: All-Cause Mortality – Placebo Control vs Open Label Trials

Study or Subgroup	RASI Events	RASI Total	Control Events	Control Total	Weight	Risk Ratio M-H, Fixed, 95% CI
**Placebo control trials**						
ALPS COVID IP	11	101	9	104	13.8%	1.26 [0.54, 2.91]
COVID MED	2	9	0	3	1.1%	2.00 [0.12, 33.10]
PRAETORIAN-COVID	2	11	1	12	1.5%	2.18 [0.23, 20.84]
ALPS-COVID OP	0	58	0	59		-
**Subtotal (95% CI)**		**179**		**178**	**16.4%**	**1.39 [0.66, 2.96]**
Total events	15		10			
Heterogeneity: Chi² = 0.27, df = 2 (P = 0.87); I² = 0%						
Test for overall effect: Z = 0.86 (P = 0.39)						
**Open label**						
ACE-COVID	12	100	8	104	12.2%	1.56 [0.67, 3.66]
ARB use to minimize respiratory failure	1	16	1	15	1.6%	0.94 [0.06, 13.68]
BRACE CORONA	9	325	9	334	13.8%	1.03 [0.41, 2.56]
RAAS-COVID	1	25	2	21	3.4%	0.42 [0.04, 4.31]
REPLACE COVID	11	75	10	77	15.4%	1.13 [0.51, 2.50]
STAR-COVID	8	32	7	32	10.9%	1.14 [0.47, 2.78]
SWITCH-COVID	4	10	1	8	1.7%	3.20 [0.44, 23.28]
Telmisartan for treatment of COVID-19	3	78	16	80	24.6%	0.19 [0.06, 0.63]
COVERAGE France	0	36	0	33		-
**Subtotal (95% CI)**		**697**		**704**	**83.6%**	**0.91 [0.63, 1.32]**
Total events	49		54			
Heterogeneity: Chi² = 10.62, df = 7 (P = 0.16); I² = 34%						
Test for overall effect: Z = 0.49 (P = 0.62)						
**Total (95% CI)**		**876**		**882**	**100.0%**	**0.99 [0.71, 1.38]**
Total events	64		64			
Heterogeneity: Chi² = 11.44, df = 10 (P = 0.32); I² = 13%						
Test for overall effect: Z = 0.06 (P = 0.96)						
Test for subgroup differences: Chi² = 0.98, df = 1 (P = 0.32), I² = 0%						

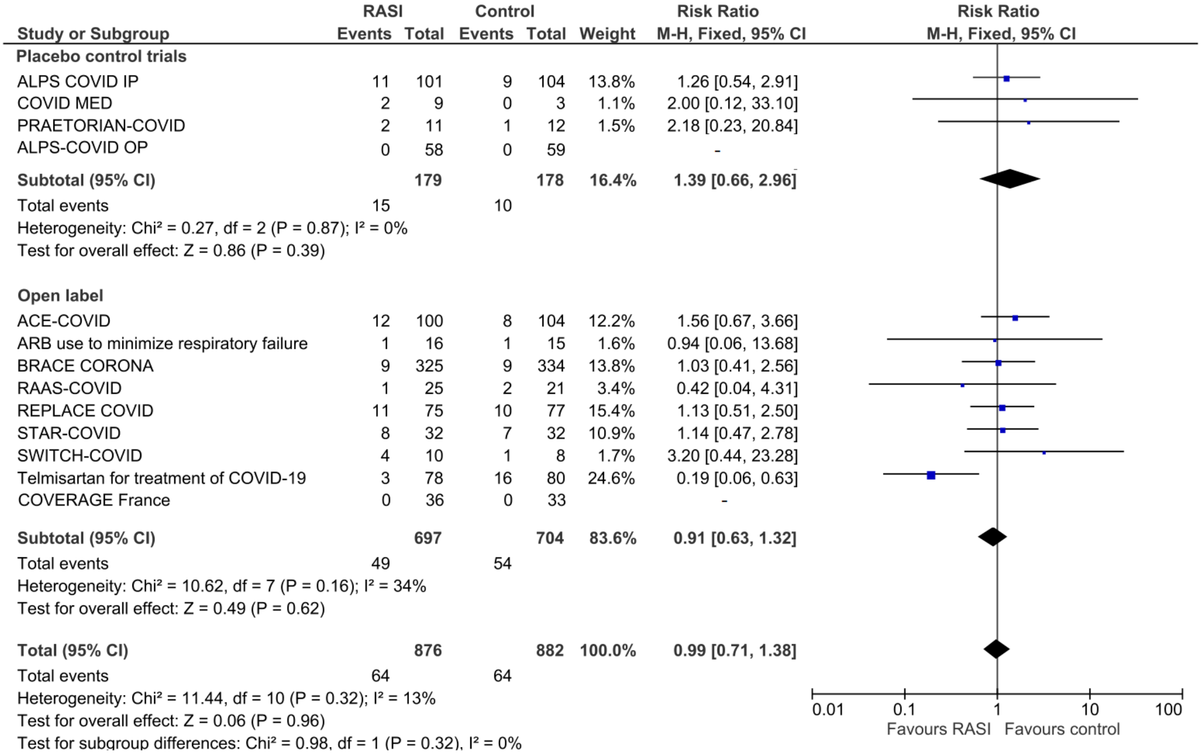

Figure S4: All-Cause Mortality – Trial Location

(A) Random Effect

Study or Subgroup	RASI Events	RASI Total	Control Events	Control Total	Weight	Risk Ratio M-H, Random, 95% CI
**North America**						
ALPS COVID IP	11	101	9	104	15.0%	1.26 [0.54, 2.91]
ALPS-COVID OP	12	100	8	104	14.7%	1.56 [0.67, 3.66]
ARB use to minimize respiratory failure	1	16	1	15	2.1%	0.94 [0.06, 13.68]
COVID MED	2	9	0	3	2.0%	2.00 [0.12, 33.10]
RAAS-COVID	1	25	2	21	2.8%	0.42 [0.04, 4.31]
STAR-COVID	8	32	7	32	13.9%	1.14 [0.47, 2.78]
**Subtotal (95% CI)**		**283**		**279**	**50.5%**	**1.26 [0.78, 2.01]**
Total events	35		27			
Heterogeneity: Tau² = 0.00; Chi² = 1.29, df = 5 (P = 0.94); I² = 0%						
Test for overall effect: Z = 0.95 (P = 0.34)						
**South America**						
BRACE CORONA	9	325	9	334	13.4%	1.03 [0.41, 2.56]
SWITCH-COVID	4	10	1	8	3.8%	3.20 [0.44, 23.28]
Telmisartan for treatment of COVID-19	3	78	16	80	9.0%	0.19 [0.06, 0.63]
**Subtotal (95% CI)**		**413**		**422**	**26.2%**	**0.75 [0.18, 3.21]**
Total events	16		26			
Heterogeneity: Tau² = 1.17; Chi² = 7.54, df = 2 (P = 0.02); I² = 73%						
Test for overall effect: Z = 0.39 (P = 0.70)						
**Europe**						
ACE-COVID	12	100	8	104	14.7%	1.56 [0.67, 3.66]
PRAETORIAN-COVID	2	11	1	12	3.0%	2.18 [0.23, 20.84]
COVERAGE France	0	36	0	33		-
**Subtotal (95% CI)**		**147**		**149**	**17.7%**	**1.63 [0.73, 3.61]**
Total events	14		9			
Heterogeneity: Tau² = 0.00; Chi² = 0.07, df = 1 (P = 0.79); I² = 0%						
Test for overall effect: Z = 1.20 (P = 0.23)						
**Middle-East**						
Evaluation of the effects of losartan in COVID-19	2	41	5	39	5.6%	0.38 [0.08, 1.85]
**Subtotal (95% CI)**		**41**		**39**	**5.6%**	**0.38 [0.08, 1.85]**
Total events	2		5			
Heterogeneity: Not applicable						
Test for overall effect: Z = 1.20 (P = 0.23)						
**Total (95% CI)**		**884**		**889**	**100.0%**	**1.04 [0.70, 1.56]**
Total events	67		67			
Heterogeneity: Tau² = 0.10; Chi² = 13.84, df = 11 (P = 0.24); I² = 21%						
Test for overall effect: Z = 0.19 (P = 0.85)						
Test for subgroup differences: Chi² = 3.02, df = 3 (P = 0.39), I² = 0.8%						

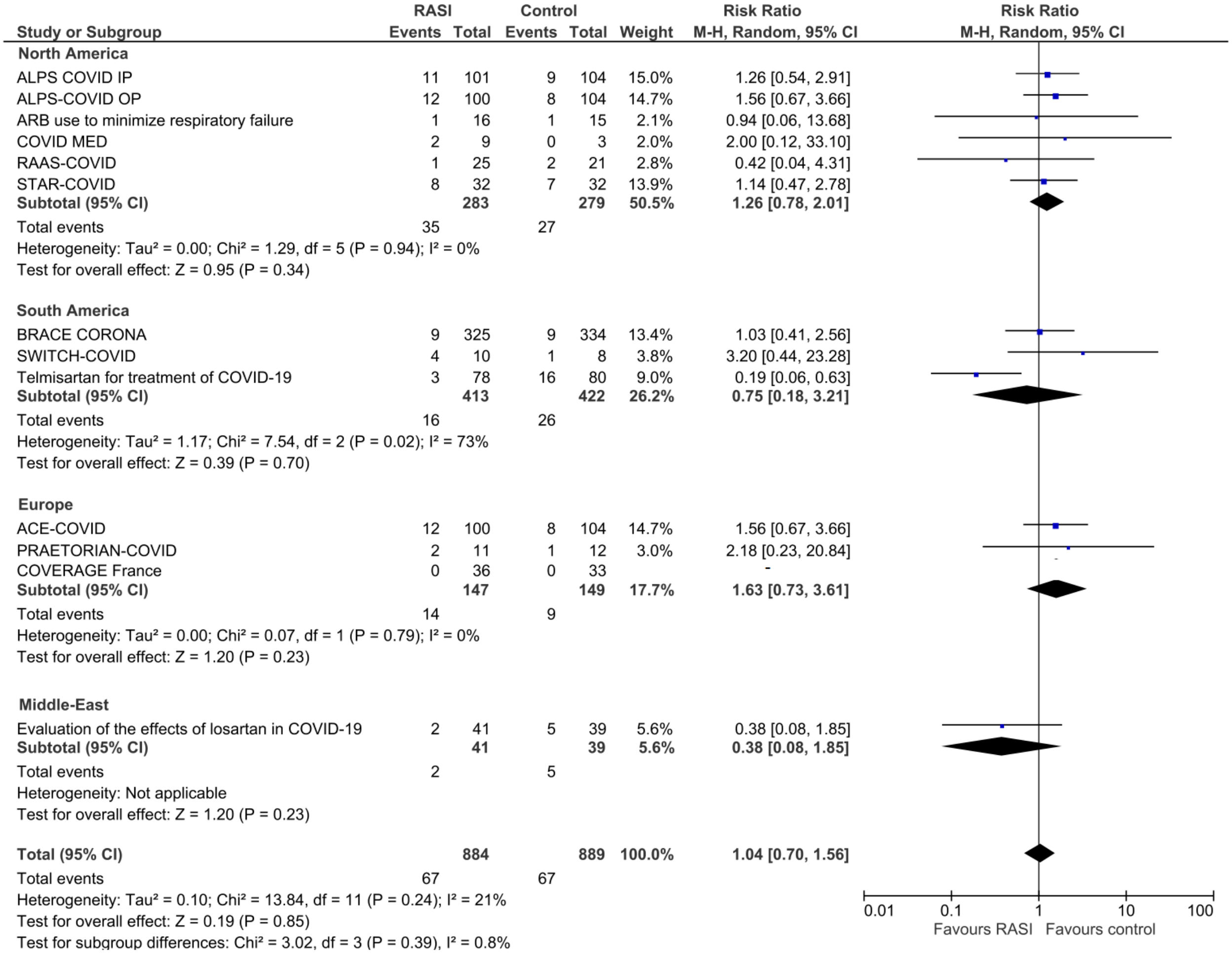

(B) Fixed Effects

Study or Subgroup	RASI Events	RASI Total	Control Events	Control Total	Weight	Risk Ratio M-H, Fixed, 95% CI
**North America**						
ALPS COVID IP	11	101	9	104	13.2%	1.26 [0.54, 2.91]
ALPS-COVID OP	12	100	8	104	11.6%	1.56 [0.67, 3.66]
ARB use to minimize respiratory failure	1	16	1	15	1.5%	0.94 [0.06, 13.68]
COVID MED	2	9	0	3	1.1%	2.00 [0.12, 33.10]
RAAS-COVID	1	25	2	21	3.2%	0.42 [0.04, 4.31]
REPLACE COVID	11	75	10	77		Not estimable
STAR-COVID	8	32	7	32	10.4%	1.14 [0.47, 2.78]
**Subtotal (95% CI)**		**283**		**279**	**41.0%**	**1.26 [0.79, 2.00]**
Total events	35		27			
Heterogeneity: Chi² = 1.29, df = 5 (P = 0.94); I² = 0%						
Test for overall effect: Z = 0.96 (P = 0.34)						
**South America**						
BRACE CORONA	9	325	9	334	13.2%	1.03 [0.41, 2.56]
SWITCH-COVID	4	10	1	8	1.6%	3.20 [0.44, 23.28]
Telmisartan for treatment of COVID-19	3	78	16	80	23.5%	0.19 [0.06, 0.63]
**Subtotal (95% CI)**		**413**		**422**	**38.3%**	**0.61 [0.33, 1.12]**
Total events	16		26			
Heterogeneity: Chi² = 7.54, df = 2 (P = 0.02); I² = 73%						
Test for overall effect: Z = 1.59 (P = 0.11)						
**Europe**						
ACE-COVID	12	100	8	104	11.6%	1.56 [0.67, 3.66]
PRAETORIAN-COVID	2	11	1	12	1.4%	2.18 [0.23, 20.84]
COVERAGE France	0	36	0	33		-
**Subtotal (95% CI)**		**147**		**149**	**13.1%**	**1.63 [0.73, 3.61]**
Total events	14		9			
Heterogeneity: Chi² = 0.07, df = 1 (P = 0.79); I² = 0%						
Test for overall effect: Z = 1.20 (P = 0.23)						
**Middle-East**						
Evaluation of the effects of losartan in COVID-19	2	41	5	39	7.6%	0.38 [0.08, 1.85]
**Subtotal (95% CI)**		**41**		**39**	**7.6%**	**0.38 [0.08, 1.85]**
Total events	2		5			
Heterogeneity: Not applicable						
Test for overall effect: Z = 1.20 (P = 0.23)						
**Total (95% CI)**		**884**		**889**	**100.0%**	**0.99 [0.72, 1.37]**
Total events	67		67			
Heterogeneity: Chi² = 13.84, df = 11 (P = 0.24); I² = 21%						
Test for overall effect: Z = 0.06 (P = 0.95)						
Test for subgroup differences: Chi² = 6.31, df = 3 (P = 0.10), I² = 52.5%						

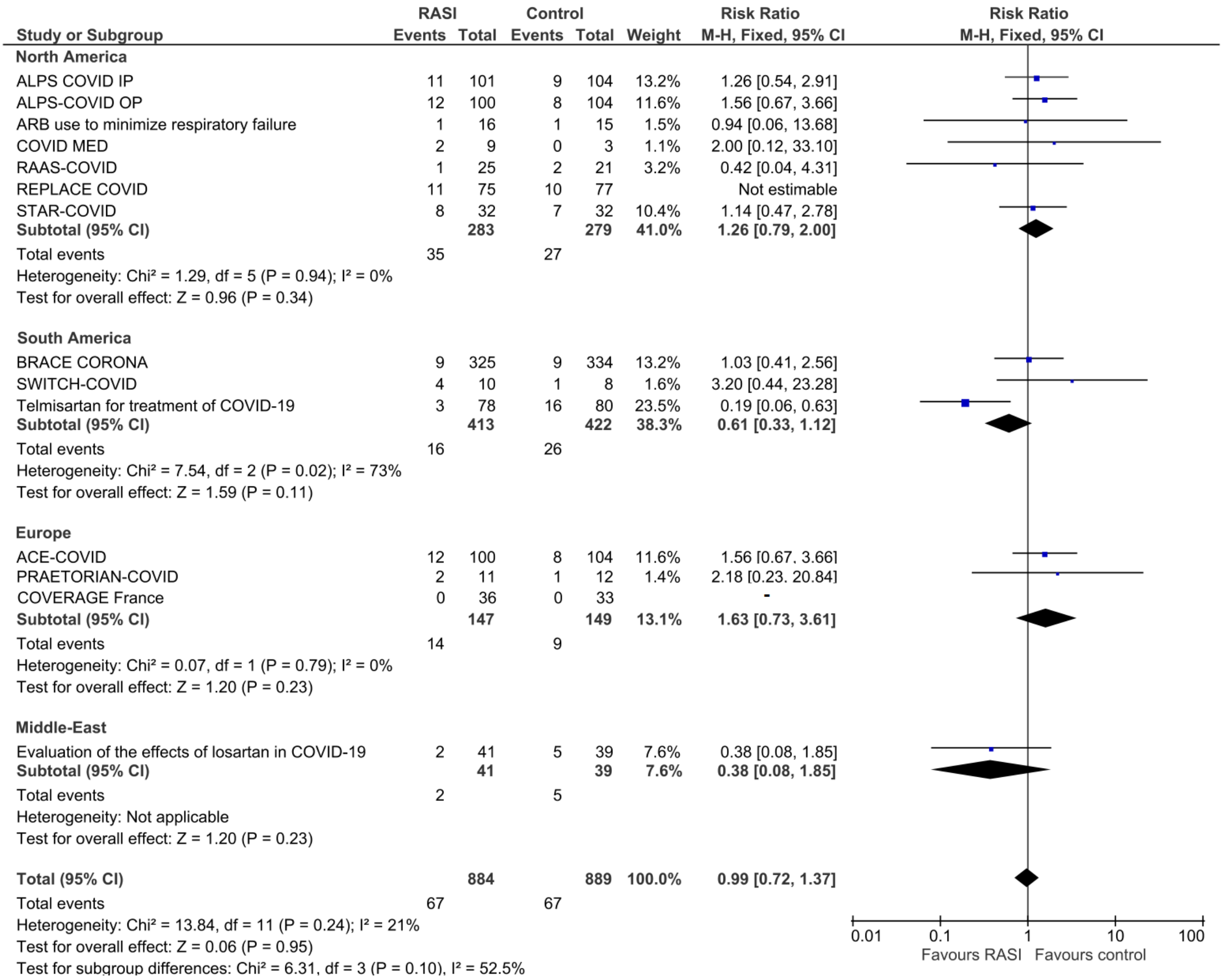

Figure S5: All-Cause Mortality – Severity of COVID-19

	RASI		Control			Risk Ratio
**Study or Subgroup**	**Events**	**Total**	**Events**	**Total**	**Weight**	**M-H, Fixed, 95% CI**
**Mild**						
REPLACE COVID	6	38	5	42	9.1%	1.33 [0.44, 4.00]
Telmisartan for treatment of COVID-19	1	23	3	25	5.5%	0.36 [0.04, 3.24]
All other trials	1	66	0	68		-
**Subtotal (95% CI)**		**127**		**135**	**14.6%**	**0.96 [0.37, 2.50]**
Total events	8		8			
Heterogeneity: Chi² = 1.09, df = 1 (P = 0.30); I² = 8%						
Test for overall effect: Z = 0.08 (P = 0.94)						
**Moderate**						
ACE-COVID	12	100	8	104	15.0%	1.56 [0.67, 3.66]
ALPS COVID IP	6	79	4	83	7.5%	1.58 [0.46, 5.38]
PRAETORIAN-COVID	2	11	1	12	1.8%	2.18 [0.23, 20.84]
REPLACE COVID	2	28	1	25	2.0%	1.79 [0.17, 18.52]
SWITCH-COVID	4	10	1	7	2.2%	2.80 [0.39, 20.02]
Telmisartan for treatment of COVID-19	2	47	13	46	25.1%	0.15 [0.04, 0.63]
All other trials	0	4	0	3		-
**Subtotal (95% CI)**		**279**		**280**	**53.6%**	**0.98 [0.60, 1.63]**
Total events	28		28			
Heterogeneity: Chi² = 10.10, df = 5 (P = 0.07); I² = 51%						
Test for overall effect: Z = 0.06 (P = 0.95)						
**Severe**						
ALPS COVID IP	5	22	5	21	9.8%	0.95 [0.32, 2.83]
COVID MED	1	5	0	1	1.4%	1.00 [0.06, 15.99]
REPLACE COVID	3	9	4	10	7.2%	0.83 [0.25, 2.76]
STAR-COVID	8	32	7	32	13.4%	1.14 [0.47, 2.78]
**Subtotal (95% CI)**		**68**		**64**	**31.8%**	**1.01 [0.56, 1.81]**
Total events	17		16			
Heterogeneity: Chi² = 0.18, df = 3 (P = 0.98); I² = 0%						
Test for overall effect: Z = 0.03 (P = 0.98)						
**Total (95% CI)**		**474**		**479**	**100.0%**	**0.99 [0.69, 1.41]**
Total events	53		52			
Heterogeneity: Chi² = 11.35, df = 11 (P = 0.41); I² = 3%						
Test for overall effect: Z = 0.06 (P = 0.95)						
Test for subgroup differences: Chi² = 0.01, df = 2 (P = 1.00), I² = 0%						

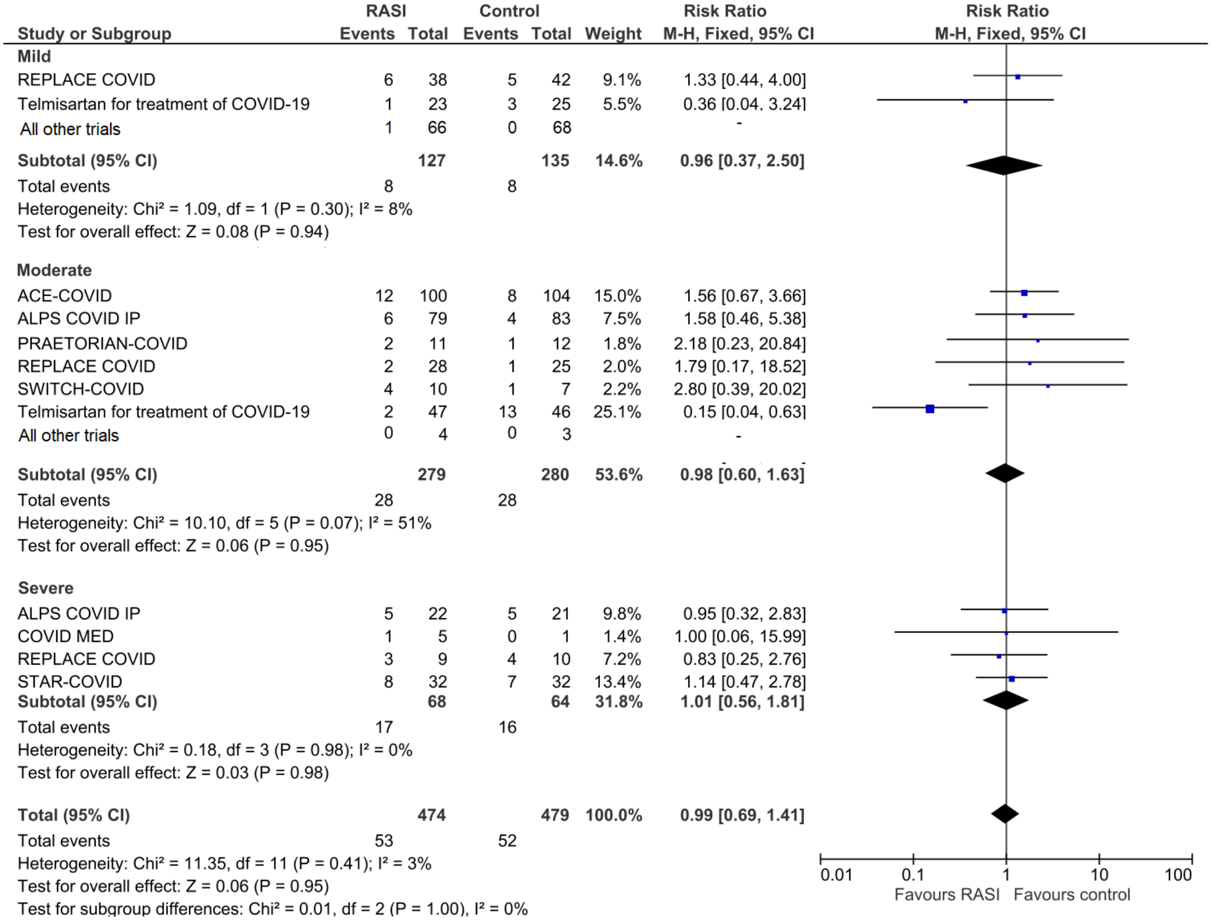

Figure S6: All-Cause Mortality by Angiotensin II type 1 Receptor Blocker

(A) Random Effects

Study or Subgroup	RASI Events	RASI Total	Control Events	Control Total	Weight	Risk Ratio M-H, Random, 95% CI
**Losartan**						
ALPS COVID IP	11	101	9	104	25.8%	1.26 [0.54, 2.91]
ARB use to minimize respiratory failure	1	16	1	15	5.6%	0.94 [0.06, 13.68]
COVID MED	2	9	0	3	5.2%	2.00 [0.12, 33.10]
Evaluation of the effects of losartan in COVID-19	2	41	5	39	12.9%	0.38 [0.08, 1.85]
ALPS-COVID OP	0	58	0	59		-
**Subtotal (95% CI)**		**225**		**220**	**49.5%**	**1.01 [0.51, 2.02]**
Total events	16		15			
Heterogeneity: Tau² = 0.00; Chi² = 1.97, df = 3 (P = 0.58); I² = 0%						
Test for overall effect: Z = 0.03 (P = 0.98)						
**Telmisartan**						
COVERAGE France	0	36	0	33		-
STAR-COVID	8	32	7	32	24.6%	1.14 [0.47, 2.78]
Telmisartan for treatment of COVID-19	3	78	16	80	18.4%	0.19 [0.06, 0.63]
**Subtotal (95% CI)**		**146**		**145**	**43.0%**	**0.49 [0.08, 2.97]**
Total events	11		23			
Heterogeneity: Tau² = 1.41; Chi² = 5.89, df = 1 (P = 0.02); I² = 83%						
Test for overall effect: Z = 0.78 (P = 0.44)						
**Valsartan**						
PRAETORIAN-COVID	2	11	1	12	7.5%	2.18 [0.23, 20.84]
**Subtotal (95% CI)**		**11**		**12**	**7.5%**	**2.18 [0.23, 20.84]**
Total events	2		1			
Heterogeneity: Not applicable						
Test for overall effect: Z = 0.68 (P = 0.50)						
**Total (95% CI)**		**382**		**377**	**100.0%**	**0.78 [0.40, 1.54]**
Total events	29		39			
Heterogeneity: Tau² = 0.28; Chi² = 9.52, df = 6 (P = 0.15); I² = 37%						
Test for overall effect: Z = 0.71 (P = 0.48)						
Test for subgroup differences: Chi² = 1.06, df = 2 (P = 0.59), I² = 0%						

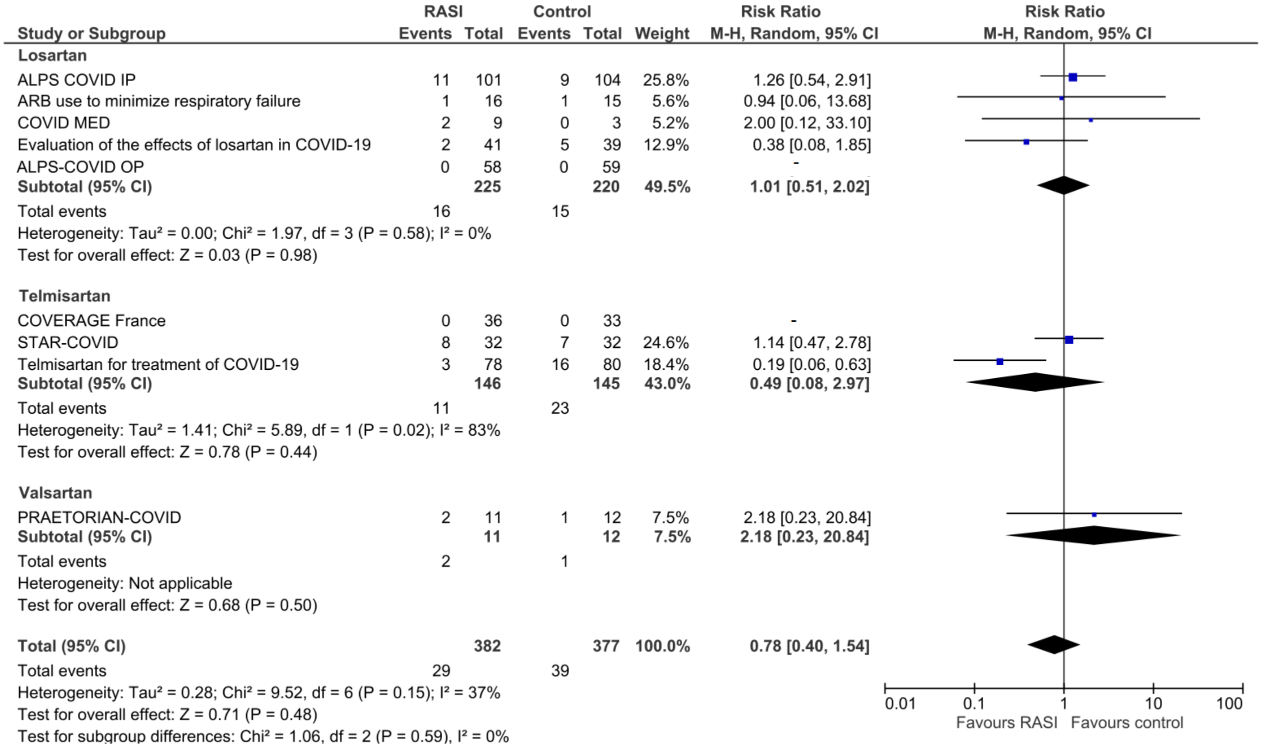

(B) Fixed Effects

Study or Subgroup	RASI Events	RASI Total	Control Events	Control Total	Weight	Risk Ratio M-H, Fixed, 95% CI
**Losartan**						
ALPS COVID IP	11	101	9	104	22.5%	1.26 [0.54, 2.91]
ARB use to minimize respiratory failure	1	16	1	15	2.6%	0.94 [0.06, 13.68]
COVID MED	2	9	0	3	1.8%	2.00 [0.12, 33.10]
Evaluation of the effects of losartan in COVID-19	2	41	5	39	13.0%	0.38 [0.08, 1.85]
ALPS-COVID OP	0	58	0	59		-
**Subtotal (95% CI)**		**225**		**220**	**39.9%**	**0.99 [0.50, 1.93]**
Total events	16		15			
Heterogeneity: Chi² = 1.97, df = 3 (P = 0.58); I² = 0%						
Test for overall effect: Z = 0.04 (P = 0.97)						
**Telmisartan**						
STAR-COVID	8	32	7	32	17.7%	1.14 [0.47, 2.78]
Telmisartan for treatment of COVID-19	3	78	16	80	40.0%	0.19 [0.06, 0.63]
COVERAGE France	0	36	0	33		-
**Subtotal (95% CI)**		**146**		**145**	**57.7%**	**0.48 [0.25, 0.94]**
Total events	11		23			
Heterogeneity: Chi² = 5.89, df = 1 (P = 0.02); I² = 83%						
Test for overall effect: Z = 2.13 (P = 0.03)						
**Valsartan**						
PRAETORIAN-COVID	2	11	1	12	2.4%	2.18 [0.23, 20.84]
**Subtotal (95% CI)**		**11**		**12**	**2.4%**	**2.18 [0.23, 20.84]**
Total events	2		1			
Heterogeneity: Not applicable						
Test for overall effect: Z = 0.68 (P = 0.50)						
**Total (95% CI)**		**382**		**377**	**100.0%**	**0.72 [0.46, 1.14]**
Total events	29		39			
Heterogeneity: Chi² = 9.52, df = 6 (P = 0.15); I² = 37%						
Test for overall effect: Z = 1.39 (P = 0.16)						
Test for subgroup differences: Chi² = 3.12, df = 2 (P = 0.21), I² = 55.9%						

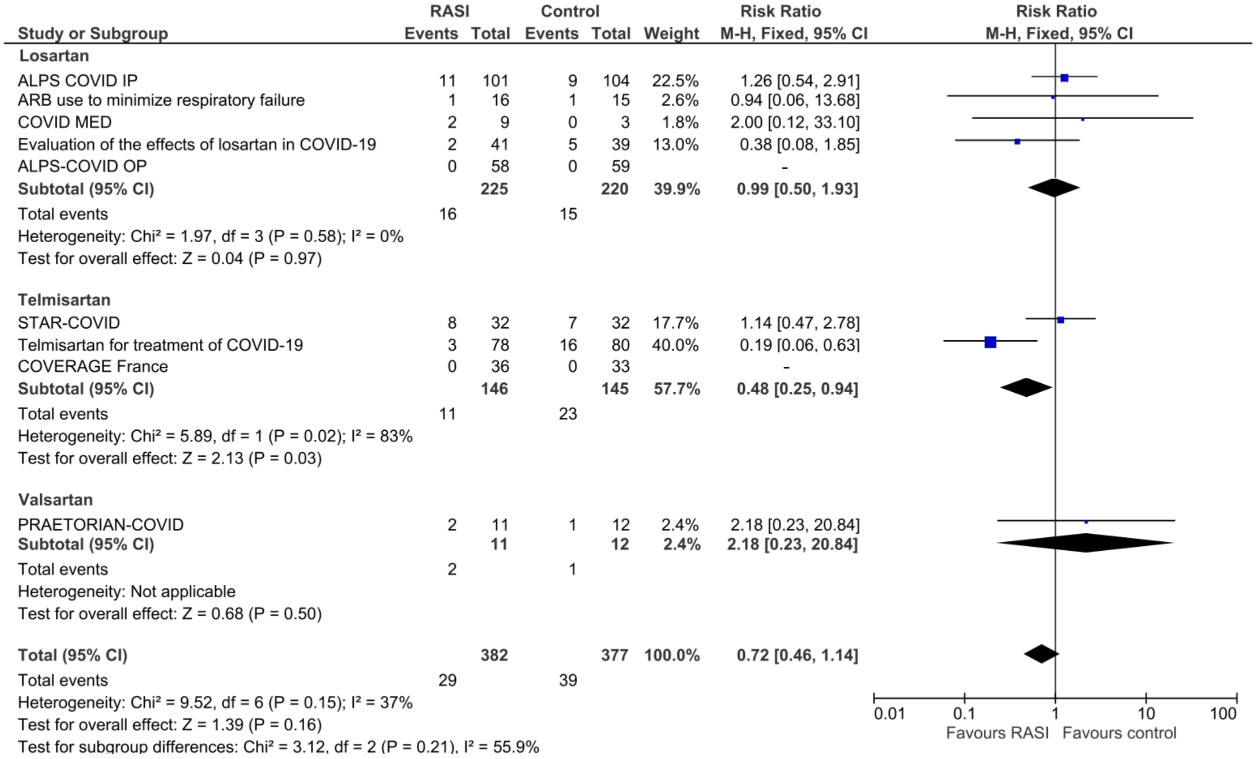

Figure S7: Publication bias and all-cause mortality

(A) Observed trials; (B) Observed and imputed trials.

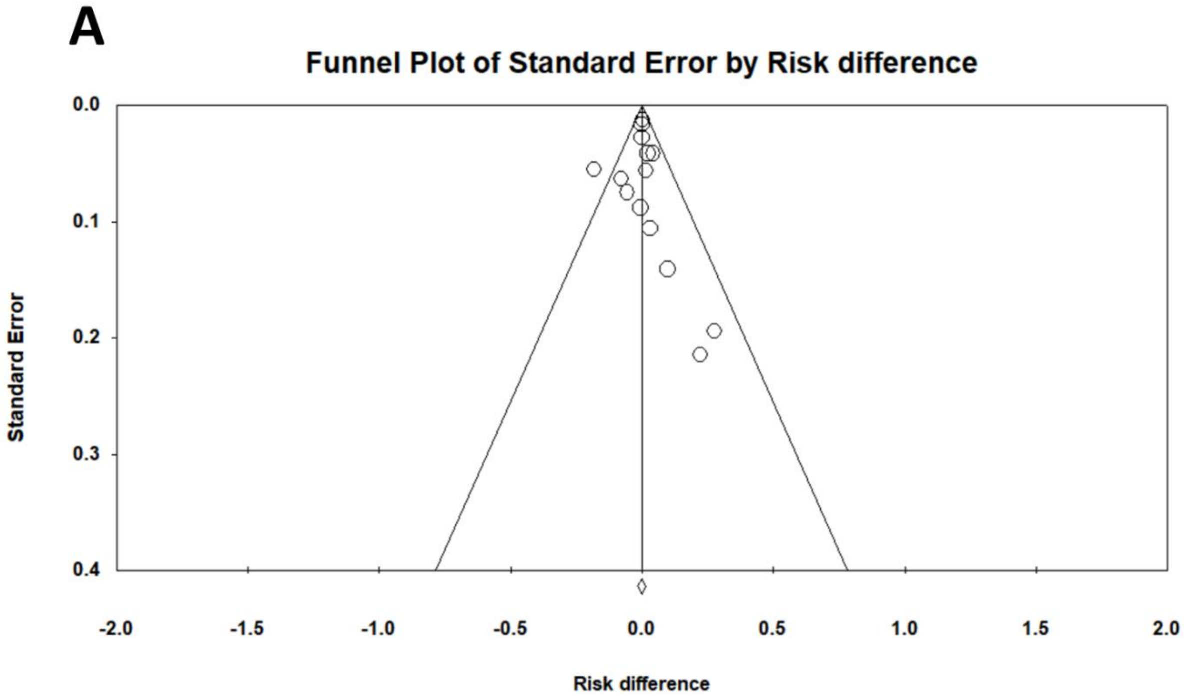

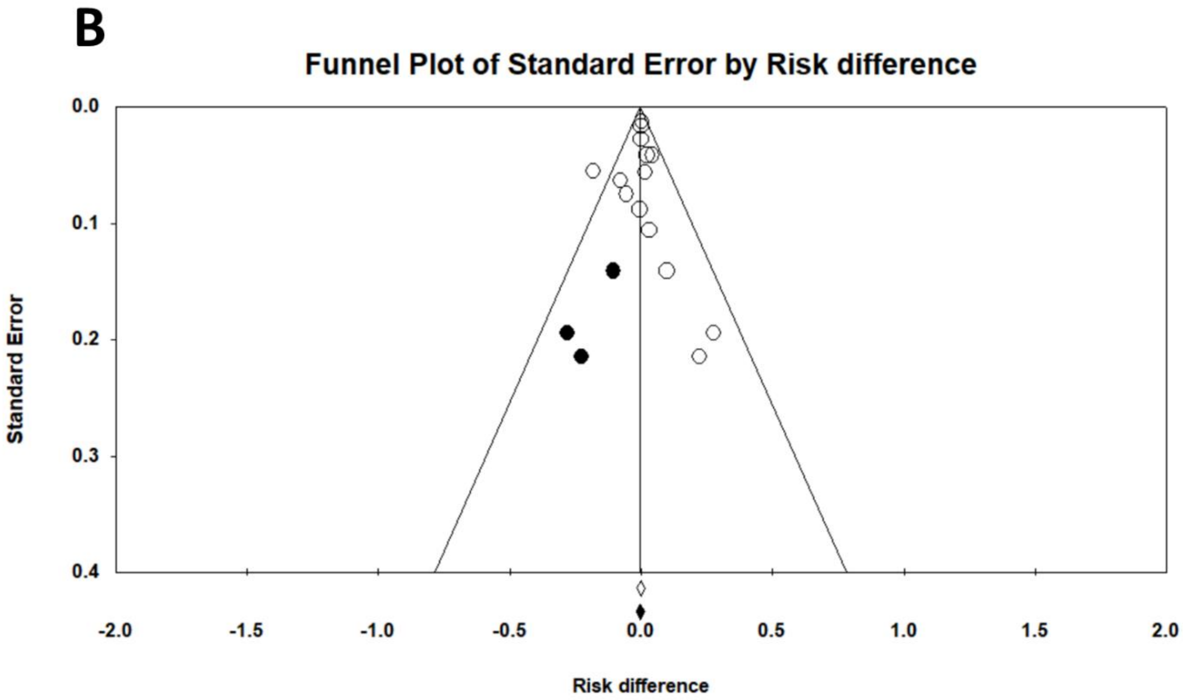

Open circles: observed trials; closed black circles: imputed trials

Figure S8: All-Cause Mortality – Age subgroups

Study or Subgroup	RASI Events	RASI Total	Control Events	Control Total	Weight	Risk Ratio M-H, Fixed, 95% CI
**<60 years**						
ALPS COVID IP	2	62	2	59	4.0%	0.95 [0.14, 6.54]
COVID MED	1	3	0	1	1.3%	1.50 [0.10, 22.62]
REPLACE COVID	4	31	1	33	1.9%	4.26 [0.50, 36.04]
STAR-COVID	6	25	6	27	11.1%	1.08 [0.40, 2.91]
SWITCH-COVID	3	6	1	3	2.6%	1.50 [0.25, 8.98]
All other trials	0	100	0	103		-
**Subtotal (95% CI)**		**227**		**226**	**20.8%**	**1.42 [0.70, 2.87]**
Total events	16		10			
Heterogeneity: Chi² = 1.48, df = 4 (P = 0.83); I² = 0%						
Test for overall effect: Z = 0.97 (P = 0.33)						
**≥60 years**						
ACE-COVID	12	89	8	83	16.0%	1.40 [0.60, 3.25]
ALPS COVID IP	9	39	7	45	12.5%	1.48 [0.61, 3.61]
COVID MED	1	6	0	2	1.3%	1.29 [0.07, 23.39]
PRAETORIAN-COVID	2	3	1	5	1.4%	3.33 [0.49, 22.90]
REPLACE COVID	7	44	9	44	17.4%	0.78 [0.32, 1.90]
STAR-COVID	2	7	1	5	2.2%	1.43 [0.17, 11.76]
SWITCH-COVID	1	4	0	5	0.9%	3.60 [0.18, 70.34]
Telmisartan for treatment of COVID-19	3	44	16	55	27.4%	0.23 [0.07, 0.75]
All other trials	0	39	0	33		-
**Subtotal (95% CI)**		**275**		**277**	**79.2%**	**0.93 [0.62, 1.40]**
Total events	37		42			
Heterogeneity: Chi² = 10.15, df = 7 (P = 0.18); I² = 31%						
Test for overall effect: Z = 0.34 (P = 0.73)						
**Total (95% CI)**		**502**		**503**	**100.0%**	**1.03 [0.73, 1.47]**
Total events	53		52			
Heterogeneity: Chi² = 11.87, df = 12 (P = 0.46); I² = 0%						
Test for overall effect: Z = 0.18 (P = 0.86)						
Test for subgroup differences: Chi² = 1.03, df = 1 (P = 0.31), I² = 2.7%						

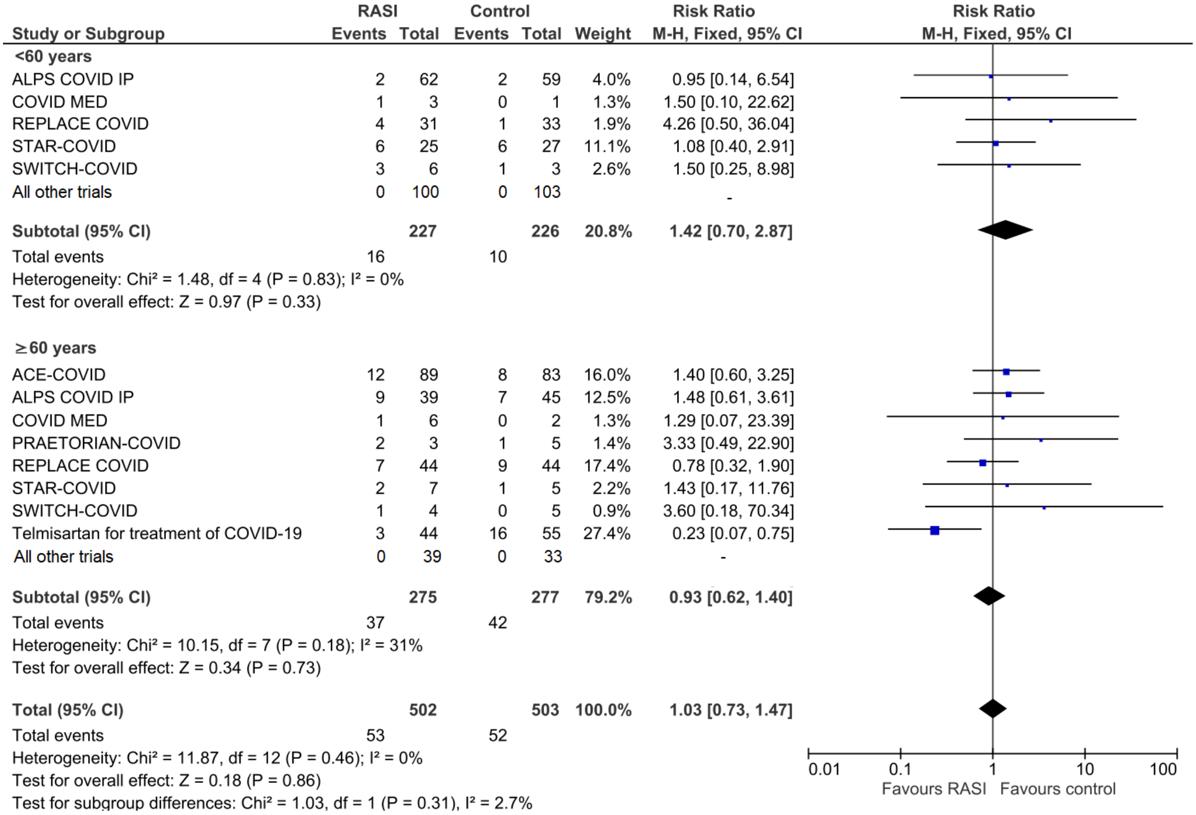

Figure S9: All-Cause Mortality – Sex

Study or Subgroup	RASI Events	RASI Total	Control Events	Control Total	Weight	Risk Ratio M-H, Fixed, 95% CI
**Male**						
ACE-COVID	11	64	5	65	9.5%	2.23 [0.82, 6.07]
ALPS COVID IP	6	60	8	63	14.9%	0.79 [0.29, 2.14]
COVID MED	1	6	0	2	1.3%	1.29 [0.07, 23.39]
PRAETORIAN-COVID	1	5	1	12	1.1%	2.40 [0.18, 31.29]
REPLACE COVID	7	42	7	42	13.3%	1.00 [0.38, 2.60]
STAR-COVID	6	19	5	21	9.1%	1.33 [0.48, 3.65]
SWITCH-COVID	2	7	0	4	1.2%	3.13 [0.19, 52.60]
Telmisartan for treatment of COVID-19	2	43	6	29	13.7%	0.22 [0.05, 1.04]
All other trials	0	39	0	48		-
**Subtotal (95% CI)**		**285**		**286**	**64.0%**	**1.08 [0.70, 1.66]**
Total events	36		32			
Heterogeneity: Chi² = 7.57, df = 7 (P = 0.37); I² = 8%						
Test for overall effect: Z = 0.36 (P = 0.72)						
**Female**						
ACE-COVID	1	36	3	39	5.5%	0.36 [0.04, 3.32]
ALPS COVID IP	5	41	1	41	1.9%	5.00 [0.61, 40.95]
COVID MED	1	3	0	1	1.3%	1.50 [0.10, 22.62]
PRAETORIAN-COVID	1	6	0	4	1.1%	2.14 [0.11, 42.52]
REPLACE COVID	4	33	3	35	5.5%	1.41 [0.34, 5.85]
STAR-COVID	2	13	2	11	4.1%	0.85 [0.14, 5.06]
SWITCH-COVID	2	3	1	4	1.6%	2.67 [0.41, 17.42]
Telmisartan for treatment of COVID-19	1	27	10	42	14.9%	0.16 [0.02, 1.15]
All other trials	0	55	0	44		-
**Subtotal (95% CI)**		**217**		**221**	**36.0%**	**0.94 [0.50, 1.75]**
Total events	17		20			
Heterogeneity: Chi² = 8.18, df = 7 (P = 0.32); I² = 14%						
Test for overall effect: Z = 0.20 (P = 0.84)						
**Total (95% CI)**		**502**		**507**	**100.0%**	**1.03 [0.72, 1.47]**
Total events	53		52			
Heterogeneity: Chi² = 15.66, df = 15 (P = 0.41); I² = 4%						
Test for overall effect: Z = 0.17 (P = 0.86)						
Test for subgroup differences: Chi² = 0.14, df = 1 (P = 0.71), I² = 0%						

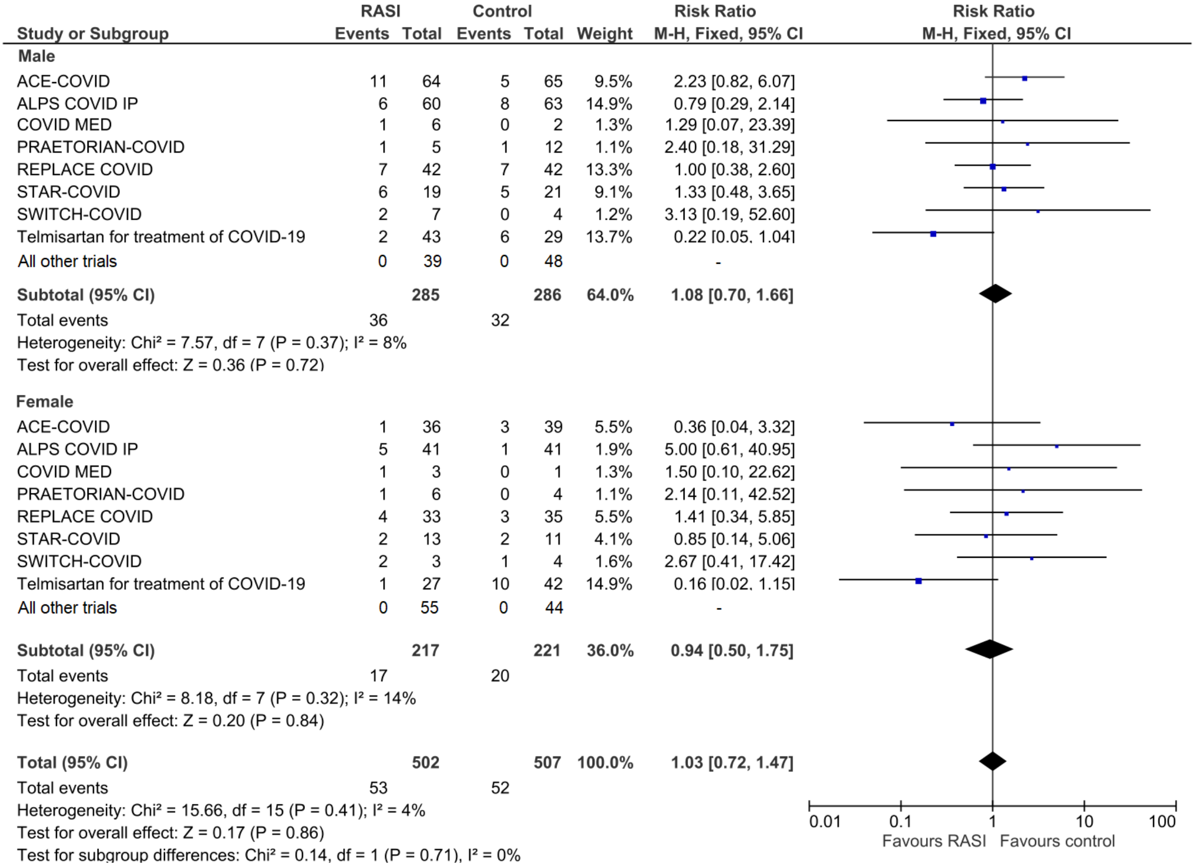

Figure S10: All-Cause Mortality – Ethnicity

Study or Subgroup	RASI Events	RASI Total	Control Events	Control Total	Weight	Risk Ratio M-H, Fixed, 95% CI
**White**						
ACE-COVID	12	100	8	104	21.8%	1.56 [0.67, 3.66]
ALPS COVID IP	4	35	7	47	16.6%	0.77 [0.24, 2.42]
COVID MED	2	9	0	3	2.0%	2.00 [0.12, 33.10]
PRAETORIAN-COVID	2	10	1	12	2.5%	2.40 [0.25, 22.75]
REPLACE COVID	2	30	1	31	2.7%	2.07 [0.20, 21.61]
SWITCH-COVID	3	7	0	6	1.5%	6.13 [0.38, 99.14]
All other trials	0	45	0	39		-
**Subtotal (95% CI)**		**236**		**242**	**47.1%**	**1.52 [0.85, 2.72]**
Total events	25		17			
Heterogeneity: Chi² = 2.59, df = 5 (P = 0.76); I² = 0%						
Test for overall effect: Z = 1.40 (P = 0.16)						
**South-East/East Asian**						
REPLACE COVID	0	1	1	3	2.8%	0.67 [0.04, 10.05]
All other trials	0	7	0	2		-
**Subtotal (95% CI)**		**8**		**5**	**2.8%**	**0.67 [0.04, 10.05]**
Total events	0		1			
Heterogeneity: Not applicable						
Test for overall effect: Z = 0.29 (P = 0.77)						
**African**						
REPLACE COVID	1	10	0	13	1.2%	3.82 [0.17, 84.90]
SWITCH-COVID	1	3	1	2	3.3%	0.67 [0.08, 5.54]
All other trials	0	5	0	4		-
**Subtotal (95% CI)**		**18**		**19**	**4.6%**	**1.51 [0.28, 8.07]**
Total events	2		1			
Heterogeneity: Chi² = 0.92, df = 1 (P = 0.34); I² = 0%						
Test for overall effect: Z = 0.48 (P = 0.63)						
**Other**						
ALPS COVID IP	2	22	1	25	2.6%	2.27 [0.22, 23.38]
REPLACE COVID	8	34	8	30	23.6%	0.88 [0.38, 2.06]
STAR-COVID	8	32	7	32	19.4%	1.14 [0.47, 2.78]
All other trials	0	8	0	11		-
**Subtotal (95% CI)**		**96**		**98**	**45.6%**	**1.07 [0.59, 1.93]**
Total events	18		16			
Heterogeneity: Chi² = 0.62, df = 2 (P = 0.73); I² = 0%						
Test for overall effect: Z = 0.23 (P = 0.82)						
**Total (95% CI)**		**358**		**364**	**100.0%**	**1.29 [0.87, 1.92]**
Total events	45		35			
Heterogeneity: Chi² = 4.86, df = 11 (P = 0.94); I² = 0%						
Test for overall effect: Z = 1.26 (P = 0.21)						
Test for subgroup differences: Chi² = 0.93, df = 3 (P = 0.82), I² = 0%						

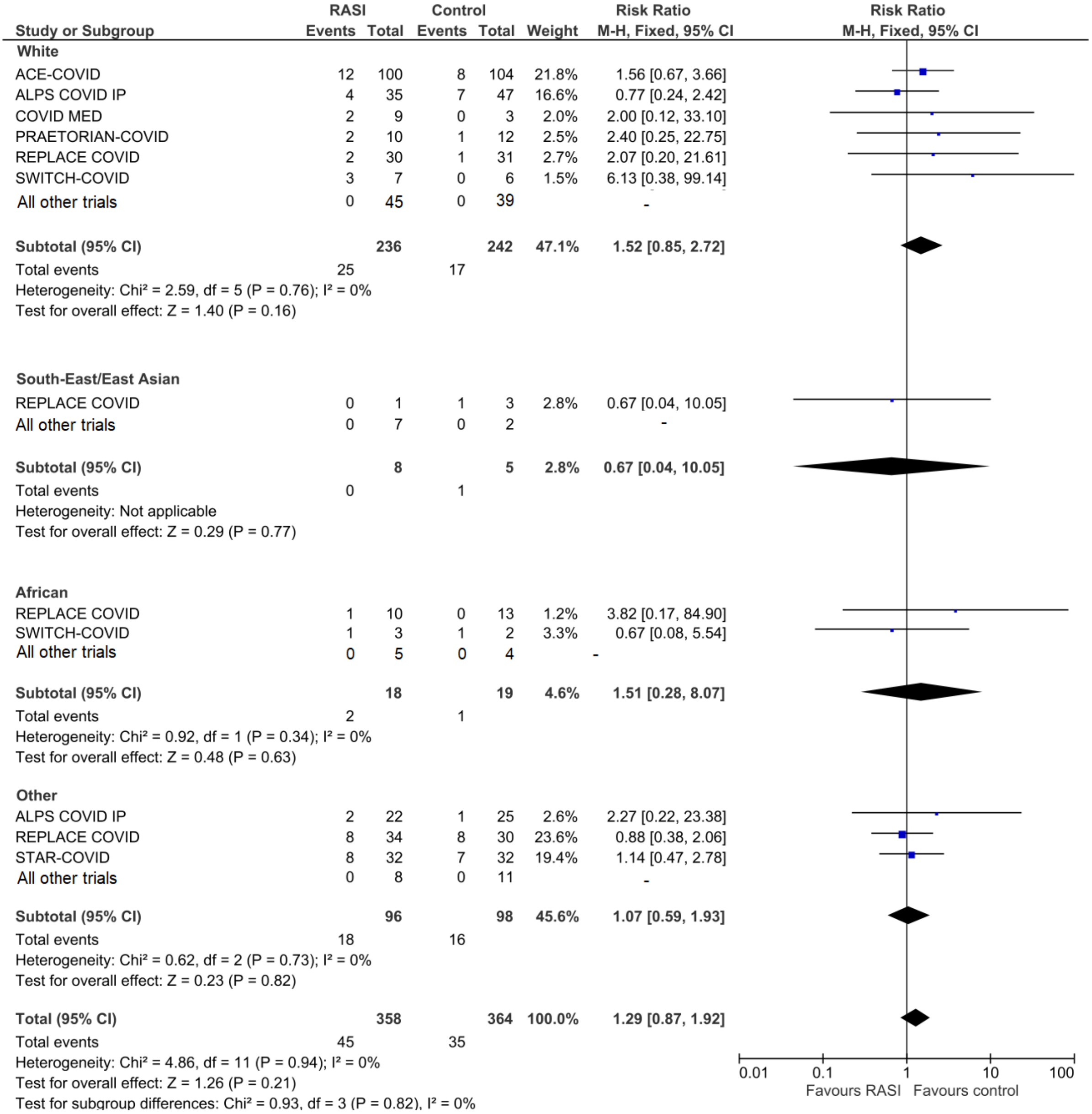

Figure S11: All-Cause Mortality – COPD vs no COPD

(A) Random Effects

Study or Subgroup	RASI Events	RASI Total	Control Events	Control Total	Weight	Risk Ratio M-H, Random, 95% CI
**COPD**						
ACE-COVID	5	22	0	10	4.3%	5.26 [0.32, 86.90]
ALPS COVID IP	8	10	3	65	10.1%	17.33 [5.50, 54.62]
REPLACE COVID	3	9	3	17	9.0%	1.89 [0.47, 7.52]
SWITCH-COVID	1	1	1	1	10.2%	1.00 [0.32, 3.10]
Telmisartan for treatment of COVID-19	0	6	1	8	3.9%	0.43 [0.02, 9.00]
All other trials	0	8	0	3		-
**Subtotal (95% CI)**		**56**		**104**	**37.5%**	**2.68 [0.65, 11.02]**
Total events	17		8			
Heterogeneity: Tau² = 1.73; Chi² = 14.97, df = 4 (P = 0.005); I² = 73%						
Test for overall effect: Z = 1.37 (P = 0.17)						
**No COPD**						
ACE-COVID	7	78	8	94	10.9%	1.05 [0.40, 2.78]
ALPS COVID IP	3	89	6	89	9.1%	0.50 [0.13, 1.94]
COVID MED	1	2	0	2	4.4%	3.00 [0.19, 47.96]
PRAETORIAN-COVID	2	11	1	11	5.7%	2.00 [0.21, 18.98]
REPLACE COVID	8	66	7	80	11.0%	1.39 [0.53, 3.62]
STAR-COVID	8	32	7	32	11.3%	1.14 [0.47, 2.78]
Telmisartan for treatment of COVID-19	3	64	15	63	9.9%	0.20 [0.06, 0.65]
All other trials	0	101	0	98		-
**Subtotal (95% CI)**		**443**		**469**	**62.5%**	**0.86 [0.47, 1.56]**
Total events	32		44			
Heterogeneity: Tau² = 0.23; Chi² = 9.57, df = 6 (P = 0.14); I² = 37%						
Test for overall effect: Z = 0.50 (P = 0.62)						
**Total (95% CI)**		**499**		**573**	**100.0%**	**1.36 [0.67, 2.75]**
Total events	49		52			
Heterogeneity: Tau² = 0.94; Chi² = 34.28, df = 11 (P = 0.0003); I² = 68%						
Test for overall effect: Z = 0.85 (P = 0.40)						
Test for subgroup differences: Chi² = 2.12, df = 1 (P = 0.15), I² = 52.8%						

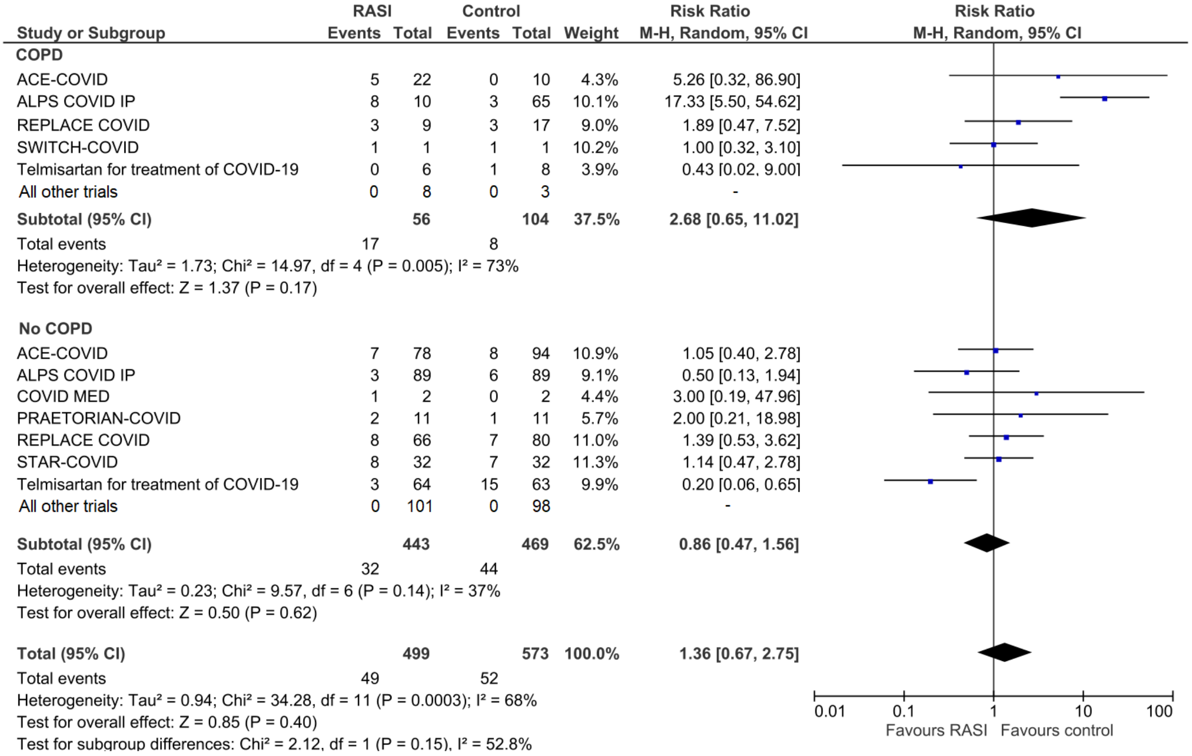

(B) Fixed Effects

Study or Subgroup	RASI Events	RASI Total	Control Events	Control Total	Weight	Risk Ratio M-H, Fixed, 95% CI
**COPD**						
ACE-COVID	5	22	0	10	1.4%	5.26 [0.32, 86.90]
ALPS COVID IP	8	10	3	65	1.6%	17.33 [5.50, 54.62]
REPLACE COVID	3	9	3	17	4.2%	1.89 [0.47, 7.52]
SWITCH-COVID	1	1	1	1	3.0%	1.00 [0.32, 3.10]
Telmisartan for treatment of COVID-19	0	6	1	8	2.6%	0.43 [0.02, 9.00]
All other trials	0	8	0	3		-
**Subtotal (95% CI)**		**56**		**104**	**12.8%**	**3.68 [1.96, 6.89]**
Total events	17		8			
Heterogeneity: Chi² = 14.97, df = 4 (P = 0.005); I² = 73%						
Test for overall effect: Z = 4.06 (P < 0.0001)						
**No COPD**						
ACE-COVID	7	78	8	94	14.6%	1.05 [0.40, 2.78]
ALPS COVID IP	3	89	6	89	12.1%	0.50 [0.13, 1.94]
COVID MED	1	2	0	2	1.0%	3.00 [0.19, 47.96]
PRAETORIAN-COVID	2	11	1	11	2.0%	2.00 [0.21, 18.98]
REPLACE COVID	8	66	7	80	12.8%	1.39 [0.53, 3.62]
STAR-COVID	8	32	7	32	14.1%	1.14 [0.47, 2.78]
Telmisartan for treatment of COVID-19	3	64	15	63	30.5%	0.20 [0.06, 0.65]
All other trials	0	101	0	98		-
**Subtotal (95% CI)**		**443**		**469**	**87.2%**	**0.78 [0.51, 1.20]**
Total events	32		44			
Heterogeneity: Chi² = 9.57, df = 6 (P = 0.14); I² = 37%						
Test for overall effect: Z = 1.13 (P = 0.26)						
**Total (95% CI)**		**499**		**573**	**100.0%**	**1.16 [0.83, 1.62]**
Total events	49		52			
Heterogeneity: Chi² = 34.28, df = 11 (P = 0.0003); I² = 68%						
Test for overall effect: Z = 0.85 (P = 0.40)						
Test for subgroup differences: Chi² = 16.02, df = 1 (P < 0.0001), I² = 93.8%						

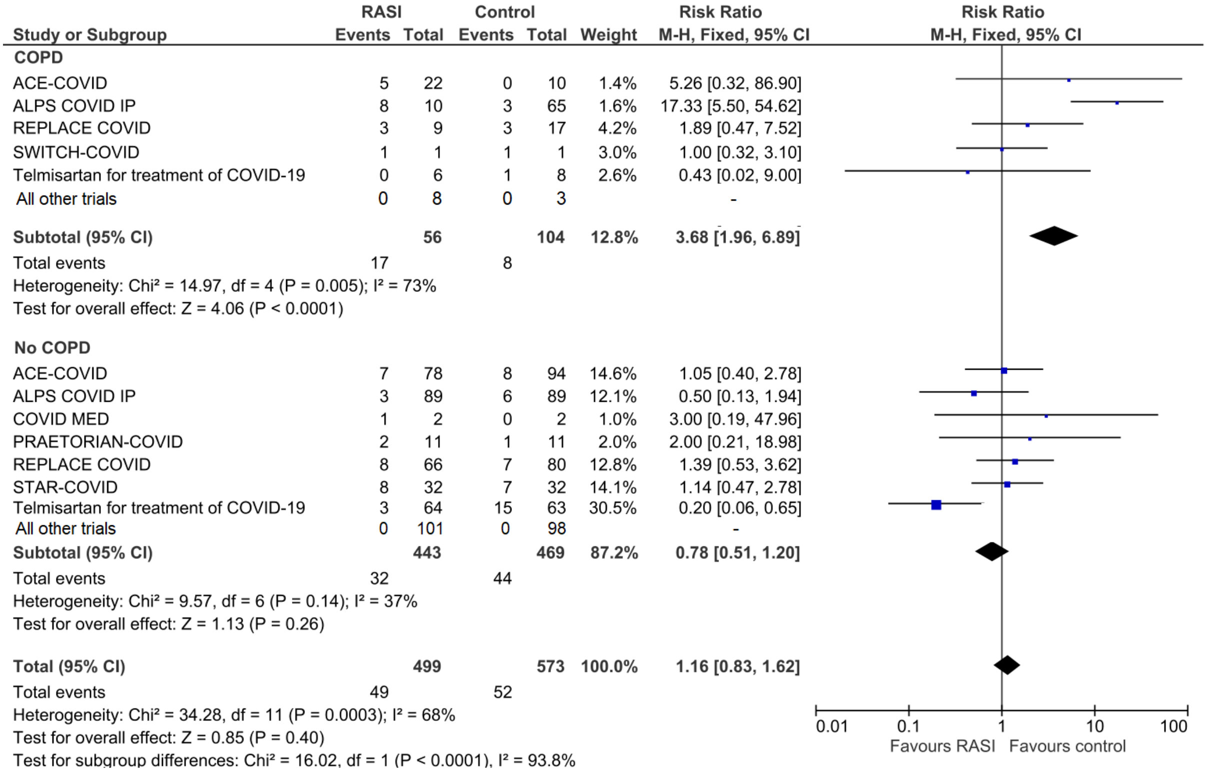

Figure S12: All-Cause Mortality – Hypertension vs no Hypertension

Study or Subgroup	RASI Events	RASI Total	Control Events	Control Total	Weight	Risk Ratio M-H, Fixed, 95% CI
**Hypertension**						
ACE-COVID	12	99	8	100	15.5%	1.52 [0.65, 3.55]
ALPS COVID IP	6	35	6	46	10.1%	1.31 [0.46, 3.73]
PRAETORIAN-COVID	0	2	1	6	1.8%	0.78 [0.04, 14.15]
REPLACE COVID	11	75	10	77	19.3%	1.13 [0.51, 2.50]
SWITCH-COVID	4	10	1	8	2.2%	3.20 [0.44, 23.28]
Telmisartan for treatment of COVID-19	1	30	11	35	19.8%	0.11 [0.01, 0.77]
All other trials	0	17	0	7		-
**Subtotal (95% CI)**		**268**		**279**	**68.7%**	**1.00 [0.65, 1.56]**
Total events	34		37			
Heterogeneity: Chi² = 7.49, df = 5 (P = 0.19); I² = 33%						
Test for overall effect: Z = 0.02 (P = 0.98)						
**No Hypertension**						
ALPS COVID IP	5	65	3	58	6.2%	1.49 [0.37, 5.95]
PRAETORIAN-COVID	2	9	0	6	1.1%	3.50 [0.20, 62.27]
STAR-COVID	8	29	7	29	13.7%	1.14 [0.48, 2.74]
Telmisartan for treatment of COVID-19	2	40	5	36	10.3%	0.36 [0.07, 1.74]
All other trials	0	9	0	15		-
**Subtotal (95% CI)**		**204**		**200**	**31.3%**	**1.04 [0.55, 1.96]**
Total events	17		15			
Heterogeneity: Chi² = 2.72, df = 3 (P = 0.44); I² = 0%						
Test for overall effect: Z = 0.12 (P = 0.90)						
**Total (95% CI)**		**472**		**479**	**100.0%**	**1.02 [0.71, 1.46]**
Total events	51		52			
Heterogeneity: Chi² = 10.16, df = 9 (P = 0.34); I² = 11%						
Test for overall effect: Z = 0.09 (P = 0.93)						
Test for subgroup differences: Chi² = 0.01, df = 1 (P = 0.93), I² = 0%						

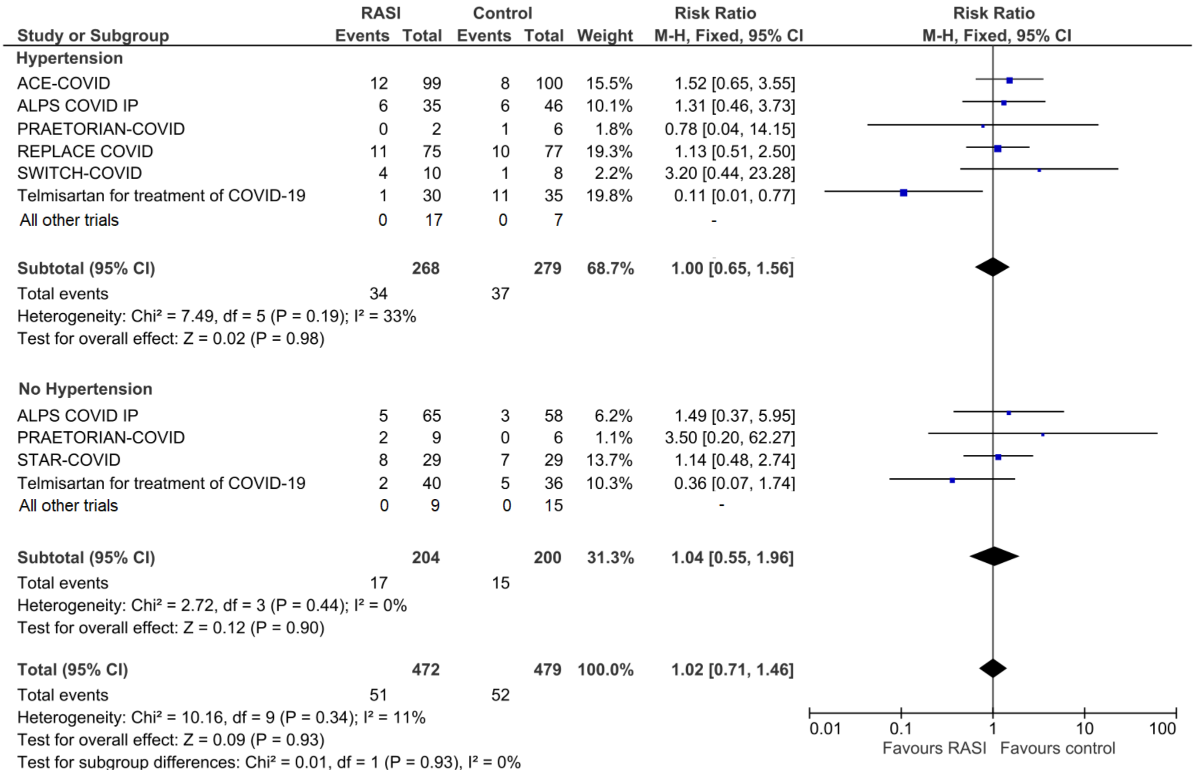

Figure S13: All-Cause Mortality – Diabetes Mellitus vs no Diabetes

Study or Subgroup	RASI Events	RASI Total	Control Events	Control Total	Weight	Risk Ratio M-H, Fixed, 95% CI
**Diabetes Mellitus**						
ACE-COVID	6	37	1	30	2.1%	4.86 [0.62, 38.23]
ALPS COVID IP	2	19	2	25	3.2%	1.32 [0.20, 8.51]
PRAETORIAN-COVID	1	1	1	3	1.9%	2.00 [0.45, 8.94]
REPLACE COVID	5	42	6	37	11.9%	0.73 [0.24, 2.21]
STAR-COVID	2	9	2	7	4.2%	0.78 [0.14, 4.23]
SWITCH-COVID	3	7	0	3	1.2%	3.50 [0.23, 52.56]
Telmisartan for treatment of COVID-19	0	12	4	14	7.8%	0.13 [0.01, 2.16]
All other trials	0	8	0	6		-
**Subtotal (95% CI)**		**135**		**125**	**32.4%**	**1.09 [0.60, 2.01]**
Total events	19		16			
Heterogeneity: Chi² = 6.25, df = 6 (P = 0.40); I² = 4%						
Test for overall effect: Z = 0.29 (P = 0.77)						
**No Diabetes**						
ACE-COVID	6	63	7	74	12.1%	1.01 [0.36, 2.84]
ALPS COVID IP	9	80	7	77	13.4%	1.24 [0.48, 3.16]
COVID MED	2	7	0	2	1.4%	1.88 [0.12, 28.78]
PRAETORIAN-COVID	1	10	0	9	1.0%	2.73 [0.12, 59.57]
REPLACE COVID	6	33	4	40	6.8%	1.82 [0.56, 5.91]
STAR-COVID	6	23	5	25	9.0%	1.30 [0.46, 3.70]
SWITCH-COVID	1	3	1	5	1.4%	1.67 [0.16, 17.89]
Telmisartan for treatment of COVID-19	3	58	12	57	22.7%	0.25 [0.07, 0.82]
All other trials	0	88	0	87		-
**Subtotal (95% CI)**		**365**		**376**	**67.6%**	**0.97 [0.63, 1.50]**
Total events	34		36			
Heterogeneity: Chi² = 7.45, df = 7 (P = 0.38); I² = 6%						
Test for overall effect: Z = 0.12 (P = 0.91)						
**Total (95% CI)**		**500**		**501**	**100.0%**	**1.01 [0.71, 1.44]**
Total events	53		52			
Heterogeneity: Chi² = 13.74, df = 14 (P = 0.47); I² = 0%						
Test for overall effect: Z = 0.07 (P = 0.94)						
Test for subgroup differences: Chi² = 0.09, df = 1 (P = 0.76), I² = 0%						

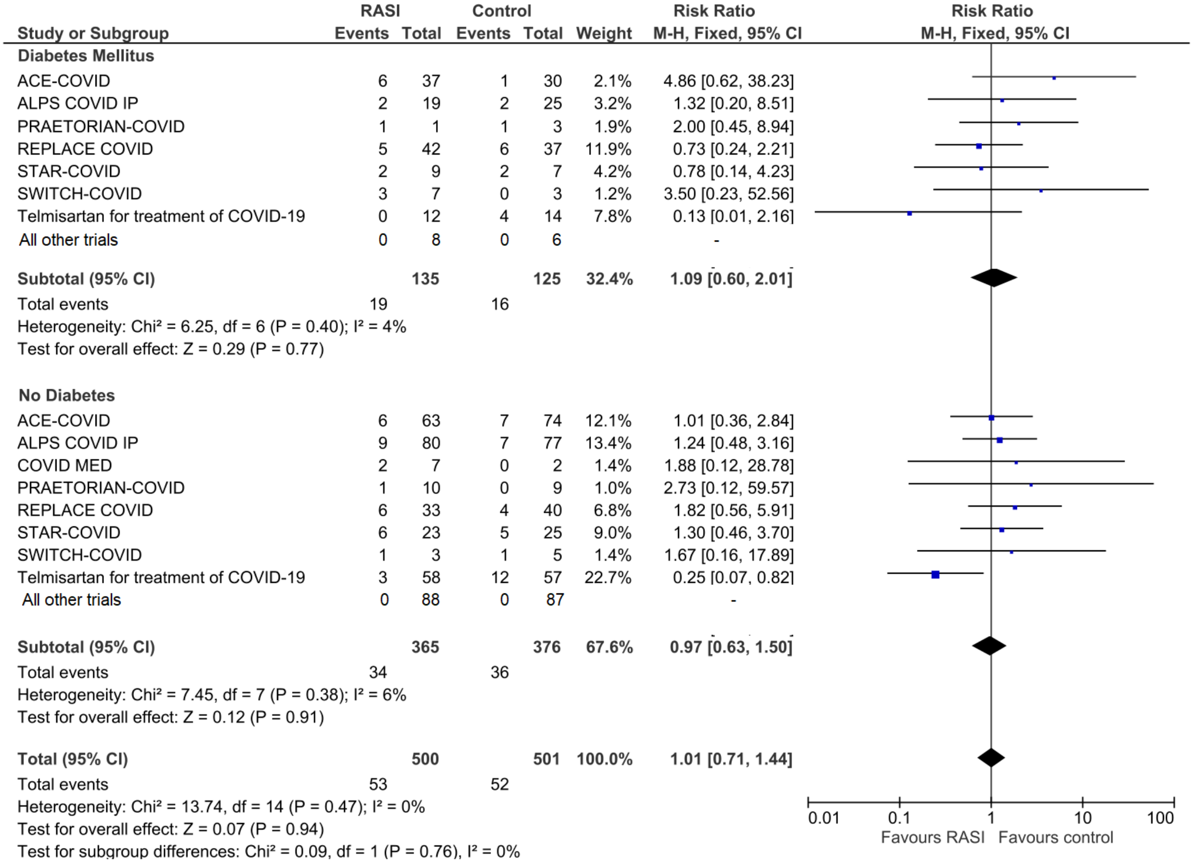

Figure S14: All-Cause Mortality – Obesity vs No Obesity

Study or Subgroup	RASI Events	RASI Total	Control Events	Control Total	Weight	Risk Ratio M-H, Fixed, 95% CI
**Obesity**						
ACE-COVID	1	22	4	36	6.2%	0.41 [0.05, 3.43]
ALPS COVID IP	8	65	3	65	6.2%	2.67 [0.74, 9.61]
COVID MED	1	3	0	2	1.2%	2.25 [0.13, 38.09]
REPLACE COVID	5	36	4	37	8.1%	1.28 [0.37, 4.40]
STAR-COVID	0	9	3	11	6.5%	0.17 [0.01, 2.94]
SWITCH-COVID	1	3	0	2	1.2%	2.25 [0.13, 38.09]
Telmisartan for treatment of COVID-19	0	14	2	8	6.4%	0.12 [0.01, 2.23]
All other trials	0	35	0	30		-
**Subtotal (95% CI)**		**187**		**191**	**35.8%**	**1.02 [0.54, 1.91]**
Total events	16		16			
Heterogeneity: Chi² = 7.18, df = 6 (P = 0.30); I² = 16%						
Test for overall effect: Z = 0.07 (P = 0.95)						
**No Obesity**						
ACE-COVID	11	77	4	68	8.7%	2.43 [0.81, 7.27]
ALPS COVID IP	3	38	6	38	12.3%	0.50 [0.13, 1.85]
COVID MED	1	6	0	1	1.6%	0.86 [0.05, 13.93]
PRAETORIAN-COVID	2	6	1	8	1.8%	2.67 [0.31, 23.00]
REPLACE COVID	3	23	1	25	2.0%	3.26 [0.36, 29.17]
STAR-COVID	8	23	4	21	8.6%	1.83 [0.64, 5.19]
SWITCH-COVID	3	7	1	6	2.2%	2.57 [0.35, 18.68]
Telmisartan for treatment of COVID-19	3	56	14	63	27.1%	0.24 [0.07, 0.80]
All other trials	0	64	0	66		-
**Subtotal (95% CI)**		**300**		**296**	**64.2%**	**1.05 [0.67, 1.67]**
Total events	34		31			
Heterogeneity: Chi² = 12.93, df = 7 (P = 0.07); I² = 46%						
Test for overall effect: Z = 0.23 (P = 0.82)						
**Total (95% CI)**		**487**		**487**	**100.0%**	**1.04 [0.72, 1.51]**
Total events	50		47			
Heterogeneity: Chi² = 20.10, df = 14 (P = 0.13); I² = 30%						
Test for overall effect: Z = 0.22 (P = 0.83)						
Test for subgroup differences: Chi² = 0.01, df = 1 (P = 0.94), I² = 0%						

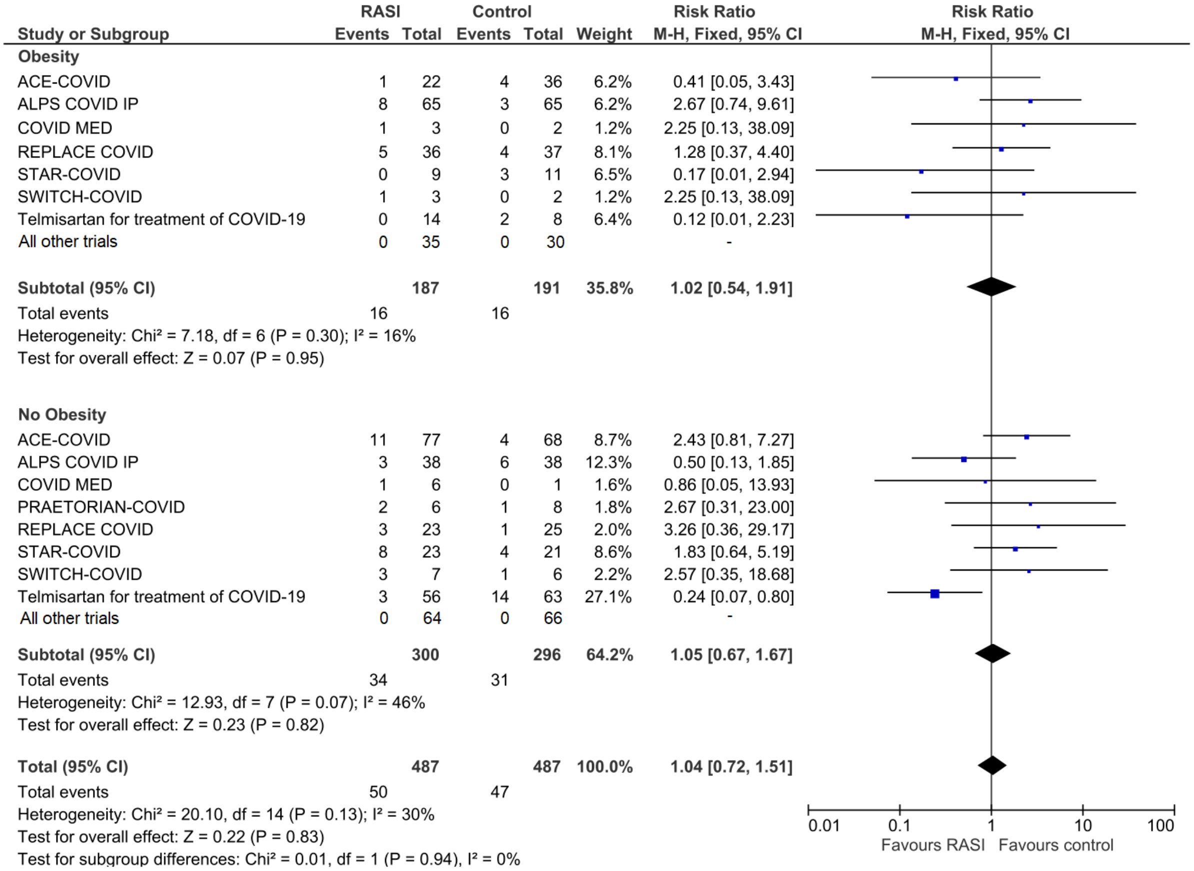

Figure S15: All-Cause Mortality – CVD vs no CVD

Study or Subgroup	RASI Events	RASI Total	Control Events	Control Total	Weight	Risk Ratio M-H, Fixed, 95% CI
**CVD**						
ACE-COVID	7	30	2	32	4.9%	3.73 [0.84, 16.57]
ALPS COVID IP	1	4	4	9	6.2%	0.56 [0.09, 3.57]
COVID MED	1	2	0	2	1.3%	3.00 [0.19, 47.96]
PRAETORIAN-COVID	1	3	0	5	1.0%	4.50 [0.24, 85.12]
REPLACE COVID	1	10	2	14	4.2%	0.70 [0.07, 6.70]
Telmisartan for treatment of COVID-19	2	6	4	8	8.7%	0.67 [0.18, 2.51]
**Subtotal (95% CI)**		**55**		**70**	**26.3%**	**1.48 [0.73, 2.98]**
Total events	13		12			
Heterogeneity: Chi² = 5.14, df = 5 (P = 0.40); I² = 3%						
Test for overall effect: Z = 1.09 (P = 0.27)						
**No CVD**						
ACE-COVID	5	70	6	72	11.5%	0.86 [0.27, 2.68]
ALPS COVID IP	10	97	5	95	9.8%	1.96 [0.70, 5.52]
COVID MED	1	7	0	1	1.6%	0.75 [0.05, 12.34]
PRAETORIAN-COVID	1	8	1	7	2.1%	0.88 [0.07, 11.54]
REPLACE COVID	10	65	8	63	15.8%	1.21 [0.51, 2.87]
STAR-COVID	8	32	7	32	13.6%	1.14 [0.47, 2.78]
SWITCH-COVID	4	10	1	8	2.2%	3.20 [0.44, 23.28]
Telmisartan for treatment of COVID-19	1	64	12	63	23.5%	0.08 [0.01, 0.61]
All other trials	0	58	0	59		-
**Subtotal (95% CI)**		**411**		**400**	**79.8%**	**0.94 [0.63, 1.43]**
Total events	40		40			
Heterogeneity: Chi² = 9.59, df = 7 (P = 0.21); I² = 27%						
Test for overall effect: Z = 0.27 (P = 0.79)						
**Total (95% CI)**		**466**		**470**	**100.0%**	**1.05 [0.74, 1.50]**
Total events	53		52			
Heterogeneity: Chi² = 14.40, df = 13 (P = 0.35); I² = 10%						
Test for overall effect: Z = 0.28 (P = 0.78)						
Test for subgroup differences: Chi² = 1.17, df = 1 (P = 0.28), I² = 14.3%						

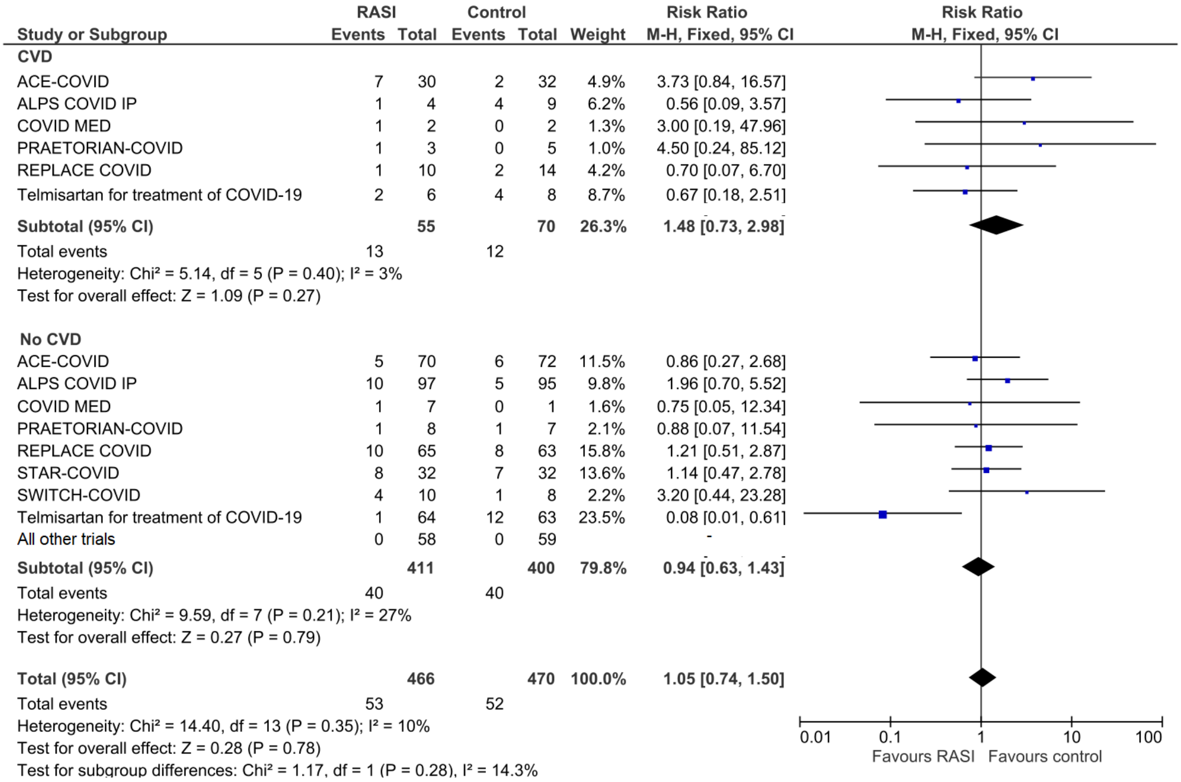

Figure S16: All-Cause Mortality – CKD vs no CKD

Study or Subgroup	RASI Events	RASI Total	Control Events	Control Total	Weight	Risk Ratio M-H, Fixed, 95% CI
**CKD**						
ACE-COVID	4	21	3	16	6.6%	1.02 [0.26, 3.91]
REPLACE COVID	1	12	1	17	1.6%	1.42 [0.10, 20.49]
All other trials	0	4	0	2		-
**Subtotal (95% CI)**		**37**		**35**	**8.2%**	**1.09 [0.33, 3.64]**
Total events	5		4			
Heterogeneity: Chi² = 0.05, df = 1 (P = 0.83); I² = 0%						
Test for overall effect: Z = 0.15 (P = 0.88)						
**No CKD**						
ACE-COVID	8	79	5	88	9.2%	1.78 [0.61, 5.22]
ALPS COVID IP	11	101	9	104	17.2%	1.26 [0.54, 2.91]
PRAETORIAN-COVID	2	11	1	11	1.9%	2.00 [0.21, 18.98]
REPLACE COVID	9	61	9	59	17.7%	0.97 [0.41, 2.27]
STAR-COVID	8	32	7	32	13.6%	1.14 [0.47, 2.78]
SWITCH-COVID	4	10	1	7	2.3%	2.80 [0.39, 20.02]
Telmisartan for treatment of COVID-19	3	66	16	71	29.9%	0.20 [0.06, 0.66]
All other trials	0	67	0	62		-
**Subtotal (95% CI)**		**427**		**434**	**91.8%**	**0.95 [0.65, 1.39]**
Total events	45		48			
Heterogeneity: Chi² = 10.06, df = 6 (P = 0.12); I² = 40%						
Test for overall effect: Z = 0.28 (P = 0.78)						
**Total (95% CI)**		**464**		**469**	**100.0%**	**0.96 [0.67, 1.38]**
Total events	50		52			
Heterogeneity: Chi² = 10.10, df = 8 (P = 0.26); I² = 21%						
Test for overall effect: Z = 0.22 (P = 0.82)						
Test for subgroup differences: Chi² = 0.05, df = 1 (P = 0.82), I² = 0%						

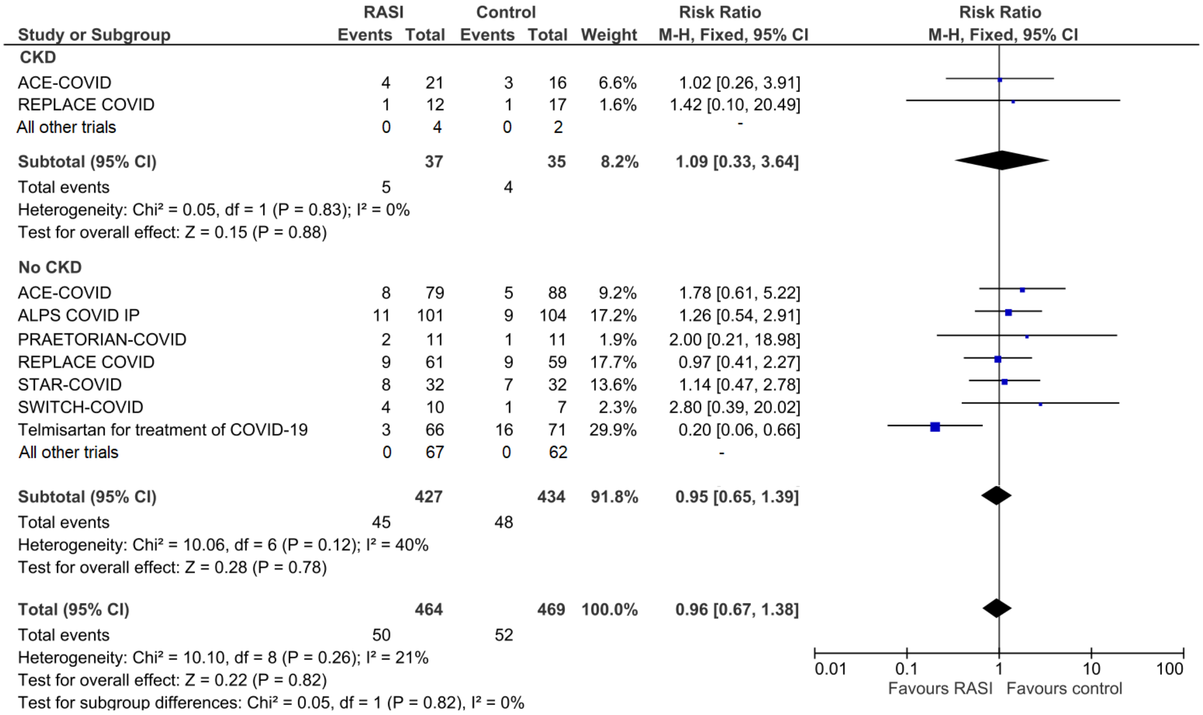

Figure S17: All-Cause Mortality - Smoker vs Non-Smoker

Study or Subgroup	RASI Events	RASI Total	Control Events	Control Total	Weight	Risk Ratio M-H, Fixed, 95% CI
**Ever smoked**						
PRAETORIAN-COVID	2	3	1	5	4.1%	3.33 [0.49, 22.90]
REPLACE COVID	2	23	2	29	9.6%	1.26 [0.19, 8.28]
STAR-COVID	2	6	2	8	9.3%	1.33 [0.26, 6.94]
All other trials	0	4	0	3		-
**Subtotal (95% CI)**		**36**		**45**	**22.9%**	**1.66 [0.59, 4.65]**
Total events	6		5			
Heterogeneity: Chi² = 0.65, df = 2 (P = 0.72); I² = 0%						
Test for overall effect: Z = 0.96 (P = 0.34)						
**Non smoker**						
COVID MED	1	5	0	1	4.1%	1.00 [0.06, 15.99]
REPLACE COVID	9	52	8	48	45.0%	1.04 [0.44, 2.47]
STAR-COVID	6	26	5	24	28.1%	1.11 [0.39, 3.16]
All other trials	0	17	0	14		-
**Subtotal (95% CI)**		**100**		**87**	**77.1%**	**1.06 [0.55, 2.03]**
Total events	16		13			
Heterogeneity: Chi² = 0.01, df = 2 (P = 0.99); I² = 0%						
Test for overall effect: Z = 0.18 (P = 0.86)						
**Total (95% CI)**		**136**		**132**	**100.0%**	**1.20 [0.69, 2.07]**
Total events	22		18			
Heterogeneity: Chi² = 1.24, df = 5 (P = 0.94); I² = 0%						
Test for overall effect: Z = 0.65 (P = 0.52)						
Test for subgroup differences: Chi² = 0.51, df = 1 (P = 0.47), I² = 0%						

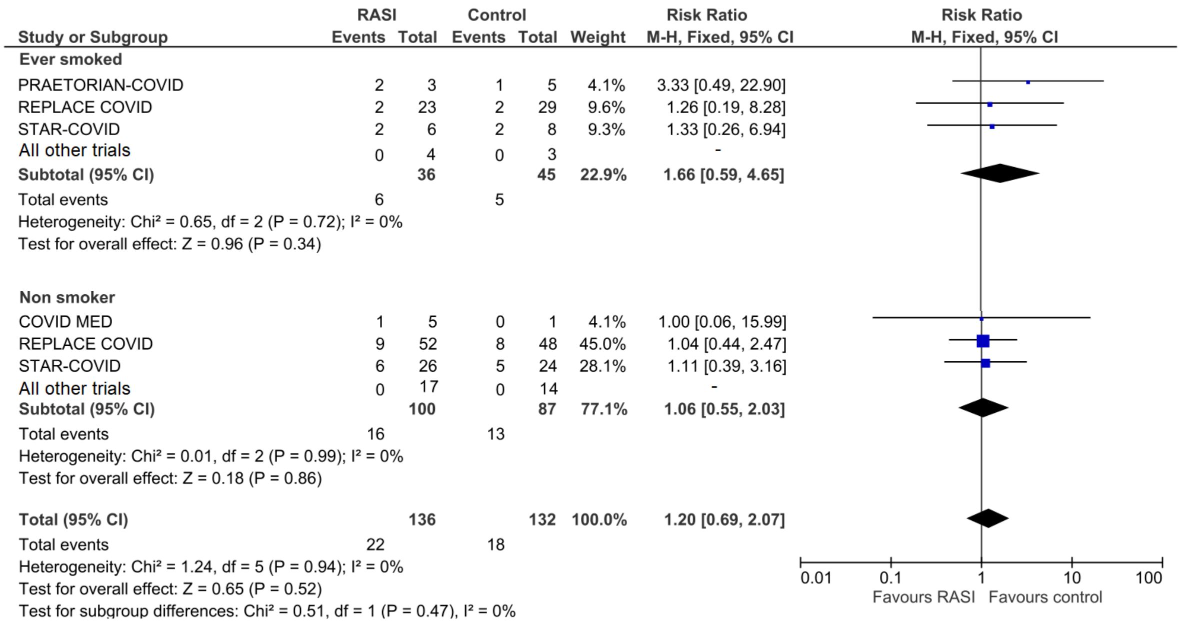

Figure S18: Meta-regression of loss to follow-up versus all-cause mortality

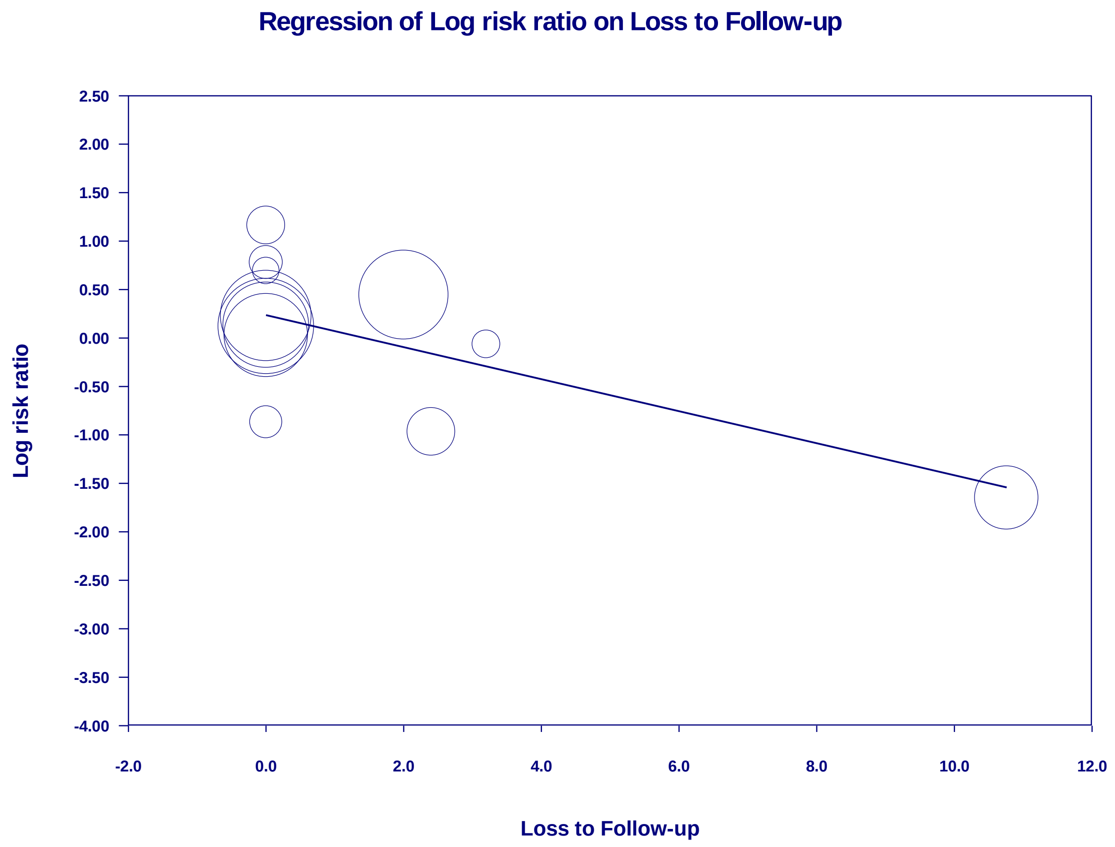

Figure S19: Network Meta-analysis comparing control vs ACEi or ARB – Mortality Risk Ratio with 95%CI.

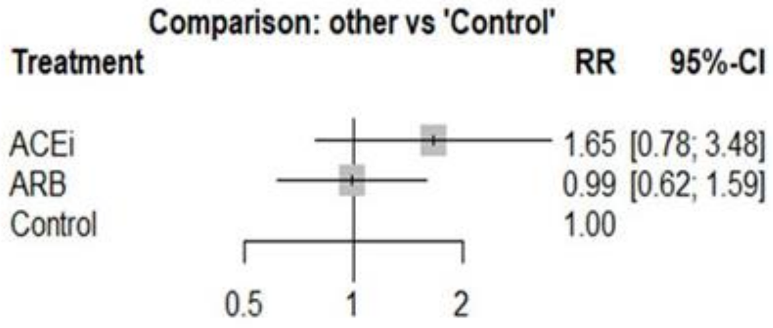

Figure S20: Network Meta-analysis comparing control, ACEi and ARB Rankogram Plot - Probability of having a specific rank.

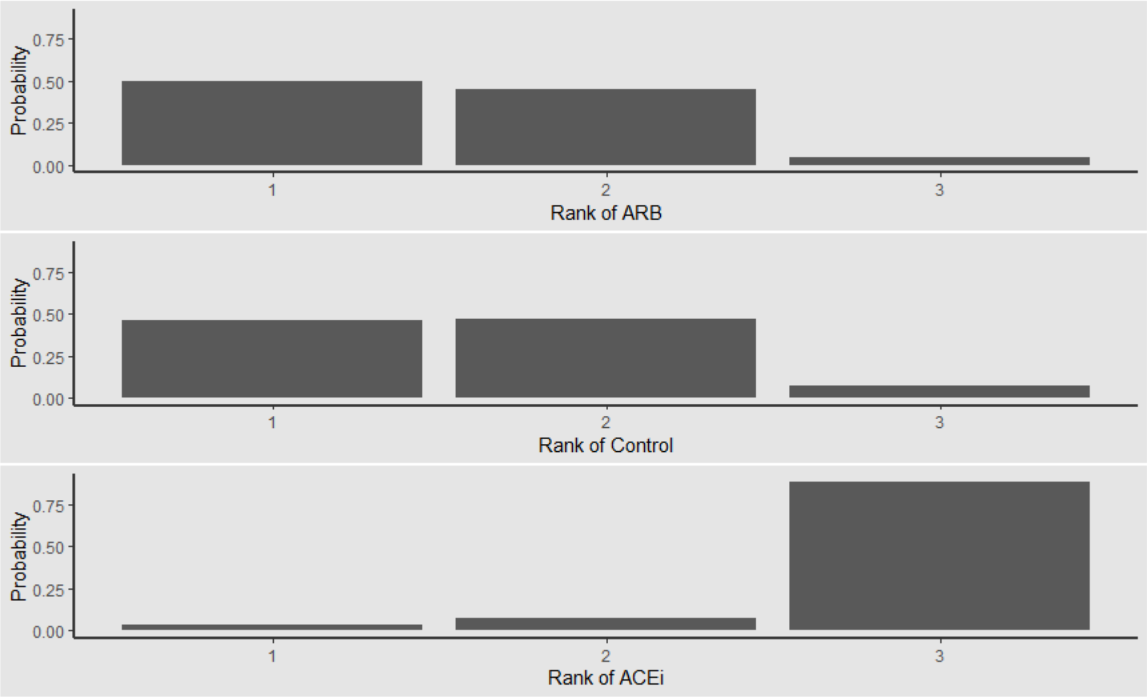

Figure S21: Cerebrovascular Events

Study or Subgroup	RASI Events	RASI Total	Control Events	Control Total	Weight	Risk Ratio M-H, Fixed, 95% CI
ACE-COVID	2	100	0	104	14.2%	5.20 [0.25, 106.95]
BRACE CORONA	3	325	3	334	85.8%	1.03 [0.21, 5.05]
All other trials	0	346	0	337		-
**Total (95% CI)**		**771**		**775**	**100.0%**	**1.62 [0.43, 6.15]**
Total events	5		3			

Heterogeneity: Chi² = 0.88, df = 1 (P = 0.35); I^2 = 0%
Test for overall effect: Z = 0.71 (P = 0.48)

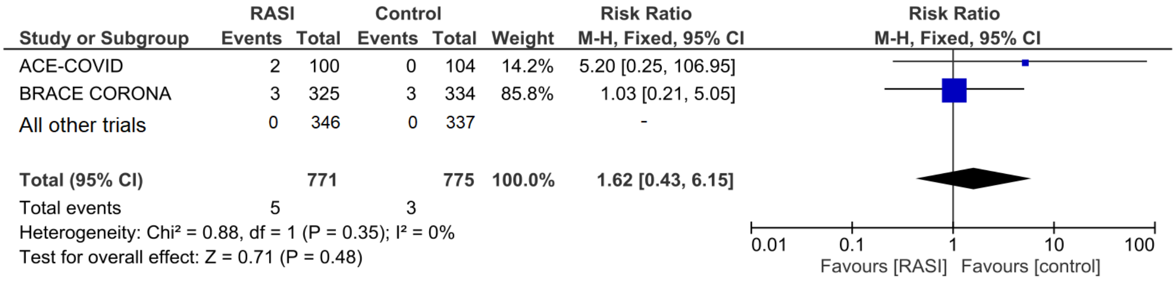

Figure S22: Congestive Cardiac Failure

(A) Random Effects

Study or Subgroup	RASI Events	RASI Total	Control Events	Control Total	Weight	Risk Ratio M-H, Random, 95% CI
BRACE CORONA	16	325	14	334	49.4%	1.17 [0.58, 2.37]
RAAS-COVID	1	25	7	21	27.6%	0.12 [0.02, 0.90]
REPLACE COVID	2	75	1	77	23.0%	2.05 [0.19, 22.17]
All other trials	0	245	0	239		–
**Total (95% CI)**		**670**		**671**	**100.0%**	**0.71 [0.16, 3.17]**
Total events	19		22			

Heterogeneity: Tau² = 1.05; Chi² = 4.96, df = 2 (P = 0.08); I² = 60%
Test for overall effect: Z = 0.45 (P = 0.66)

Risk Ratio
M-H, Random, 95% CI

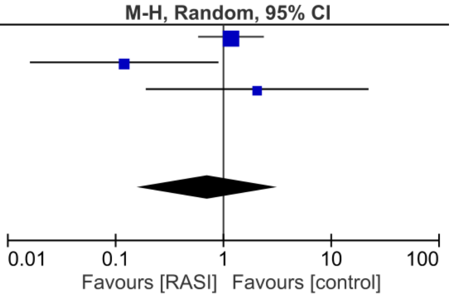

(B) Fixed Effects

Study or Subgroup	RASI Events	RASI Total	Control Events	Control Total	Weight	Risk Ratio M-H, Fixed, 95% CI
BRACE CORONA	16	325	14	334	61.6%	1.17 [0.58, 2.37]
RAAS-COVID	1	25	7	21	34.0%	0.12 [0.02, 0.90]
REPLACE COVID	2	75	1	77	4.4%	2.05 [0.19, 22.17]
All other trials	0	245	0	239		-
**Total (95% CI)**		**670**		**671**	**100.0%**	**0.86 [0.47, 1.54]**
Total events	19		22			

Heterogeneity: Chi² = 4.96, df = 2 (P = 0.08); I² = 60%
Test for overall effect: Z = 0.52 (P = 0.60)

Risk Ratio
M-H, Fixed, 95% CI

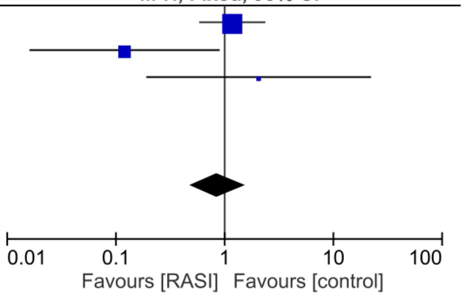

Figure S23: Venous Thromboembolism

Study or Subgroup	RASI Events	RASI Total	Control Events	Control Total	Weight	Risk Ratio M-H, Fixed, 95% CI
BRACE CORONA	4	325	6	334	77.7%	0.69 [0.20, 2.41]
COVID MED	1	9	0	3	9.4%	1.20 [0.06, 23.70]
REPLACE COVID	4	75	1	77	13.0%	4.11 [0.47, 35.90]
All othe trials	0	337	0	340		-
**Total (95% CI)**		**746**		**754**	**100.0%**	**1.18 [0.45, 3.05]**
Total events	9		7			

Heterogeneity: Chi² = 1.99, df = 2 (P = 0.37); I² = 0%

Test for overall effect: Z = 0.33 (P = 0.74)

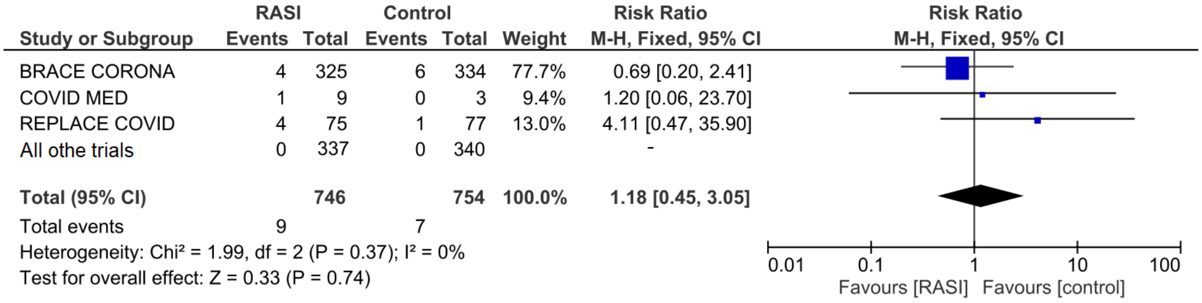

Figure S24: Hospitalisation

	RASI		Control			Risk Ratio
Study or Subgroup	Events	Total	Events	Total	Weight	M-H, Fixed, 95% CI
ALPS-COVID OP	3	58	1	59	32.2%	3.05 [0.33, 28.49]
COVERAGE France	3	36	2	33	67.8%	1.38 [0.24, 7.72]
**Total (95% CI)**		**94**		**92**	**100.0%**	**1.92 [0.50, 7.35]**
Total events	6		3			

Heterogeneity: Chi² = 0.31, df = 1 (P = 0.58); I² = 0%

Test for overall effect: Z = 0.95 (P = 0.34)

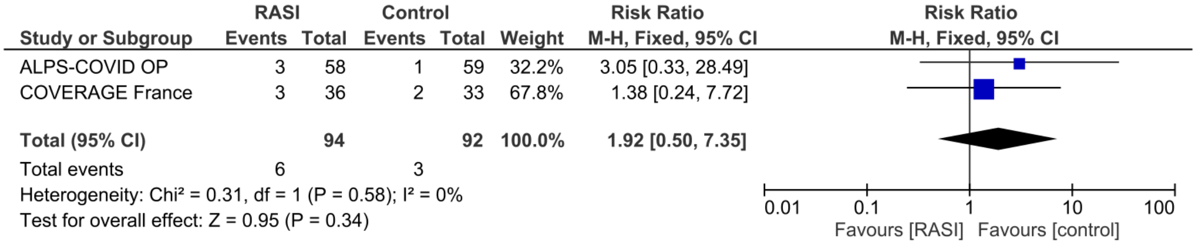

Figure S25: ICU admission –Start vs Continue/Discontinue Trials

Study or Subgroup	RASI Events	RASI Total	Control Events	Control Total	Weight	Risk Ratio M-H, Fixed, 95% CI
**Start**						
ALPS COVID IP	36	101	28	104	32.3%	1.32 [0.88, 2.00]
ALPS-COVID OP	1	58	1	59	1.2%	1.02 [0.07, 15.88]
ARB use to minimize respiratory failure	1	16	2	15	2.4%	0.47 [0.05, 4.65]
COVID MED	4	9	0	3	0.8%	3.60 [0.25, 52.60]
PRAETORIAN-COVID	1	11	2	12	2.2%	0.55 [0.06, 5.21]
Telmisartan for treatment of COVID-19	6	78	15	80	17.4%	0.41 [0.17, 1.00]
All other trials	0	36	0	33		-
**Subtotal (95% CI)**		**309**		**306**	**56.3%**	**1.00 [0.71, 1.42]**
Total events	49		48			
Heterogeneity: Chi² = 7.17, df = 5 (P = 0.21); I² = 30%						
Test for overall effect: Z = 0.01 (P = 0.99)						
**Continue/Discontinue**						
ACE-COVID	18	100	20	104	23.0%	0.94 [0.53, 1.66]
RAAS-COVID	0	25	1	21	1.9%	0.28 [0.01, 6.58]
REPLACE COVID	16	75	14	77	16.2%	1.17 [0.62, 2.23]
SWITCH-COVID	4	10	2	8	2.6%	1.60 [0.39, 6.62]
**Subtotal (95% CI)**		**210**		**210**	**43.7%**	**1.04 [0.69, 1.55]**
Total events	38		37			
Heterogeneity: Chi² = 1.28, df = 3 (P = 0.73); I² = 0%						
Test for overall effect: Z = 0.17 (P = 0.87)						
**Total (95% CI)**		**519**		**516**	**100.0%**	**1.02 [0.78, 1.32]**
Total events	87		85			
Heterogeneity: Chi² = 8.43, df = 9 (P = 0.49); I² = 0%						
Test for overall effect: Z = 0.12 (P = 0.90)						
Test for subgroup differences: Chi² = 0.01, df = 1 (P = 0.91), I² = 0%						

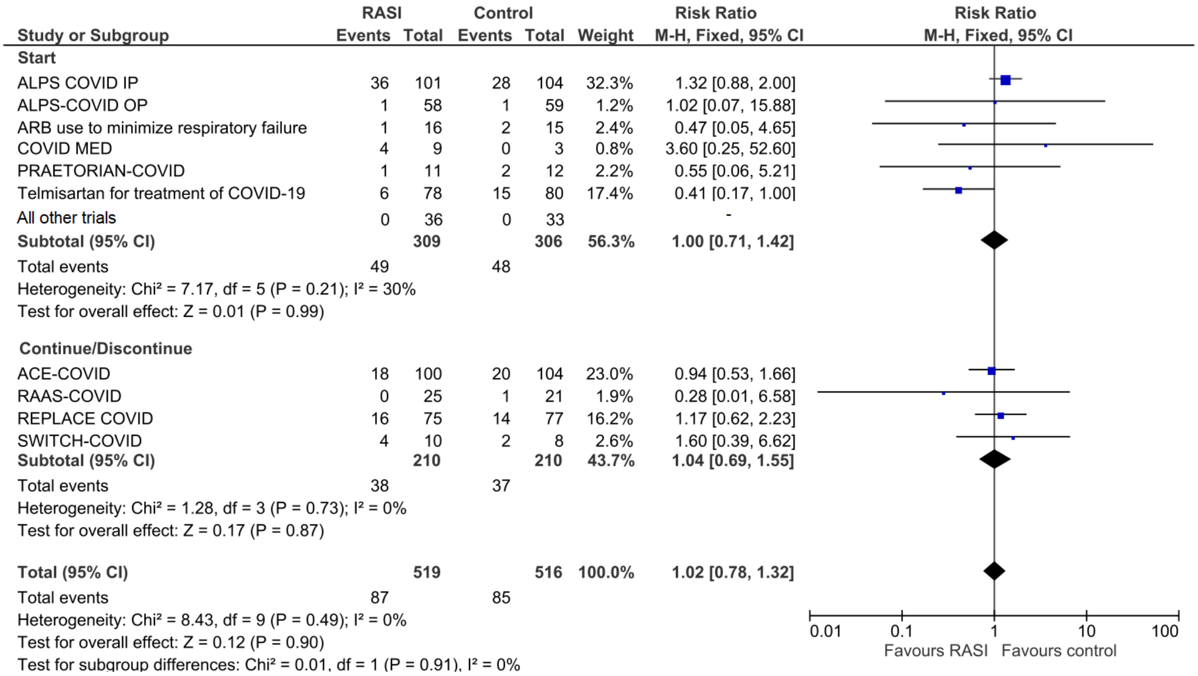

Figure S26: Mechanical Ventilation –Start vs Continue/Discontinue Trials

	RASI		Control			Risk Ratio
Study or Subgroup	Events	Total	Events	Total	Weight	M-H, Fixed, 95% CI
**Start**						
ALPS COVID IP	21	101	17	104	18.1%	1.27 [0.71, 2.27]
ARB use to minimize respiratory failure	1	16	1	15	1.1%	0.94 [0.06, 13.68]
COVID MED	4	9	0	3	0.8%	3.60 [0.25, 52.60]
Evaluation of the effects of losartan in COVID-19	8	41	9	39	10.0%	0.85 [0.36, 1.97]
PRAETORIAN-COVID	0	11	2	12	2.6%	0.22 [0.01, 4.07]
STAR-COVID	8	32	6	32	6.5%	1.33 [0.52, 3.41]
Telmisartan for treatment of COVID-19	4	78	4	80	4.3%	1.03 [0.27, 3.96]
All other trials	0	94	0	92		-
**Subtotal (95% CI)**		**382**		**377**	**43.3%**	**1.13 [0.77, 1.66]**
Total events	46		39			
Heterogeneity: Chi² = 2.71, df = 6 (P = 0.84); I² = 0%						
Test for overall effect: Z = 0.61 (P = 0.54)						
**Continue/Discontinue**						
ACE-COVID	8	100	10	104	10.6%	0.83 [0.34, 2.02]
BRACE CORONA	25	325	32	334	34.1%	0.80 [0.49, 1.32]
RAAS-COVID	1	25	2	21	2.3%	0.42 [0.04, 4.31]
REPLACE COVID	10	75	8	77	8.5%	1.28 [0.54, 3.07]
SWITCH-COVID	3	10	1	8	1.2%	2.40 [0.30, 18.89]
**Subtotal (95% CI)**		**535**		**544**	**56.7%**	**0.90 [0.62, 1.31]**
Total events	47		53			
Heterogeneity: Chi² = 2.14, df = 4 (P = 0.71); I² = 0%						
Test for overall effect: Z = 0.56 (P = 0.58)						
**Total (95% CI)**		**917**		**921**	**100.0%**	**1.00 [0.76, 1.31]**
Total events	93		92			
Heterogeneity: Chi² = 5.55, df = 11 (P = 0.90); I² = 0%						
Test for overall effect: Z = 0.02 (P = 0.99)						
Test for subgroup differences: Chi² = 0.69, df = 1 (P = 0.41), I² = 0%						

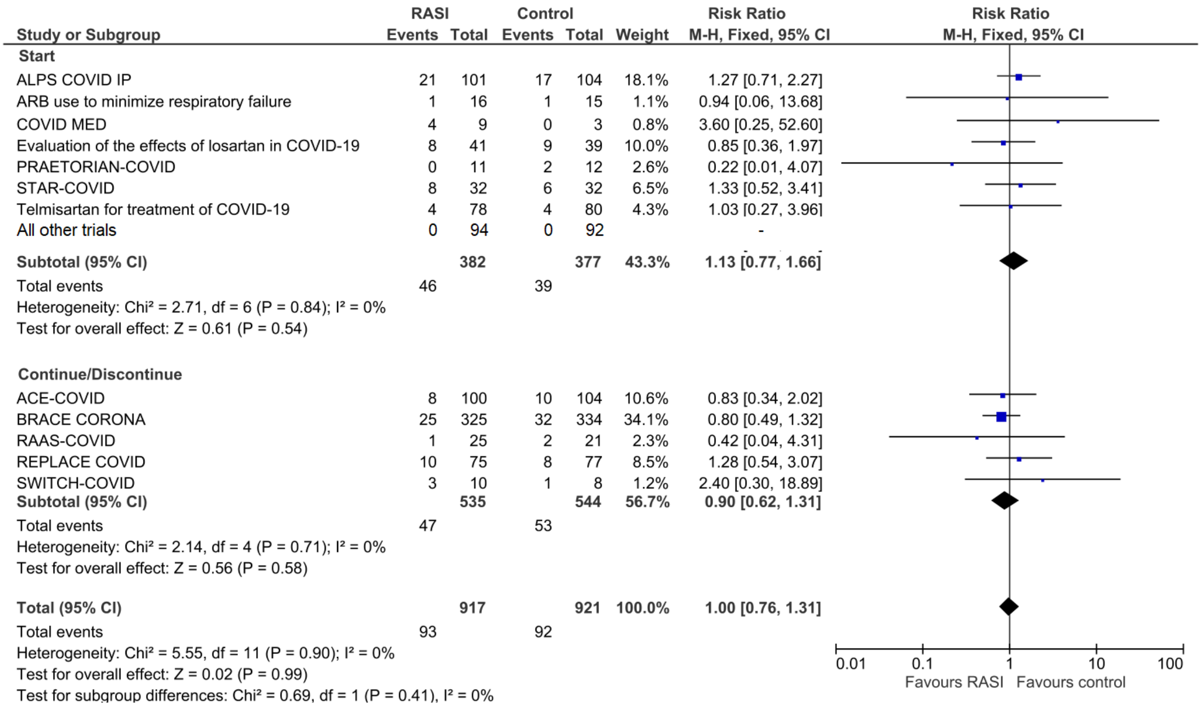

Figure S27: Hypotension requiring Inotropes by COVID-19 severity

Study or Subgroup	RASI Events	RASI Total	Control Events	Control Total	Weight	Risk Ratio M-H, Fixed, 95% CI
**Severe disease**						
ALPS COVID IP	11	22	6	21	22.3%	1.75 [0.79, 3.88]
COVID MED	3	5	0	1	2.7%	2.33 [0.19, 28.25]
REPLACE COVID	3	9	1	10	3.4%	3.33 [0.42, 26.58]
STAR-COVID	6	32	6	32	21.8%	1.00 [0.36, 2.77]
**Subtotal (95% CI)**		**68**		**64**	**50.3%**	**1.56 [0.88, 2.79]**
Total events	23		13			
Heterogeneity: Chi² = 1.42, df = 3 (P = 0.70); I² = 0%						
Test for overall effect: Z = 1.52 (P = 0.13)						
**Moderate**						
ALPS COVID IP	8	79	7	83	24.8%	1.20 [0.46, 3.16]
REPLACE COVID	3	28	2	25	7.7%	1.34 [0.24, 7.38]
All other trials	0	12	0	9		-
**Subtotal (95% CI)**		**119**		**117**	**32.5%**	**1.23 [0.53, 2.86]**
Total events	11		9			
Heterogeneity: Chi² = 0.01, df = 1 (P = 0.91); I² = 0%						
Test for overall effect: Z = 0.49 (P = 0.62)						
**Mild**						
REPLACE COVID	3	38	5	42	17.3%	0.66 [0.17, 2.59]
All other trials	0	60	0	60		
**Subtotal (95% CI)**		**98**		**102**	**17.3%**	**0.66 [0.17, 2.59]**
Total events	3		5			
Heterogeneity: Not applicable						
Test for overall effect: Z = 0.59 (P = 0.55)						
**Total (95% CI)**		**285**		**283**	**100.0%**	**1.30 [0.83, 2.04]**
Total events	37		27			
Heterogeneity: Chi² = 2.76, df = 6 (P = 0.84); I² = 0%						
Test for overall effect: Z = 1.15 (P = 0.25)						
Test for subgroup differences: Chi² = 1.34, df = 2 (P = 0.51), I² = 0%						

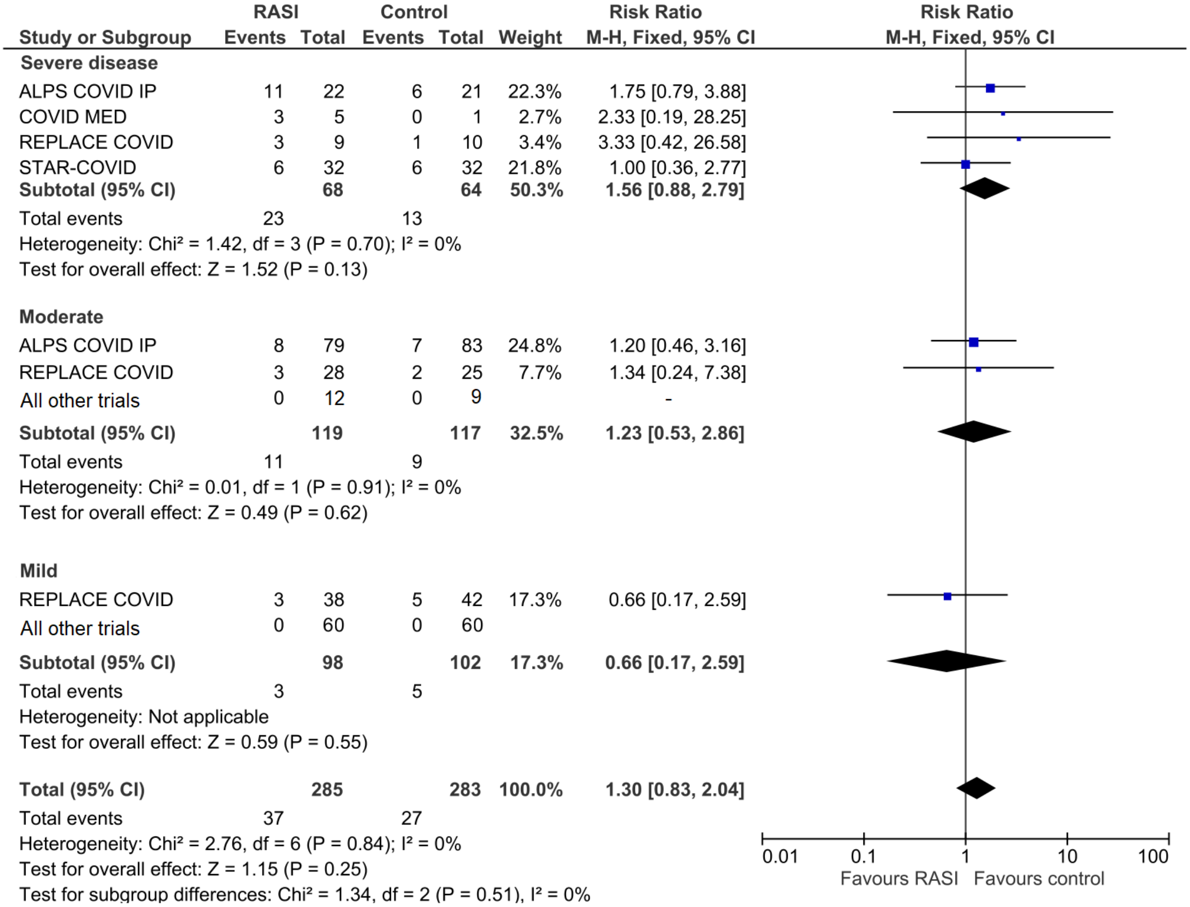

Figure S28: Inotropes – Start vs Continue/Discontinue Trials

Study or Subgroup	RASI Events	RASI Total	Control Events	Control Total	Weight	Risk Ratio M-H, Fixed, 95% CI
**Start**						
ALPS COVID IP	19	101	13	104	20.4%	1.50 [0.79, 2.88]
COVID MED	3	9	0	3	1.1%	2.80 [0.18, 42.80]
STAR-COVID	6	32	6	32	9.5%	1.00 [0.36, 2.77]
All other trials	0	172	0	172		-
**Subtotal (95% CI)**		**236**		**231**	**31.0%**	**1.40 [0.82, 2.39]**
Total events	28		19			
Heterogeneity: Chi² = 0.71, df = 2 (P = 0.70); I² = 0%						
Test for overall effect: Z = 1.23 (P = 0.22)						
**Continue/Discontinue**						
ACE-COVID	4	100	8	104	12.5%	0.52 [0.16, 1.67]
BRACE CORONA	23	325	28	334	43.9%	0.84 [0.50, 1.43]
REPLACE COVID	9	75	8	77	12.6%	1.16 [0.47, 2.83]
All other trials	0	10	0	8		-
**Subtotal (95% CI)**		**510**		**523**	**69.0%**	**0.84 [0.55, 1.28]**
Total events	36		44			
Heterogeneity: Chi² = 1.13, df = 2 (P = 0.57); I² = 0%						
Test for overall effect: Z = 0.80 (P = 0.43)						
**Total (95% CI)**		**746**		**754**	**100.0%**	**1.01 [0.73, 1.41]**
Total events	64		63			
Heterogeneity: Chi² = 3.75, df = 5 (P = 0.59); I² = 0%						
Test for overall effect: Z = 0.09 (P = 0.93)						
Test for subgroup differences: Chi² = 2.12, df = 1 (P = 0.15), I² = 52.8%						

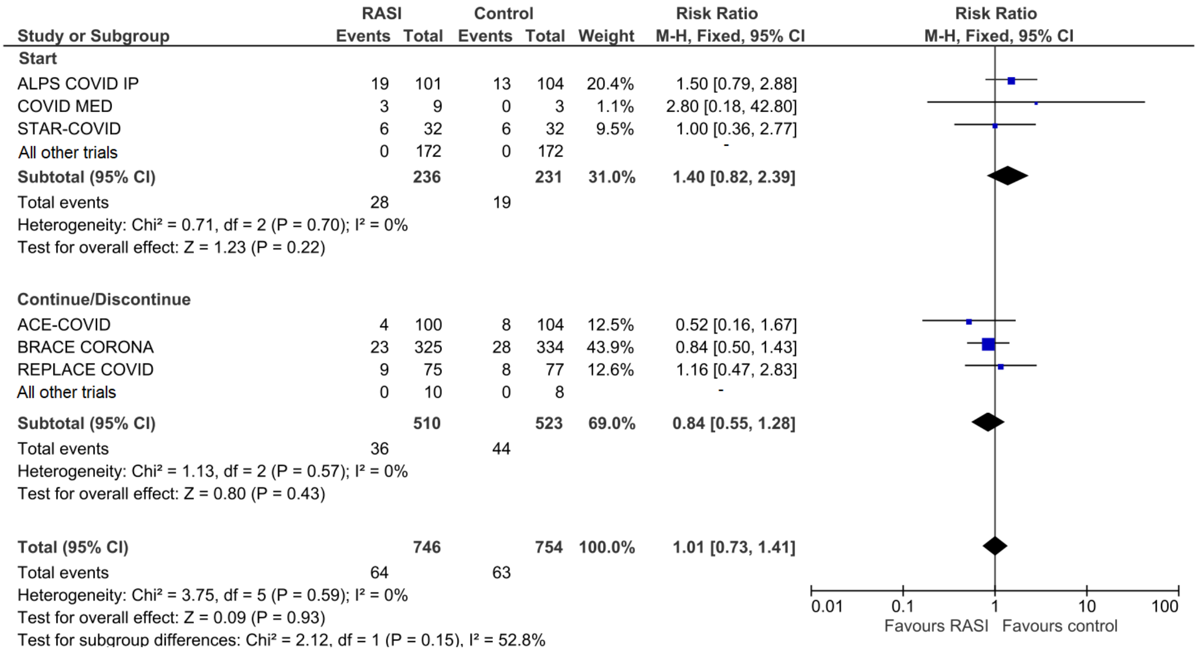

Figure S29: Acute Kidney Injury – Start vs Continue/Discontinue Trials

Study or Subgroup	RASI Events	RASI Total	Control Events	Control Total	Weight	Risk Ratio M-H, Fixed, 95% CI
**Start**						
ALPS COVID IP	19	101	11	104	61.7%	1.78 [0.89, 3.55]
COVID MED	1	9	0	3	4.1%	1.20 [0.06, 23.70]
STAR-COVID	5	32	2	32	11.4%	2.50 [0.52, 11.96]
All other trials	0	94	0	92		-
**Subtotal (95% CI)**		**236**		**231**	**77.2%**	**1.85 [1.00, 3.44]**
Total events	25		13			
Heterogeneity: Chi² = 0.24, df = 2 (P = 0.89); I² = 0%						
Test for overall effect: Z = 1.96 (P = 0.05)						
**Continue/Discontinue**						
ACE-COVID	1	100	0	104	2.8%	3.12 [0.13, 75.67]
REPLACE COVID	3	75	3	77	16.9%	1.03 [0.21, 4.93]
SWITCH-COVID	2	10	0	8	3.1%	4.09 [0.22, 74.78]
All other trials	0	25	0	21		-
**Subtotal (95% CI)**		**210**		**210**	**22.8%**	**1.70 [0.51, 5.74]**
Total events	6		3			
Heterogeneity: Chi² = 0.89, df = 2 (P = 0.64); I² = 0%						
Test for overall effect: Z = 0.86 (P = 0.39)						
**Total (95% CI)**		**446**		**441**	**100.0%**	**1.82 [1.05, 3.16]**
Total events	31		16			
Heterogeneity: Chi² = 1.16, df = 5 (P = 0.95); I² = 0%						
Test for overall effect: Z = 2.13 (P = 0.03)						
Test for subgroup differences: Chi² = 0.01, df = 1 (P = 0.90), I² = 0%						

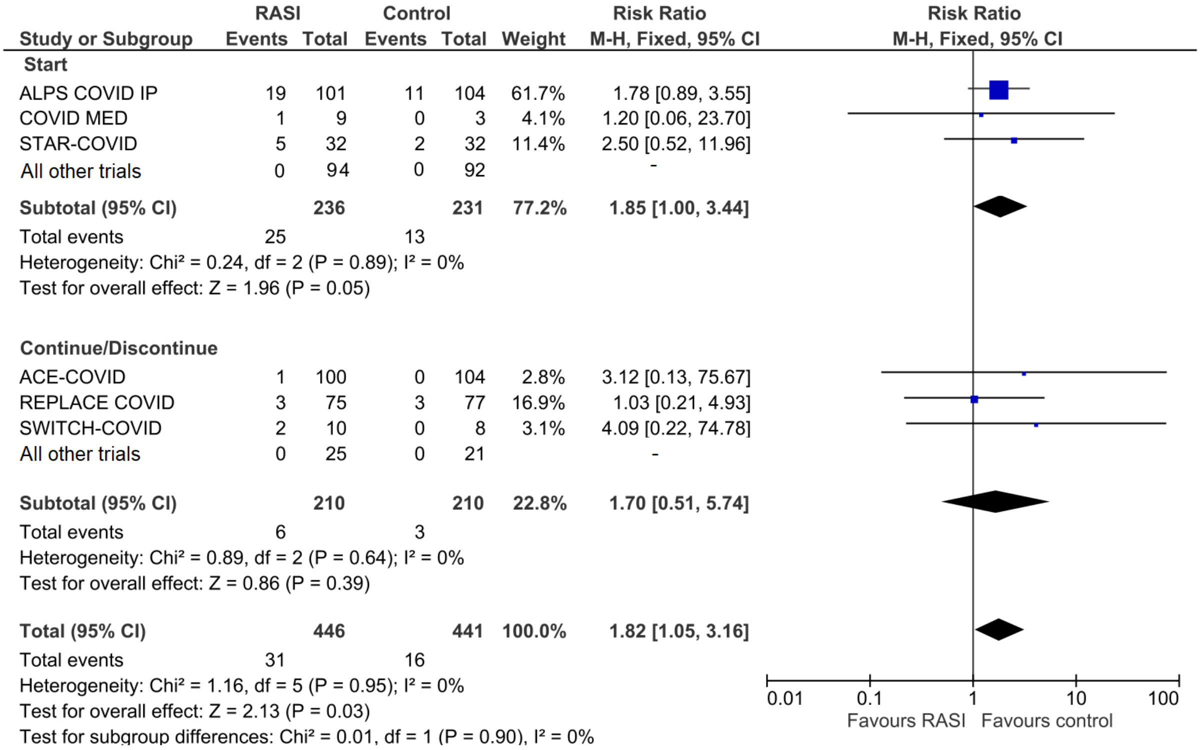

Figure S30: Acute Kidney Injury – Severity of COVID-19

Study or Subgroup	RASI Events	RASI Total	Control Events	Control Total	Weight	Risk Ratio M-H, Fixed, 95% CI
**Severe disease**						
ALPS COVID IP	6	22	4	20	23.9%	1.36 [0.45, 4.14]
COVID MED	1	5	0	1	4.3%	1.00 [0.06, 15.99]
REPLACE COVID	2	9	2	10	10.8%	1.11 [0.19, 6.34]
STAR-COVID	5	32	2	32	11.4%	2.50 [0.52, 11.96]
**Subtotal (95% CI)**		**68**		**63**	**50.3%**	**1.54 [0.71, 3.31]**
Total events	14		8			
Heterogeneity: Chi² = 0.64, df = 3 (P = 0.89); I² = 0%						
Test for overall effect: Z = 1.10 (P = 0.27)						
**Moderate**						
ALPS COVID IP	13	79	7	83	38.9%	1.95 [0.82, 4.64]
REPLACE COVID	0	25	1	28	8.1%	0.37 [0.02, 8.73]
All other trials	0	14	0	10		-
**Subtotal (95% CI)**		**118**		**121**	**47.0%**	**1.68 [0.74, 3.79]**
Total events	13		8			
Heterogeneity: Chi² = 0.99, df = 1 (P = 0.32); I² = 0%						
Test for overall effect: Z = 1.25 (P = 0.21)						
**Mild**						
REPLACE COVID	1	38	0	42	2.7%	3.31 [0.14, 78.84]
All other trials	0	66	0	68		-
**Subtotal (95% CI)**		**104**		**110**	**2.7%**	**3.31 [0.14, 78.84]**
Total events	1		0			
Heterogeneity: Not applicable						
Test for overall effect: Z = 0.74 (P = 0.46)						
**Total (95% CI)**		**290**		**294**	**100.0%**	**1.65 [0.95, 2.86]**
Total events	28		16			
Heterogeneity: Chi² = 1.89, df = 6 (P = 0.93); I² = 0%						
Test for overall effect: Z = 1.79 (P = 0.07)						
Test for subgroup differences: Chi² = 0.22, df = 2 (P = 0.90), I² = 0%						

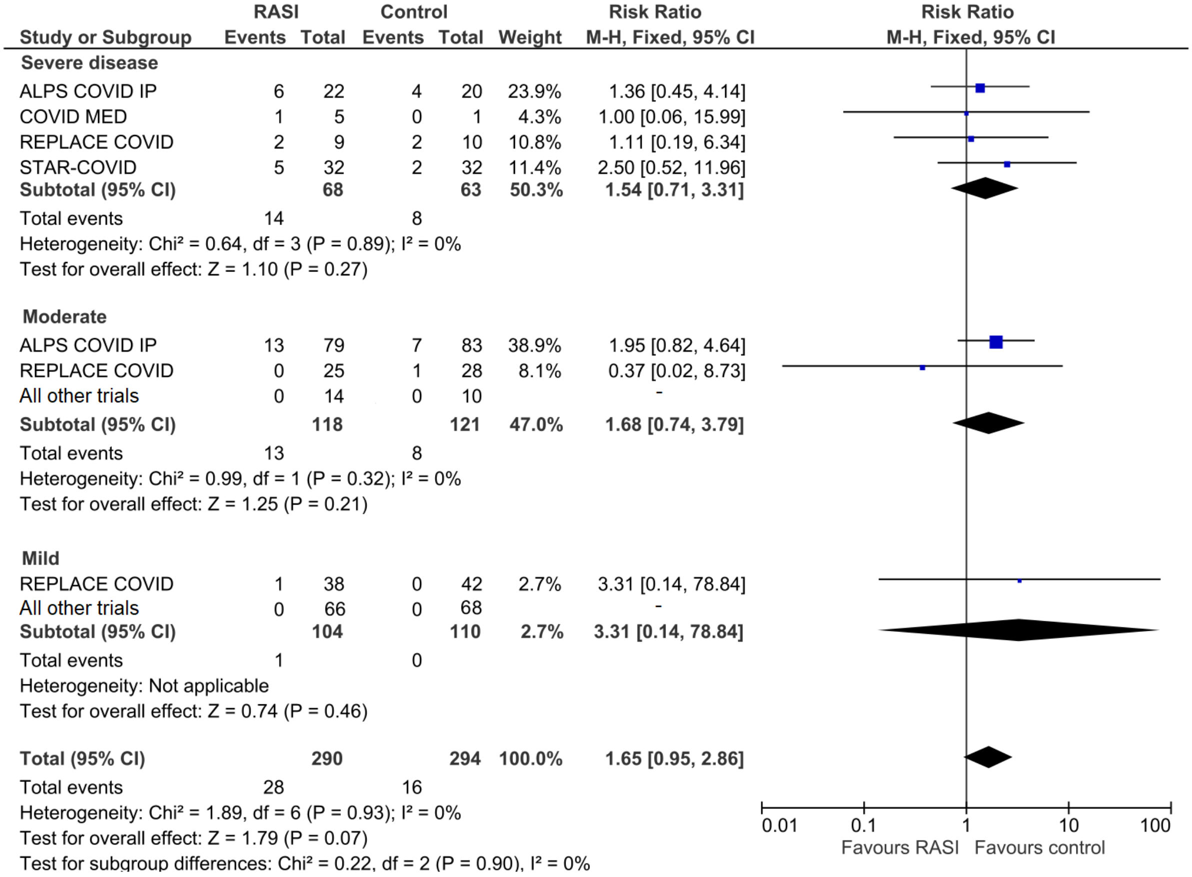

Supplement: Supplementary file 1 — Data S1 Tables S1–S2 Figures S1–S30 [file JAH3-11-e026143-s001.pdf]
